# Mapping the sequence specificity of heterotypic amyloid interactions enables the identification of aggregation modifiers

**DOI:** 10.1038/s41467-022-28955-9

**Published:** 2022-03-15

**Authors:** Nikolaos Louros, Meine Ramakers, Emiel Michiels, Katerina Konstantoulea, Chiara Morelli, Teresa Garcia, Nele Moonen, Sam D’Haeyer, Vera Goossens, Dietmar Rudolf Thal, Dominique Audenaert, Frederic Rousseau, Joost Schymkowitz

**Affiliations:** 1 Switch Laboratory, VIB Center for Brain and Disease Research, Herestraat 49, 3000 Leuven, Belgium; 2 Switch Laboratory, Department of Cellular and Molecular Medicine, KU Leuven, Herestraat 49, 3000 Leuven, Belgium; 3 VIB Screening Core, Ghent, Belgium; 4 Centre for Bioassay Development and Screening (C-BIOS), Ghent University, Ghent, Belgium; 5 KU Leuven, Leuven Brain Institute, 3000 Leuven, Belgium; 6 Laboratory for Neuropathology, KU Leuven, and Department of Pathology, UZ Leuven, 3000 Leuven, Belgium

**Keywords:** Thermodynamics, Protein aggregation, Protein aggregation, Neurodegeneration, Molecular modelling

## Abstract

Heterotypic amyloid interactions between related protein sequences have been observed in functional and disease amyloids. While sequence homology seems to favour heterotypic amyloid interactions, we have no systematic understanding of the structural rules determining such interactions nor whether they inhibit or facilitate amyloid assembly. Using structure-based thermodynamic calculations and extensive experimental validation, we performed a comprehensive exploration of the defining role of sequence promiscuity in amyloid interactions. Using tau as a model system we demonstrate that proteins with local sequence homology to tau amyloid nucleating regions can modify fibril nucleation, morphology, assembly and spreading of aggregates in cultured cells. Depending on the type of mutation such interactions inhibit or promote aggregation in a manner that can be predicted from structure. We find that these heterotypic amyloid interactions can result in the subcellular mis-localisation of these proteins. Moreover, equilibrium studies indicate that the critical concentration of aggregation is altered by heterotypic interactions. Our findings suggest a structural mechanism by which the proteomic background can modulate the aggregation propensity of amyloidogenic proteins and we discuss how such sequence-specific proteostatic perturbations could contribute to the selective cellular susceptibility of amyloid disease progression.

## Abstract (web summary)

In this work, Louros et al. uncover a rule book for interactions of amyloids with other proteins. This grammar was shown to promote cellular spreading of tau aggregates in cells, but can also be harvested to develop structure-based aggregation blockers.

## Introduction

Neurodegenerative disorders are a diverse group of pathologies that are associated to the gradual deterioration of different brain regions and cause variable clinical phenotypes that range from cognitive impairment to motor deterioration and neuropsychiatric symptoms^1,2^. Despite this complexity, these diseases share fundamental characteristics in regard to their mechanistic underpinnings and clinical manifestation. To begin with, they are characterised by the presence of β-rich amyloid aggregates, the formation of which is initiated by self-propagation of the amyloid conformation of certain key proteins and affects particular areas of the brain^3–8^. Another shared clinical feature relates to their specific spatial and temporal progression patterns that can discriminate between distinct disorders by matching symptoms to the functionality of the affected brain regions^9–11^. Efforts to address the basis of cellular and regional vulnerability have focused on the intricate balance between intrinsic neuronal homeostasis to the heterogeneity of amyloid self-assembly and transcellular propagation pathways^9,12^. Genetic variability^13,14^, extrinsic clearance pathways^15^ and molecular expression profiles^16,17^ are important risk factors that enhance cellular susceptibility to toxic amyloid aggregates, with their effects being further exacerbated when coupled to the progressive decline of molecular proteostasis mechanisms that deteriorate with physiological ageing^18^. Although the exact cellular interactions that contribute to the modulation of neuronal susceptibility still remain largely unknown, the prominent role of cellular proteomic heterogeneity in this process can no longer ignored^19^. Specific protein hetero-interactions have been shown to directly influence susceptibility to amyloid formation of several proteins, including among others, Aβ^20–22^, tau^23,24^ and α-synuclein^25–27^, involved in Alzheimer’s (AD) and Parkinson’s disease (PD), respectively. In the same line, cell-specific inherent metastability of proteins that supersede their solubility levels has been proposed as a generic mechanism that can promote regional protein co-deposition^28–32^.

Cellular predilection to toxic aggregates is also conformation-specific, as recent evidence has shown that different amyloid fibril morphologies derived from the same misfolded protein can characterise other neurodegenerative disorders^33–35^. Regardless of their protein of origin and self-assembly conditions, however, amyloid fibrils share a common structural cross-β architecture^36–39^. Further to this, disease-related amyloid conformers share overlapping thermodynamic distribution profiles, as specific segments that also drive their nucleation predominantly stabilise their amyloid framework^40^. These regions, previously identified as aggregation prone regions (APRs)^41–45^, form thermodynamically stable steric zipper interfaces that staple together amyloid fibril structures. As a result, they are also able to support their own self-assembly^46–50^, as well as to promote heterotypic interactions dominated by sequence similarity^19,51–55^ that have been shown to promote pathology^56–59^ or the formation of biologically functional amyloids^60–66^.

Several classes of biomolecules have been found to interact with amyloid fibrils. Glycosaminoglycans, RNA, lipids and rotor dyes, among others, selectively interact with binding pockets or other surface features of amyloid polymorphs^67–70^, while chaperones have been shown to bind to the lateral surface of amyloid fibrils during secondary nucleation or fragmentation^71^. Heterotypic interactions between amyloids and proteins have also been found to modify elongation at the growing tips of amyloids suggesting the existence of cross-seeding in yeast prions^72^, functional amyloids^63,73^ and disease amyloids^48^. While heterotypic amyloid protein interactions have been observed in different model systems, we still have no understanding on their determining structural rules beyond the observation that sequence homology favours heterotypic amyloid interactions. Based on our growing insight into amyloid architecture, however, it is becoming evident that amyloid fibril structure is highly ordered and constrained by specific patterns of tightly interlaced side-chains, which are particularly susceptible to minimal variation. For example, even single disease mutations typically induce significant morphological differentiation that often raises barriers of structural incompatibility between strains^74–76^.

Here we focused on investigating sequence promiscuity of amyloid core APRs, as a structural mechanism that engages in heterotypic amyloid interactions. By performing a comprehensive thermodynamic evaluation of the entire sequence space for APR cores derived from several amyloidogenic proteins, we investigated the sequence dependencies that drive heterotypic interactions both in vitro and within a cellular environment. Together, our results highlight that this structural mechanism may be implicated in selective cellular vulnerability by utilising local sequence similarity to promote the entrapment of protein components with important functions, but can also be harvested as the means to improve therapeutics against major amyloid diseases.

## Results

### Thermodynamic profiling of heterotypic amyloid interactions

**Fig. 1:**
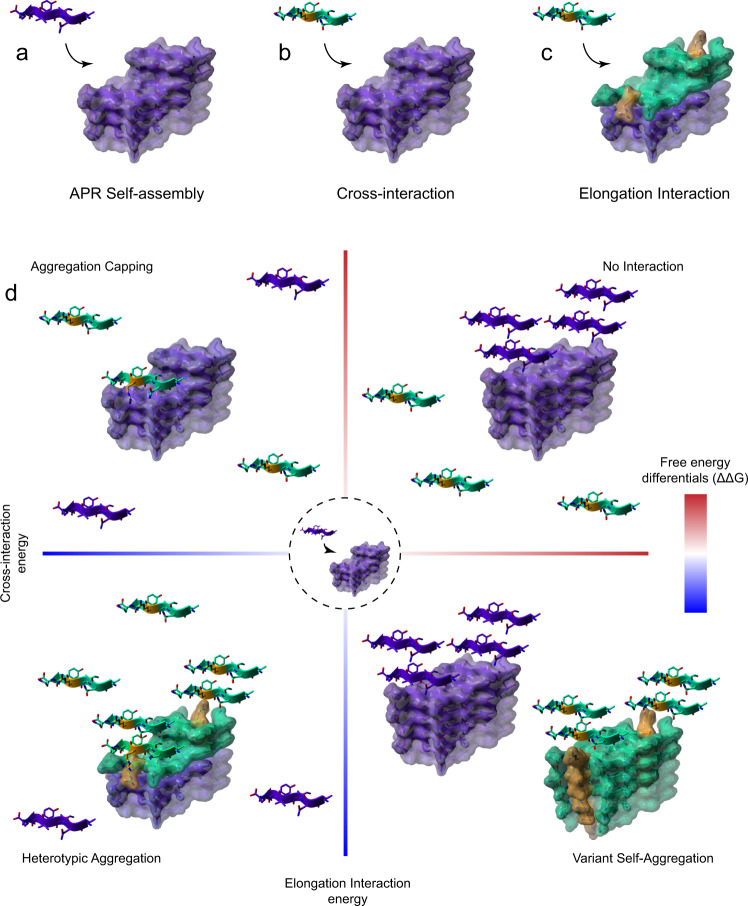
Structural framework to describe cross-interactions of APR aggregation cores. **a**–**c** Three structural templates were generated for each APR amyloid core structure (shown in purple), corresponding to **a** self-elongation by monomeric APR addition (purple β-strands), **b** primary cross-interaction of a single position (highlighted in yellow) sequence variant (shown as green β-strands) at the amyloid fibril ends and **c** successive elongation by the same variant. **d** Variants that produce favourable differentials compared to monomeric APR elongation are driven towards heterotypic aggregation, compared to disfavourable potentials that limit interactions. Aggregation capping is instigated by sequences that are compatible to cross-interactions with the APR core but block further elongation, while opposite energies are associated to individual self-aggregation, respectively.

In order to perform a systematic in silico exploration of the potential impact of the incorporation of homologous sequence segments from unrelated proteins into the amyloid core, we assembled a collection of 83 experimentally determined APR amyloid core structures derived from 18 distinct proteins (Supplementary Table 1). Using this dataset, we used the all-atom force field FoldX to perform an exhaustive thermodynamic profiling of the energies for cross-interaction (i.e. binding of a homologous sequence segment on the growing fibril tip) and elongation (i.e. docking of additional copies of the homologous sequence) against sequence divergence (Fig. 1a–c). We limited our search to single variants of the known major APRs reasoning that: (i) APRs are the kinetic drivers that promote self-assembly of amyloids^41–44^, (ii) individual amyloid polymorphs share energetic profiles in a sense that they depend on APRs as a common framework of high structural stability to counteract longer regions of structural frustration in their core^40^ and (iii) this approach also supports a deeper understanding of potential tendencies, as assignments are performed at a single residue level.

We developed a systematic thermodynamic analysis of the impact of side-chain mismatches on the cross-interaction and elongation energies in APR cross-beta assemblies, in order to classify APR-homologous sequences by their ability to interact with the APR in the amyloid structure, potentially giving rise to different heterotypic-induced outcomes (see below and Fig. 1d). Our approach is based on all-atom structural models of the cross-beta cores formed by the APR regions under study, in which we introduce mismatches and judge their impact on the thermodynamics using the FoldX force field (Fig. S1)^77^. By comparing the free energy of cross-interaction (Fig. 1b) and elongation (Fig. 1c) interactions to the free energy potential of the APR self-interaction (Fig. 1a), we can define hetero-interaction-compatible variants as sequences that produce thermodynamically favourable cross-interaction free energies at the growing tip of amyloid fibrils. Furthermore, elongation energies are used to distinguish segments that participate in heterotypic assembly (after docking, the fibril can grow further, Fig. 1d, bottom-left quadrant) from aggregation-blockers (after docking, further growth is not possible, also defined here as “cappers”) (Fig. 1d, top-left quadrant). On the other hand, favourable elongation energies and disfavourable cross-interaction energies suggest a propensity of the variant sequence towards its own intermolecular assembly, thus are expected to result in sequences that self-aggregate instead of cross-aggregate with the starting APR (Fig. 1d, bottom-right quadrant). Finally, limited aggregation propensity is expected for stretches that produce unsuited free energy profiles for both modes of interaction (Fig. 1d, top-right quadrant).

### Investigating sequence space compatibility of APR cross-interactions

**Fig. 2:**
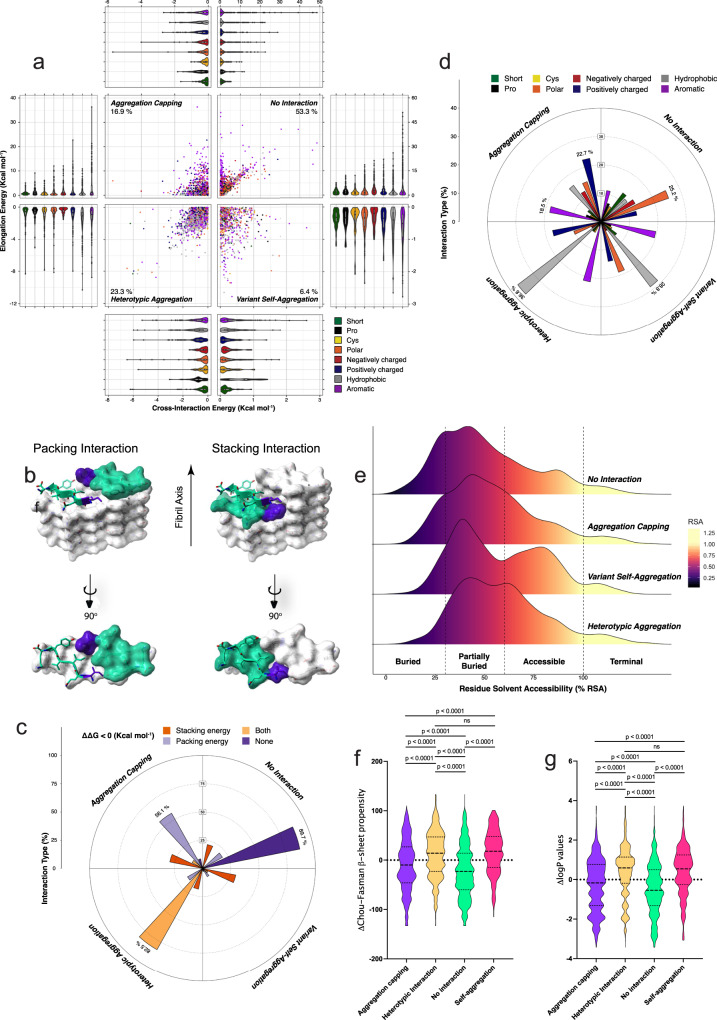
Thermodynamic profiling of APR amyloid core cross-interactions to single variants. **a** Single-mutation potential distributions in the four defined modes of cross-interaction (*n* = 10,374 mutations). A quadrant plot is generated by plotting cross-interaction energies on the x-axis and elongation energies on the y-axis, respectively. Energy distributions for each quadrant are shown as violin plots (consisting of a rotated kernel density plot on each side, so the full distribution can be observed), comprising also box-plots (representing median values with the lower and upper hinges corresponding to the 25th and 75th percentiles and whiskers representing minimum and maximum values). Residues are categorised as shown in the figure legend (Short = A, G, Pro = P, Cys = C, Polar = N, Q, S, T, Negatively charged = D, E, Positively charged = R, K, H, Hydrophobic = V, I, L, M, Aromatic = F, Y, W). **b**, **c** Rose plot (I.e. a circularly arranged histogram bar graph) distribution of the packing and stacking energy contributions along the four modes of interaction. **d** Rose plot distribution of residue type mutations along the four modes of interaction. **e** Residue burial distributions (relative surface area – RSA) for the four modes of interaction. **f** β-propensity and **g** solubility differentials calculated as a difference in value compared to their corresponding cognate APR sequence. Statistics: one-way ANOVA with multiple comparison. Source data are provided as a Source Data file.

Using our profiling scheme, we investigated the structural compatibility for heterotypic amyloid interactions of the entire collection of single variants of the APR dataset (Supplementary Table 1). Our energetic analysis revealed that less than 1 out of 4 variants are expected to engage in both cross-interaction and elongation, i.e. co-aggregation (Fig. 2a, bottom-left quadrant), while even fewer sequences (16.9%) were seen to be compatible with suppressing further elongation after cross-reacting with growing fibril ends, i.e. inhibiting aggregation (Fig. 2a, top-left quadrant). This apparent incompatibility of APR cores to sequence variation was also supported by the fact that only a limited fraction of sequence variants were predicted to favour self-assembly (6.9%) (Fig. 2a, bottom-right quadrant), possibly suggesting that the template backbone arrangements are strongly adapted to their particular sequences. In agreement, increasing the sequence variation to two mismatches further reduced the predicted thermodynamic compatibility, with predictions rendering most homologous stretches containing two mismatches (>75–80%) structurally incompatible for cross-interactions. However, the widened range of elongation potentials suggests that incorporation of multiple mutations can be a strategy to produce stronger cappers, in the expense of having to search a larger sequence space for sequences with compatible cross-interaction energies (Fig. S2). This is also supported by our previous findings on the cross-reactivity of sequence-targeting engineered antiviral and antibacterial peptide designs^78,79^, which also indicated that two mismatches are sufficient to inhibit cross-seeding.

We identified a linear correlation between cross-interaction and elongation free energy potentials for the majority of sequence variants ranging from heterotypic aggregation to non-interactors (Fig. 2a). This suggests that selective deviation from this correlation is required in order to develop potent capper designs that efficiently recognise fibril tips and simultaneously disrupt further elongation steps (i.e. strong cross-interaction energies with unfavourable elongation energies). This becomes more evident when comparing cross-sectional packing (residues opposing each other in the same layer) to transversal stacking (residues on top of each in subsequent layers) contributions along the axis of the fibril (Fig. 2b). Indeed, almost 90% of unfavourable interactions are characterised by poor packing and stacking energies, with both interfaces contributing actively for a similar fraction of heterotypic interactors (Fig. 2c). Notably, heterotypic capping was found to be facilitated primarily by destabilised packing interfaces during elongation (56.1%), suggesting that although stacking interactions are integral for overall stabilisation, cross-sectional packing is more easily destabilised by sequence variation.

Residue distribution analysis pinpointed that hydrophobic side chain substitutions are primarily associated to heterotypic aggregation, however they can also often increase the self-association tendency of variants, leading to independent self-assembly (Fig. 2a, d). On the other hand, polar side chain substitutions and introduction of so-called gatekeeper residues, such as Pro, Glu and Asp reduce hetero-compatibility, implying that apart of acting as evolutionary suppressors of APRs^80^, these residues may also limit aggregation cross-talk. Besides this, introduction of aromatic and positively charged residues was primarily associated to weak elongation energies. Heterotypic interactions were predominantly associated to partially buried positions, as changes in residues that are tightly packed in the amyloid core were harder to incorporate in co-aggregation compatible variants (Fig. 2e). In contrast, high surface exposure reduces specificity and can often increase the self-assembly potential of variants by simultaneously minimising cross-interactions. Finally, β-propensity (Fig. 2f) and solubility (Fig. 2g) are additional determinants between the four modes of interaction. Mutations that either promoted heterotypic or homotypic assembly were usually associated to increased β-sheet propensity and solubility, compared to their APR counterpart. On the other hand, heterotypic cappers are less soluble and often destabilise β-formation, with the effect being even stronger in the case of non-interacting mutants, respectively.

### Dimensionality reduction reveals the driving forces of heterotypic aggregation

**Fig. 3:**
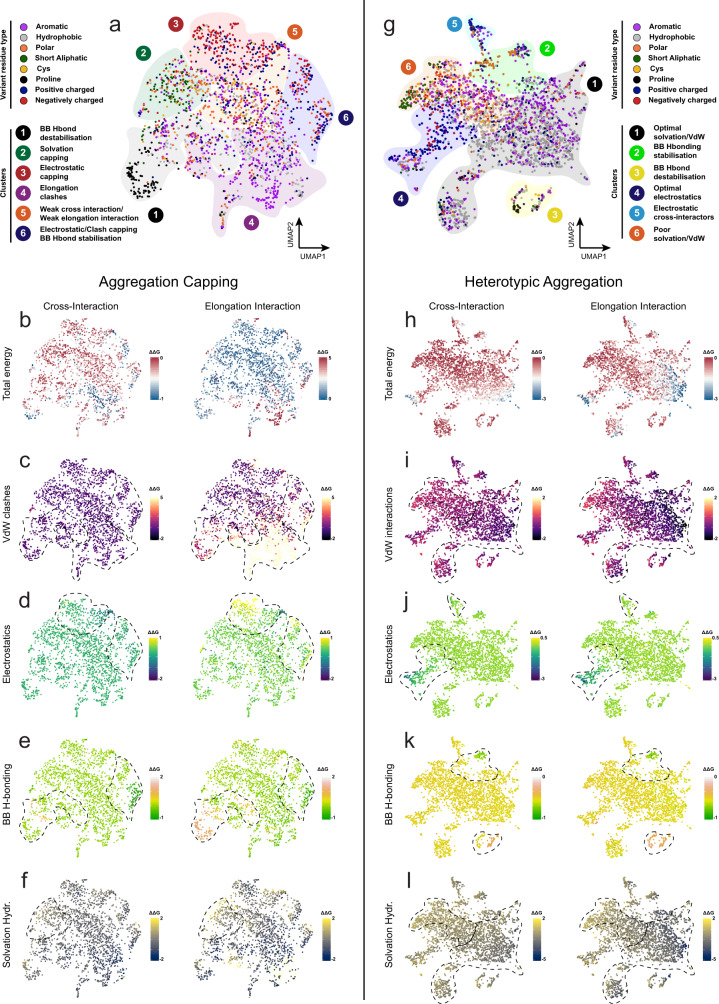
Dimensionality-reduction mapping of the heterotypic sequence space. **a** Clustered modes of interaction that dominate capping variants were identified by analysing **b** total energy and individual energy components, including **c** steric clashes, **d** electrostatics, **e** backbone hydrogen bonding (BB H-bonding) and **f** solvation energy of hydrophobics. **g** Clustered modes of interaction that dominate variants supporting heterotypic aggregation. Independent clusters were identified by analysing **h** total energy and individual energy components, including **i** van der Waals interactions (VdW), **j** electrostatics, **k** backbone hydrogen bonding and **l** solvation energy of hydrophobics. ΔΔG scales are shown in kcal mol^−1^ units. Dimensionality reduction was performed using the Uniform Manifold Approximation and Projection (UMAP) technique.

To objectively define the thermodynamic determinants of cross-amyloid interactions, we performed dimensionality reduction and clustering of the individual energy contributions obtained from FoldX during the mismatch modelling using the Uniform Manifold Approximation and Projection (UMAP) technique (Fig. 3). Three primary clusters of potent cappers were identified (Fig. 3a, clusters 4 and 6 and to a lesser extent cluster 3) to interact well with fibril tips and significantly disrupt further elongation by mapping cross-interaction and elongation interaction energies (Fig. 3b). The first cluster (cluster 4) shows the strongest conversion from stabilising cross-interactions to highly destabilising elongation energies (Fig. 3b). Cluster 4 is primarily occupied by aromatics that efficiently cap fibril ends by introducing significant steric clashes during elongation, but not during initial interaction with the fibril tip (Fig. 3c). Pure electrostatic repulsion (Fig. 3d) can also drive aggregation capping (cluster 3), however is more efficient when coupled with steric hindrance of elongation seen with the longer side chains of the positively charged side chains (cluster 6), but not the negative side chains (cluster 3). Interestingly, globular β-sheet proteins use similar strategies as negative evolutionary invariant designs in their natural folds, in order to prevent uncontrollable edge-to-edge agglomeration^81^, whereas proteins with amyloid-compatible folds, such as β-solenoids, β-rolls and β-ladders are known to be heavily charged, as well as to incorporate polyprolines or aromatic bulges as edge-capping mechanisms to prevent aggregation events at the tip of their folds^82–84^. Another mode of capping refers to disruption of the hydrogen bond network that staples β-strands together in growing amyloids (Fig. 3e). This cluster (cluster 1), is in principle mostly composed of proline variants that act as β-breakers. However, this capping mode is less efficient due to the fact that the levels of disruption are thermodynamically low and similar between cross-interaction and elongation (Fig. 3b). Finally, short side chains can also mildly cap fibril ends (cluster 2) by gradually weakening the free energy gaining from dispersive interactions between the solute and solvent (Fig. 3f), whereas polar and hydrophobic residues are poor cappers that are not particularly driven by specific interactions (cluster 5).

Dimensionality reduction charted a different energy landscape for co-aggregating sequences (Fig. 3g–l). In this analysis, cluster 1 contained the strongest cross-interacting variants (Fig. 3g). Composed principally by hydrophobic (and to a lesser extent, aromatic) side chains, this cluster is defined by tightly packed hydrophobic cores that maximise Van der Waals (Fig. 3i) and solvation (Fig. 3l) contributions and is located at opposite ends of the cross-aggregating sequence space, compared to short and polar side chains (cluster 6). Other cases indicated that electrostatic interactions (Fig. 3j) can also stabilise cross-aggregation (cluster 5), however are more potent when electrostatic contributions are enhanced by successive side-chain stacking during elongation (cluster 4). Finally, backbone hydrogen bonding is a much more limited contributing energy component in co-aggregation (cluster 2) and can still produce heterotypic-compatible variants when slightly destabilising (primarily when introduced by β-breakers, such as Pro residues), due to energetic compensation by other individual energy components (cluster 3) (Fig. 3k).

### Experimental investigation of the modification of self-assembly of aggregation prone regions by APR-like peptides with high sequence similarity

**Fig. 4:**
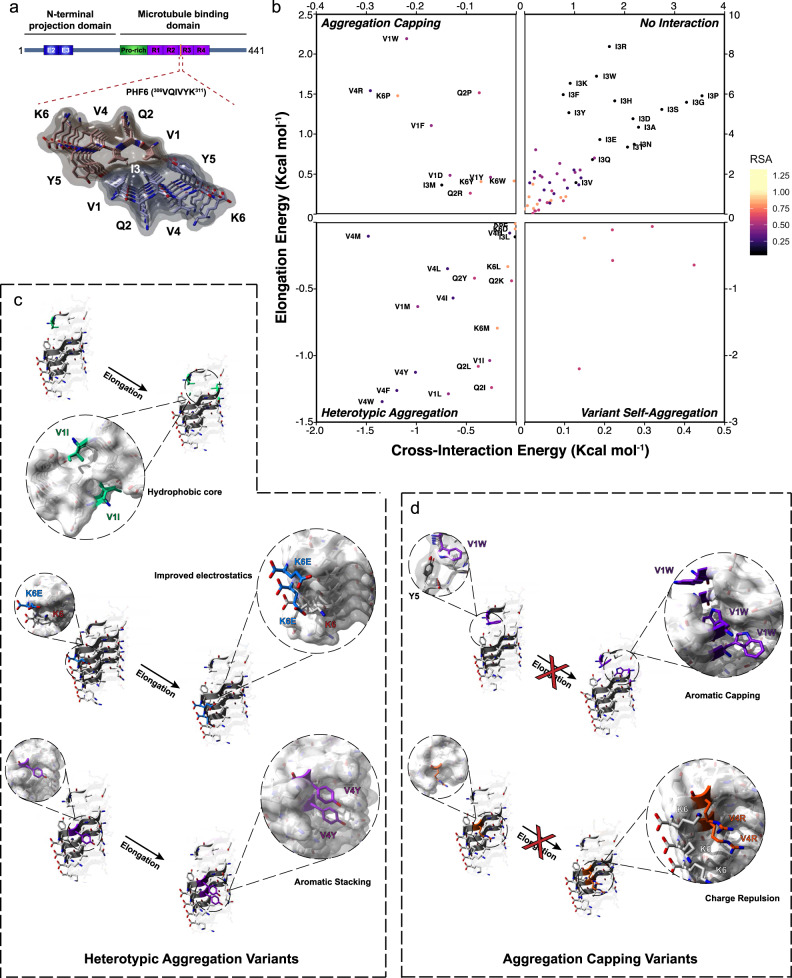
Thermodynamic profiling of cross-interactions for the VQIVYK amyloid core. **a** Schematic representation and positioning of the VQIVYK APR in full-length tau. **b** Quadrant plot analysis of the four modes of interactions for single variants of the VQIVYK APR. **c** Heterotypic aggregation is promoted by hydrophobic mutations that stabilise the aggregation core, electrostatic interactions that improve surface solubility or improved stacking interactions at the exposed fibril surface. **d** Capping interactions are facilitated by the incorporation of bulky aromatic residues that blocked further elongation at the fibril tips through steric clashes or charged side chains that blocked elongation through electrostatic repulsion of stacked charges.

Next, we sought to experimentally investigate these different modes of fibril-tip interactions. For this, we selected a well-known and thoroughly described APR from tau as a case study^85^. The VQIVYK (PHF6) stretch, located in the C-terminal repeat domain of tau, has been demonstrated to be crucial for tau aggregation^86,87^ and is a dominant stabiliser of all tau amyloid polymorphs^40^ (Fig. 4a). Thermodynamic profiling of single variants indicated that cross-interacting variants of the VQIVYK sequence occur primarily at partially buried positions, in contrast to substitutions of the fully buried Ile residue that introduce significant steric clashes during cross-interactions, as well as the Lys side chain that has minimal selectivity due to its high solvent exposure (Fig. 4b). In line with our UMAP clustering analysis, strong co-aggregating variants enabled a tighter packing of the hydrophobic core, utilised electrostatic interactions that promote cross-interactions without causing significant disruptions during elongation or better-defined stacking interactions along the surface of the growing fibril core (Fig. 4c). On the other hand, strong capping variants relied on elongation clashes introduced by aromatic packing or on charge repulsion introduced by successive stacking of charged residues during elongation (Fig. 4d).

**Fig. 5:**
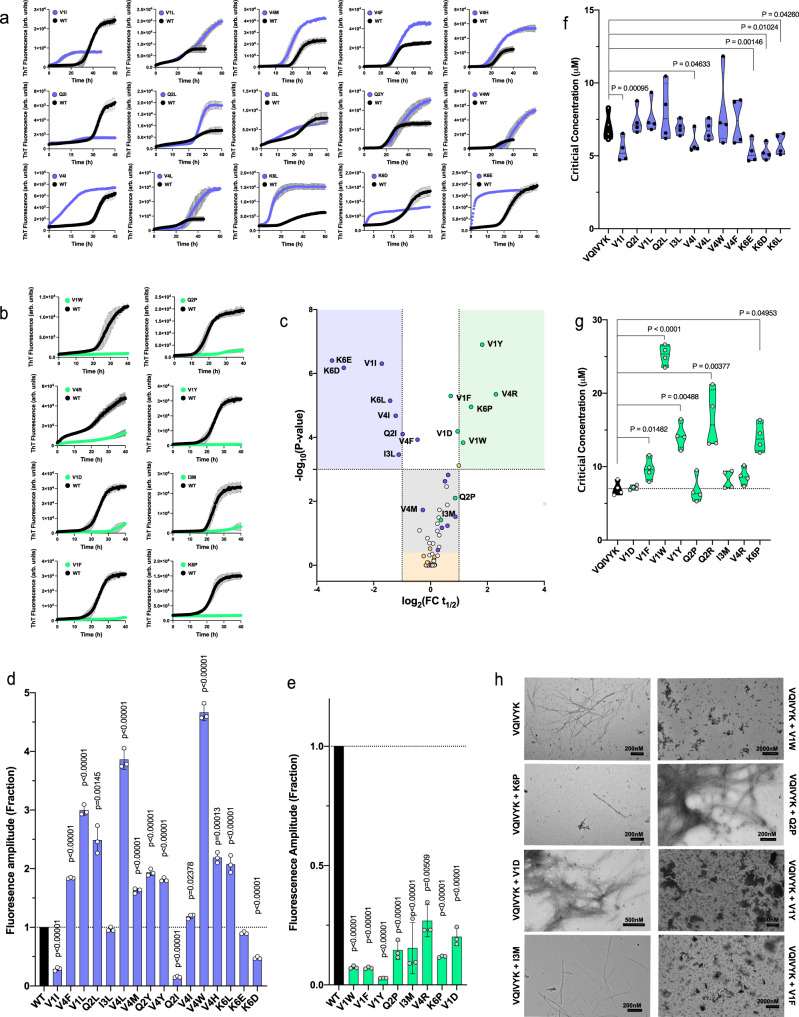
Peptide screening of variant cross-interactions with the VQIVYK aggregation-prone region from tau. Th-T kinetic assays of VQIVYK-alone (125 μM) or in the sub-stoichiometric presence of the strongest **a** co-aggregating or **b** inhibiting single-position variants (25 μM). Curves are shown as means ± SD (*n* = 3 biologically independent samples). **c** Volcano plot analysis of the kinetic halftimes for the entire peptide screen (Fig. S4). Green- and blue-shaded backgrounds indicate capping and co-aggregating sequences of high significance. Sequences with a strong thermodynamic profile for capping or heterotypic interaction are shown in green or purple points, respectively, whereas mutants of the Ile residue are shown in yellow. Statistical significance was determined using one-way ANOVA with Tukey’s test for multiple comparisons (compared to VQIVYK-alone halftime). **d**, **e** End-state fluorescence (*n* = 3 biologically independent samples) and **f**, **g** critical concentration (*n* = 4 biologically independent samples) modifications induced by the strongest **d**–**f** heterotypic aggregating and **e**–**g** capping sequences. Statistical significance was determined using one-way ANOVA with Tukey’s test for multiple comparisons (compared to VQIVYK-alone). **h** Electron micrographs (*n* = 3 independent repeats) of capping mix samples after 7 days of incubation. Minimal to no fibril formation was observed for the strongest cappers (V1W, K6P, V1Y, V1F). Source data are provided as a Source Data file.

To experimentally investigate these calculations, we synthesised a library of 90 peptides corresponding to 78% of all single amino acid substitutions of the VQIVYK APR. We excluded introduction of cysteines to avoid further complexity introduced by the formation of intermolecular disulphides and also avoided substitutions of the Tyr residue at position five, since it enables fast and accurate readout of peptide concentration. First, peptide:APR mixtures were monitored using Th-T aggregation kinetic assays. For this screen, we used sub-stoichiometric mixtures of the variant peptides against PHF6 (1:5 analogy). The reasoning behind this was that it enabled tracing of subtle differences in aggregation kinetics, while at the same time reduced the propensity of most variants to participate in self-assembly. In total, 7 variants, all corresponding to the exposed Lys position were found to still self-aggregate at 25 μM and as a result were excluded from further analysis (Fig. S3). For the rest, following curve fitting of the monitored Th-T curves (Figs. 5a, b and S4), we calculated and summarised fold changes of aggregation half-times (t_1/2_) in a volcano plot, with the negative logarithm of the *p*-values represented on the vertical axis (Fig. 5c). Remarkably, we observed a significant overlap to their thermodynamic profiling, as calculated capping (Fig. 5c, green points) and inducing modifier sequences (Fig. 5c, purple points) overlapped to peptides that had a negative or positive impact on the experimentally determined aggregation kinetics. Additionally, most variants of the buried central Ile position did not engage in cross-interplay, as seen by the minimal changes in aggregation kinetics (Fig. 5c, yellow points). End-state fluorescence analysis validated that most co-aggregating variants increased aggregation (V1I, Q2I, K6E and K6D have reduced Th-T levels, but significantly reduce the kinetic lag-phase) (Fig. 5d), whereas diminished Th-T levels supported the inhibitory effect of the capping sequences (Fig. 5e). It should be noted, however, that changes in fluorescence intensity can also potentially reflect morphological differentiation of the produced fibrils that can affect Th-T binding. Equilibrium thermodynamics analysis using critical concentration determination showed that co-assembly variants, such as V1I, V4I, K6E, K6D and K6L significantly reduced the concentration of the wild-type APR that remains in solution when equilibrium is reached, a clear sign that the free energy of aggregation was impacted (Fig. 5f). Conversely, aromatic, charged and proline substitutions effectively capped and reduced aggregation of VQIVYK, as up to a five-fold increase of the wild-type APR was identified in the soluble fraction of those mixtures, even after a week of incubation (Fig. 5g). The latter was also confirmed using electron microscopy, since almost no amyloid formation was observed for V1W, K6P, V1Y and V1F mixtures at the same timeframe (Fig. 5h).

To further ensure the findings of the UMAP clustering, we also analysed the co-aggregation kinetics of a smaller subset of single variants derived from another experimentally defined APR segment from human Apolipoprotein A-I (ApoA-I)^88,89^. Using the same thermodynamic profiling against a model of the ApoA-I APR, we randomly selected 5 of the strongest hetero-aggregating and capping variant sequences (Fig. S5a–b). Following peptide synthesis, our experimental observations once more supported the heterotypic profiling, since Th-T screening followed by kinetic and end-state analysis (Fig. S5c–e) indicated that all 10 variants had an expected modulatory effect on the aggregation kinetics of the WT sequence.

### Sequence-dependent modifiers alter the morphology of APR amyloid fibrils

**Fig. 6:**
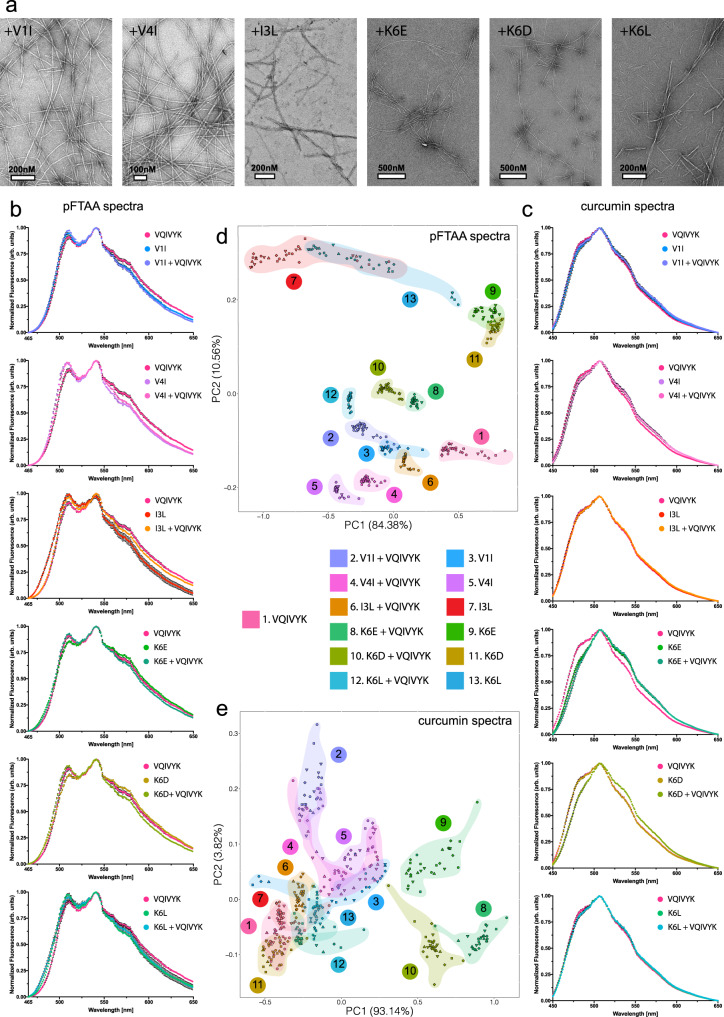
Morphological differentiation induced by co-aggregating sequence-dependent variants of the VQIVYK aggregation prone peptide. **a** Electron micrographs indicate that co-aggregating sequences modify the morphology of amyloid fibrils formed compared to the VQIVYK-alone fibrils. **b**, **c** Normalised binding spectra of **b** pFTAA and **c** curcumin amplified from fibrils derived from mix, peptide modifier-alone or VQIVYK-alone samples. Data points are shown as mean values ± SD. **d**, **e** PCA of the derived **d** pFTAA and **e** curcumin spectra highlighted the distribution of heterogenic conformers that cluster in the defined eigen space. For each sample, six individual preparations were split in five independent aliquots and combined in thirty data points (*n* = 30) per sample in order to represent the intrinsic variability in the fluorescence measurements. Source data are provided as a Source Data file.

Previous studies have indicated that even single mutations can have notable effects on the morphology of amyloid fibrils^74–76,90–96^. Therefore, we employed transmission electron microscopy to investigate if the substoichiometric presence of heterologous APRs, such as described in the previous paragraph, could also alter the morphology of fibrils formed by the VQIVYK APR. Mixtures of conserved variants, such as V1I and V4I, produced longer and thicker fibril networks compared to cognate APR self-assembly. On the other hand, mixtures containing co-interactors incorporating more radical mutations that contain charge inversions, such as K6E or K6D, caused significant morphological differentiation, by forming super-twisted helical fibrils with very tight pitch distances, while the K6L variant produced fibril fragments of shorter lengths (Fig. 6a). To gain further insight on this conformational heterogeneity, we used fluorescence probe binding. Due to its excellent sensitivity, this approach has been used in past studies to determine structural heterogeneity of fibril populations derived even from the same protein constituent^35,97^. Fluorescence spectral acquisitions were obtained side-by-side by adding pFTAA (Fig. 6b) or curcumin (Fig. 6c) to fibrils obtained from peptide mixtures. In each case, the derived spectra were cross-compared to those produced by peptide-only solutions, as well as against solutions containing APR-only amyloid fibrils. Spectral analysis of the pFTAA and curcumin aggregation reporters indicated the presence of different amyloid conformers represented by spectral shifts of band maxima, as well as from inter-band ratio variations. To increase the discriminative sensitivity of the reporters, we coupled this approach to principal component analysis (PCA). Towards this, we normalised the derived spectra after background subtraction and fed the resulting points to PCA. We found that this way structural conformers were actively separated, as the primary principal components (PCs) accounted for more than 90% of the variability in both dye spectra (Fig. 6d, e). The eigen space defined by pFTAA spectral analysis resulted in almost complete separation between different conformers (Fig. 6d). More specifically, the charge switch of the exposed Lys in the case of mixtures containing K6D and K6E resulted in the formation of equally distant (from PHF6 fibrils, cluster 1), yet closely-related diversified amyloid polymorphs. This is evident by the fact that both the peptide-alone (clusters 9, 11) and co-assembly samples (clusters 8,10) gathered close together in the eigen space defined by pFTAA (Fig. 6d), as well as from their observed shared super-twisting morphology. However, the peptide-alone samples do not significantly overlap in the eigen space defined by the curcumin spectra, suggesting that curcumin may have a higher sensitivity in distinguishing subtle morphological differences on the surface of K6D- and K6E-produced fibrils (Fig. 6e). Similarly, the K6L mixture polymorphs (cluster 12) were equally distant to WT amyloid fibrils in terms of their pFTAA binding capacity. Hydrophobic variants also formed distinct conformers that were clearly defined with pFTAA, and to a less extent with curcumin (clusters 2, 4, 6), however their reduced eigen distances to the PHF6 cluster (cluster 1) suggest that these differentiated fibril polymorphs are more similar to the APR fibrils, at least in terms of their dye-binding surface properties.

**Fig. 7:**
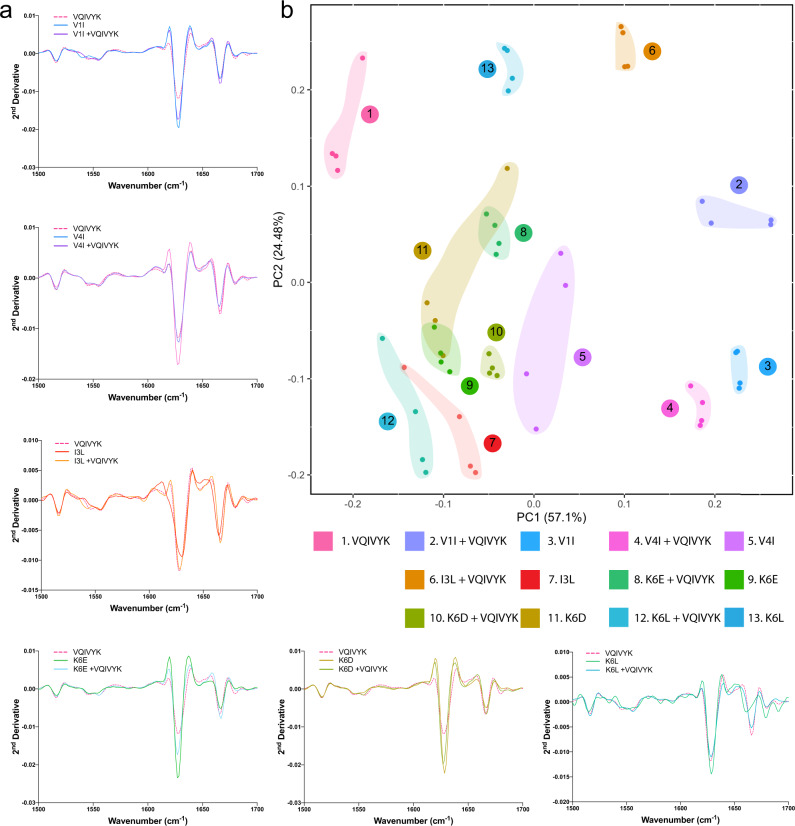
FTIR spectroscopy coupled to PCA revealed the formation of diversified conformers in the presence of VQIVYK co-aggregating modifiers. **a** Second derivatives of the FTIR spectra generated from mixed, peptide modifier-alone or VQIVYK-alone samples, focused around the amide I and amide II region (1700 cm^−1^–1500 cm^−1^). **b** PCA analysis of the derived spectra indicates the presence of different clustering locations in the eigen space representing the formation of differentiated amyloid fibril conformers in the different samples. For each sample, two individual preparations were split in two independent aliquots and combined in four data points (*n* = 4) per sample in order to represent the intrinsic variability in the spectral measurements.

As an additional manner to independently validate the morphological differentiation of the fibrils formed in the mixtures, we employed Fourier-Transform infrared spectroscopy (FTIR) coupled to PCA analysis. This approach supports the identification of structural divergence in amyloid polymorphs by applying vibrational spectroscopy directly on amyloid fibrils and as such, is independent of the physicochemical properties of external probes. Amyloid fibril samples produce strong peaks in the amide I and amide II regions (wavenumber region 1500–1700 cm^−1^), mainly arising from the stretching and bending vibrations of carbonyl- and NH groups,  respectively, that hold together the β-backbones that constitute their axis. As a result, we isolated this spectral region from each sample (Fig. 7a), normalised and fed the resulting points to PCA. Once again, the derived eigen space distributions indicated that mixtures containing variants with modified charge content form strains that cluster in close proximity (clusters 8, 10 and 9, 11) (Fig. 7b). Importantly, however, FTIR spectral analysis highlighted that mixtures containing the more conserved hydrophobic variants also produce fibrils (clusters 2, 4, 6) that are structurally different from WT amyloid fibrils (cluster 1), despite the fact that they closely resemble the dye-binding properties of the latter, suggesting that they may share similar exposed fibril surfaces that facilitate dye binding but ultimately form structurally distinct aggregation cores.

### Optimising the design of structure-based amyloid inhibitors

**Fig. 8:**
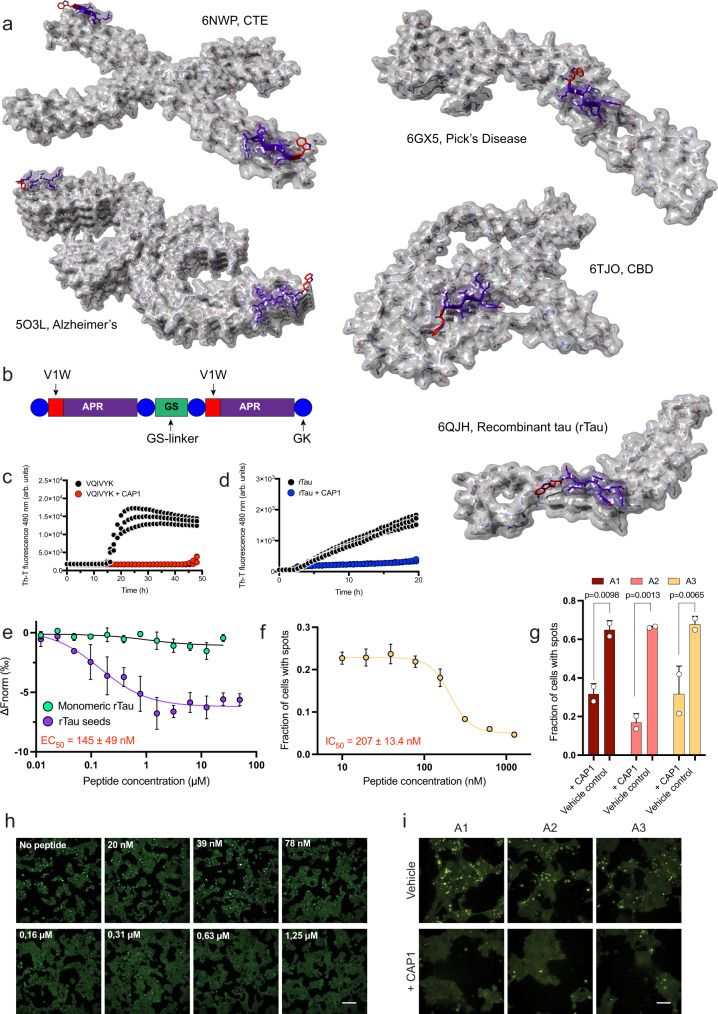
Structure-based inhibition of tau aggregation. **a** Structure-based design of a thermodynamic strong VQIVYK-targeting capper variant sequence (V1W), using full-length tau fibril polymorph cryo-EM structures. Structure representation were prepared using YASARA (v21.8.26). **b** CAP1 tandem peptide design of the V1W capping variant sequence, incorporating Arg gatekeeper residues and a GS-linker. **c**, **d** The CAP1 peptide inhibits both **c** VQIVYK and **d** heparin-induced aggregation of recombinant full-length tau (rTau), as shown by Th-T aggregation kinetics. **e** High affinity binding of CAP1 to tau aggregation seeds (purple curve, 145 ± 49 nM) produced from sonicating heparin-induced tau fibrils (Supplementary Fig. S6). No binding was determined for CAP1 to soluble monomeric tau (green curve), suggesting a high specificity of aggregated species. Curves are shown as mean values ± SD (*n* = 3 biologically independent samples). **f** Dose-dependent inhibition of tau seeding in the FRET biosensor cell line after pre-treatment of rTau (125 nM). An inhibitory concentration value (IC_50_) of 207 nM was determined using curve fitting analysis. Curves are shown as mean values ± SD (*n* = 3 biologically independent samples). **g** Pre-treatment of individual brain extract samples (shown as A1, A2 and A3 (for neuropathological details see Supplementary Table 3) isolated from AD patients (500x dilution) with the CAP1 peptide (500 nM) significantly reduces the fraction of cells containing tau inclusions (three samples, tested in duplicates, *n* = 6). Bar plots are represented as mean values ±SD. Statistical significance was determined using one-way ANOVA with Tukey’s test for multiple comparisons. **h** Representative images of biosensor cells treated with heparin-induced tau seeds pre-incubated with incremental dosage of the CAP1 peptide (Bar = 100 μm). Higher CAP1 concentrations significantly reduce the formation of tau inclusions shown as FRET-intensive puncta (*n* = 3 independent repeats). **i** Representative images of biosensor cells (Bar = 50 μm) treated with AD extracts with and without CAP1 preatreatment (500 nM) (*n* = 3 independent repeats). Source data are provided as a Source Data file.

**Fig. 9:**
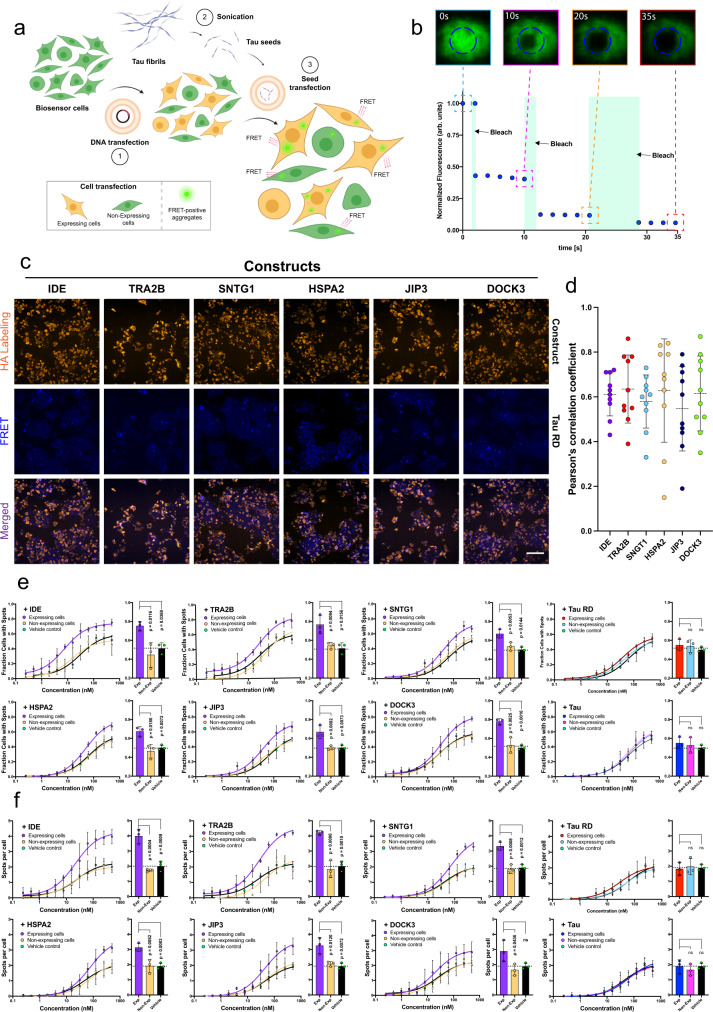
Proteins harbouring localised sequence promiscuity to the VQIVYK aggregation prone peptide modify susceptibility to tau spreading in FRET biosensor cells. **a** Graphical depiction of the experimental setup in the tau biosensor cells. Transient expression of protein constructs, followed-up by secondary tau seeds transfection is measured by quantifying the formation of individual FRET-intensive puncta in construct-expressing (traced with HA staining) and non-expressing cells. Created with BioRender. **b** FRAP measurements of FRET-intensive puncta in the biosensor cells. Complete absence of fluorescence recovery was observed after every successive bleaching step of fluorescence puncta. **c** Representative images of cells expressing individual constructs (HA staining channel) containing tau inclusions shown as fluorescent puncta (FRET channel). Merging of the two channels indicates significant colocalization (purple regions) between HA-intense and FRET-intense regions in expressing cells (Bar = 100 μm). **d** Quantification of HA-intense and FRET colocalisation in expressing cells. Pearson’s correlation coefficient values for individual cells are represented, with error bars indicating mean values ± SD (*n* = 10 from three independent wells). **e**, **f** Absolute quantification of **d** the number of construct-expressing and non-expressing cells containing tau aggregates (*n* = 3 independent experiments) or **e** the number of spots per cell after dose-dependent treatment with tau seeds, compared side-by-side to the vehicle control (no construct transfection), as well as to tau^RD^ and 2N4R transfected cells, respectively. Bar plots highlight differentials observed in cells when treated with the highest concentration of tau seeds (500 nM). Data are represented as mean values ± SD. Statistical significance was calculated using one-way ANOVA with multiple comparisons. Source data are provided as a Source Data file.

Recent developments have pointed out that sequence-driven structured-based inhibition of amyloids may yield an effective approach to counter amyloid formation^55,87,98–106^. In agreement, our thermodynamic profiling and umap reduction analysis also revealed that certain modes of interaction are more successful in capping the ends of growing aggregates, highlighting that aromatic variants have the strongest potential by introducing steric hindrance during elongation. We validated this notion in vitro by showing that strong cappers of the VQIVYK APR also often incorporated aromatic residues, while the V1W capper specifically also reduced amyloid formation and critical concentration after several days of co-incubation. Coupling this approach to the recent burst of cryo-EM structures of different tau strains^34^, the V1W capper is also expected to be efficient against in vitro prepared amyloid fibrils from recombinant tau (rTau) as well as to brain-extracted tau strains, due to the central position of the PHF6 segment in their amyloid core (Fig. 8a). Previous studies have also proposed that modified scaffolds designed to maximise interaction (e.g. tandem or microcyclic designs) and impose structural constraints can enhance the activity of structure-based inhibitors^105,107^, a notion that was also validated during our previous work on antiviral, antibacterial and cancer cell-targeting aggregation-prone peptide designs^78,79,108^. Following this premise, we designed a tandem peptide (named CAP1) incorporating the V1W capping sequence and experimentally tested its capacity to inhibit tau aggregate formation (Fig. 8b). In vitro Th-T kinetics validated the potency of the CAP1 capping activity, as it successfully inhibited the self-assembly of both the PHF6 hexapeptide (Fig. 8c) and rTau (Fig. 9d). To investigate the targeting specificity of CAP1 towards aggregate species of tau, we utilised microscale thermophoresis (MST). Towards this end, we generated fluorescently labelled rTau seeds by sonicating end-state amyloid fibrils formed after co-incubation of ATTO_633_-labelled and unlabelled rTau (1:9 analogy). Dose-response affinity analysis disclosed that CAP1 specifically binds to rTau seed aggregates with high affinity (EC_50_ = 145 ± 49 nM), whereas no significant binding was observed against monomeric rTau, respectively (Fig. 8e). Similar to this, seeding inhibition was also calculated in the biosensor cell line by counting the formation of FRET-positive spots as a function of CAP1 concentration. The derived dose-response curve revealed a high inhibitory effect for CAP1 with an impressive IC_50_ of about 200 nM (Fig. 8f, h), that is very similar to the determined binding affinity of the peptide and corresponds to a fivefold or higher improvement in efficacy compared to optimal tau inhibitors from previous studies^87^. More importantly, we also tested the CAP1 capping activity against tau brain extracts isolated from three individual AD patients (each tested in duplicates). Impressively, upon pre-treatment of the isolated physiological tau conformers with the CAP1 design (500 nM), seed propagation was significantly reduced in the same cell line (Fig. 8g, i). Overall, our results highlight that due to the current surge in amyloid template structures^39^, our growing structural knowledge of amyloids constitutes thermodynamic profiling, coupled to optimised scaffold design, a competent strategy to design aggregation suppressors of high specificity.

### Over-expression of full-length proteins harbouring APR variants modulates aggregation in cells

Our in vitro screening showed that short sequence stretches with homology to APRs are potential aggregation modifiers, supporting accumulating data on sequence-driven amyloid cross-interactions^19,51–54^. To extend this notion further, we sought to assess whether full-length proteins harbouring such homologous hotspots are vulnerable to cross-aggregation. Based on a proteome-wide search for PHF6 sequence homologues and subsequent manual curation, we selected and tested a subset of 11 full length and/or domain regions of proteins containing such co-aggregation hotspots, in addition to full-length (tau^2N4R^) and repeat-domain tau (Tau^RD^) which were included as controls (Supplementary Table 2). In order to experimentally investigate if these protein regions can indeed participate in cross-talk and modulate tau aggregation, we designed constructs for transient expression (Fig. 9a). To distinguish expressing from non-expressing cells, each gene construct included a fluorescent reporter (mKO2), separated by an internal ribosome entry site (IRES). The constructs were transfected into HEK293 Tau^RD^-P301S-CFP/YFP expressing biosensor cells that are highly sensitive reporters of tau-specific seeding-competent aggregates^109^. Recombinant full-length tau aggregation was monitored in vitro (Fig. S6a) and used to produce uniform tau seeds by sonicating end-state fibrils (Fig. S6b) that were then concentration-dependently transfected into biosensor cells (Fig. 9a). This yields a concentration-related gradient induction of aggregation of the cellular tau reporter construct that can be quantified through image analysis by counting the formation of FRET-positive puncta. Using this experimental setup, we compared the seeding capacity of exogenously added tau aggregates in cells expressing our constructs compared to controls, and we verified the aggregated nature of the resulting cellular inclusions, using fluorescence recovery after photobleaching (FRAP) (Figs. 9b and S7). Moreover, construct colocalization with the tau inclusions was traced using immunofluorescence (HA staining) (Fig. 9c). High-content screening revealed that six of the selected constructs colocalize with tau FRET-positive inclusions (Fig. 9c, d). Cells that strongly expressed these constructs were significantly more susceptible to seeding of tau aggregation, as induced tau aggregation raised by at least 20% in transfection-positive cells, when compared to both non-expressing, as well as to non-transfected cells (vehicle control) and even increased to 30% for specific constructs at high seeding concentrations (IDE, TRA2B and DOCK3) (Fig. 9e). Impressively, concentration-dependent quantification analysis revealed that this effect remained even when treating with lower concentrations of seeds, with certain proteins (Fig. 9e, IDE and TRA2B) rendering cells vulnerable to tau seeding even at picomolar concentrations, whereas no visible aggregation was observed at similar conditions in the corresponding controls. On the other hand, over-expression of tau^RD^ and tau^2N4R^, using the same construct design, did not have any effect on the efficiency of tau aggregation and spreading in the cells, which was also recapitulated for the rest of the constructs included in the selected subset (Fig. S8a–e).

Another indication that the transient over-expression of these constructs increases cellular susceptibility to tau aggregation was highlighted by the morphological analysis of cellular inclusions (Fig. 9f). Results showed that with the exception of DOCK3, there was a concentration-dependent increase in the number of inclusions formed per cell, with certain constructs having a doubling or higher effect (IDE, TRA2B and SNTG1) when exposed at higher concentrations of tau seeds, compared to the controls. Similarly, no significant morphological differentiation was observed when transfecting with either tau^2N4R^, tau^RD^ or any of negative constructs (Fig. S8f–j), respectively, thus further supporting the notion that apart of simply participating in co-aggregation with cellular tau inclusions, these proteins may also actively enhance cellular susceptibility to tau aggregation.

## Discussion

Recent developments in structure determination methodologies, such as cryo-EM, microcrystal electron diffraction and solid-state NMR have provided significant advancement in the field of amyloids. Our structural insight on different architectures of amyloid polymorphs, APR aggregation cores and even oligomeric species is now reaching levels that support a broader understanding of the key structural features that mediate major amyloid-related properties, such as their self-assembly mechanisms, kinetics and overall structural stability^39–50^. On the other hand, our knowledge on amyloid cross-talk with other protein components still remains limited. Despite this, more and more evidence is coming to light indicating that cross-aggregation could be on the basis of defining the apparent selective vulnerability of specific cell types to aggregates or complex spatiotemporal spreading patterns of amyloid deposition, while may also explain observed overlaps between distinct pathologies or why certain amyloid conformers are associated to them, respectively^19^. Building on the above, we provided here a deeper understanding of the structural determinants that define sequence dependency of amyloid cross-aggregation interactions. To achieve this, we performed a systematic thermodynamic evaluation, coupled with multidimensionality analysis, to identify the dominant forces that mediate cross-talk with experimentally determined APR amyloid cores. Our results indicated that even for highly conserved sequences, such as single-position variants, a thermodynamically favourable fit within the defined aggregation core is rather hardly accommodated. This notion comes to add to our recent thermodynamic profiling of the fibril cores of full-length amyloid fibrils, which highlighted that these APR segments provide an extremely conserved framework that commonly stabilises different polymorphs. Furthermore, the same analysis revealed that although additional segments of the polypeptide chain participate in hetero-packing when incorporated in the fibril core, these segments are described by energetically degenerative tertiary packing^40^, thus supporting our findings on the limitations of cross-aggregation interactions within amyloid cores.

Owing to the above, we next tested whether proteins containing homologous sequence stretches as potential co-aggregation hotspots could be particularly susceptible to the aggregation propensity of amyloidogenic proteins. Our cellular screening assay, using tau as a case study, validated this premise, yet importantly also indicated that these proteins can further influence the seeding efficiency, morphology and spreading of tau aggregates in the cells. These results suggest that sequence-specific modulatory effects can work in parallel to other mechanisms, as for instance supersaturated sub-proteomes^28–32^ or heterotypic-induced biomolecular condensation^110–113^, to influence amyloid interplay with the background proteomic content of various cell types, thus promoting selective cellular vulnerability. This becomes more evident when considering the role of the six proteins that were here found to significantly modify tau spreading in cells, as they have major impact in progression of AD and various other neurodegenerative disorders. In more detail, the insulin degrading enzyme (IDE) is imperative during clearance of Aβ peptide fragments and has been recently designated as a prime target for therapeutic treatments against both AD and T2D, respectively^114–116^. Our results showed that IDE colocalises in tau inclusions and promotes spreading, processes that precede Aβ accumulation and plaque formation, suggesting that the latter can be amplified by its early entrapment and gradual loss of function in tau aggregates. Similarly, the dedicator of cytokinesis, DOCK3 (also known as modifier of cell adhesion - MOCA and presenilin-binding protein - PBP), is another important protein involved AD progression and several other neurological deficiencies, including tauopathies and Creutzfeldt-Jakob disease. This enzyme is a known interactor of presenilin, a genetic marker involved in AD, and has also been shown to redistribute and accumulate in neurofibrillary tangles extracted from AD brain samples^117^, indicating that it also colocalises in vivo with tau aggregates. Transformer-2 homolog beta (TRA2B) is a splicing factor that controls alternative splicing of the *MAPT* gene encoding expression of tau. The reportedly altered expression and activity of TRA2B has been directly implicated to major neurological disorders, such as AD and PD, as well as to promoting tau hyperphosphorylation^118–120^. Importantly, this comes to add to recent evidence indicating that several nuclear speckle components, such as TRA2B, mislocalize to cytosolic tau aggregates in cells, mouse brains, and brains of individuals with AD, frontotemporal dementia (FTD), and corticobasal degeneration (CBD)^121^. Synaptotagmin-1 (SNTG1) is essential for proper synaptic transmission and cognitive function. Recent mass spectrometry assays on cerebrospinal fluid extracted form AD patients, highlighted its use as a biomarker in dementia^122^. Furthermore, SNTG1 has a compensatory protective function by gradually increasing its binding to presenilin in the aging brain, an association that has been shown to deter in sporadic AD brains^123,124^. The above suggest that gradual depletion of SNTG1 due to co-aggregation with tau can have detrimental cascading effects during AD progression. The *MAPK8IP3* gene encodes for JIP3, a neuronally enriched critical regulator of axonal lysosome abundance^125^. Loss of JIP3 functionality in pluripotent stem cells (iPSCs) results in the aberrant accumulation of Aβ42^126^, suggesting that its inactivation by co-aggregation with tau at the early stages of AD brains can be an important initiator for Aβ proliferation. Finally, despite the known activity of Hsp70 in preventing or inhibiting tau aggregation, our assay revealed that a fragment containing both the nucleotide and substrate-binding domain of the molecular chaperone is also vulnerable to tau co-assembly, suggesting that as the proteostatic control mechanisms of cells erode over ageing, protective components such as chaperons may worsen the load produced by amyloids. At this point, our work here presents evidence on a generic structural mechanism that cultivates sequence-driven interactions of amyloids to various cellular protein components. Future work is required in order to contextualise this structural mechanism to other generic modes that promote heterotypic aggregation or to understand how and if sequence-specific heterotypic knock-down of certain proteins is amenable to spatiotemporal spreading patterns and selective cellular toxicity of neurodegenerative diseases.

Structured-based designs have been used for years as a strategy for the development of molecular inhibitors in conformational diseases^55,87,98–106^. Following this logic, we also showed here that the accumulating numbers of amyloid structures, combined to detailed thermodynamic profiling of sequence-specific heterotypic interactions can be used to optimise the design of aggregation cappers. By applying this approach on cryo-EM structures of tau polymorphs, we tested the efficacy of a tandem peptide design, CAP1, in blocking tau aggregation in vitro and in cells. Our results indicated that CAP1 selectively binds with high affinity to tau aggregates formed in vitro in the presence of heparin and blocks its cellular spreading with a five-fold improved efficacy compared to previous designs. At the same time, CAP1 was efficient in inhibiting the seeding capacity of tau polymorphs isolated from AD patient brain samples, thus indicating the strength of this comprehensive approach in significantly improving the selective affinity of inhibitor designs that can target multiple amyloid polymorphs and suggesting that this is a promising methodology for the development of therapeutics in amyloidosis diseases.

## Methods

### Thermodynamic profiling using the FoldX energy force field

We collected a complete set of recently published APR amyloid core structures from the PDB^127^ (Supplementary Table 1). First, we utilised the FoldX energy force field^77^ to generate cross-interaction and elongation instances of cross-assembly for every template by mutating single residues of chains located at its fibril ends (Fig. 1a–c). Second, we used FoldX to perform a thermodynamic breakdown of the energy potentials for both modes of interaction. FoldX as a method has been described in length previously^77^, but briefly here, during free energy calculations, the force field first calculates the free energy contribution of each atom in protein interfaces based on its own position relative to neighbours in the complex. Following this, FoldX subsequently sums individual contributions together, first at the residue level, to calculate segment interaction potentials. This allows to accurately chart the free energy contribution (Δ*G*) of each residue participating in intermolecular interfaces but also reports on individual thermodynamic components (e.g. Van der Waals, electrostatics, H-bonding or electrostatics, entropy) contributing to overall structural stability. Based on this premise, interaction energies per variant were represented as differentials cross-compared to the free energy potential of the wild-type interaction:

1 $${\Delta \Delta G}_{{{{{{{\mathrm{cross}}}}}}}-{{{{{{\mathrm{interaction}}}}}}}}=\,{\Delta G}_{{{{{{{\mathrm{edge}}}}}}}\;{{{{{{\mathrm{variant}}}}}}}}-\,{\Delta G}_{{{{{{{\mathrm{edge}}}}}}}\;{{{{{{\mathrm{APR}}}}}}}}$$

2 $${\Delta \Delta G}_{{{{{{{\mathrm{elongation}}}}}}}}=\,{\Delta G}_{{{{{{{\mathrm{elongation}}}}}}}\;{{{{{{\mathrm{variant}}}}}}}}-\,{\Delta G}_{{{{{{{\mathrm{edge}}}}}}}\;{{{{{{\mathrm{APR}}}}}}}}$$

where ${{\Delta }G}_{{{{{{{\mathrm{edge}}}}}}\; {{{{{\mathrm{variant}}}}}}}}$ is the free energy of the cross-interaction of a variant chain at the APR fibril end (Fig. 1b), ${{\Delta }G}_{{{{{{{\mathrm{elongation}}}}}}\; {{{{{\mathrm{variant}}}}}}}}$ is the free energy of interaction between a single-variant chain docked against an APR axial end occupied by variant chains (Fig. 1c) and ${{\Delta }G}_{{{{{{{\mathrm{edge}}}}}}\; {{{{{\mathrm{APR}}}}}}}}$ corresponds to the interaction energy between the cognate APR chain against its own amyloid core (Fig. 1a). The reasoning behind using differential ΔΔG values is two-fold: (i) the calculated differentials are comparisons to thermodynamically stable interacting chains derived from experimentally determined APR crystal structures, (ii) while as differentials, they enable global analysis since they only report on the effects in free energy imposed by single mutations and are indifferent to the relevant starting stability of the template structure.

#### Determination of peptide propensities

Relative solvent accessibility values were calculated for each residue position of the template structures using the maximum allowed solvent accessibility scale by Tien and co-workers^128^. The effects of positional mutations in the overall solubility and β-propensity of APRs were defined as differentials of partition coefficients, calculated using PlogP^129^, and Chou and Fasman^130^ propensities between WT and variant, respectively.

### Uniform manifold approximation and projection dimensionality reduction

A defined sequence space was constructed by merging the identified single-variant capping and cross-aggregating sequences for the complete set of 83 experimentally APR amyloid core structures from 18 proteins (Supplementary Table 1). A 30-dimensional vector, composed of a wide list of individual energy components, including H-bonding, electrostatics, entropy, solvation and Van der Waals interactions between both backbone and side-chain atoms, among others, was extracted using the FoldX force field. First, this multidimensional vector was analysed using principal component analysis and the derived principal components were subsequently fed into a umap matrix. Finally, each data point, representing a single-position variant, was reduced and embedded in 2D-space using the R umap package, with the minimum distance to the nearest neighbour set to 0.3 and the number of neighbours to 15, in order to avoid extreme local clustering complexity.

### Peptide library synthesis

Peptides were synthesised using an Intavis Multipep RSi solid phase peptide synthesis robot. Peptide purity (>90%) was evaluated using RP-HPLC purification protocols and peptides were stored as ether precipitates (−20 °C). Peptide samples were initially pre-treated with 1,1,1,3,3,3-hexafluoro-isopropanol (HFIP) (Merck), then dissolved in traces of dimethyl sulfoxide (DMSO) (Merck) (<5%) and filtered through 0.2 μm filters before dissolving in the final buffer.

### Aggregation assays

For Th-T kinetics, each peptide variant was pre-treated to form films. The cognate APR peptide was then dissolved and filtered in DMSO, then split into equal aliquots that were used to dissolve the variant films. The resulting mixtures were subsequently dissolved in PBS. Final concentration of the WT APR was set to 125 μM and 25 μM for the variants (1:5 analogy). Thioflavin-T (Sigma) was added in half-area black 96-well microplates (Corning, USA) at a final concentration of 25 μM. Fluorescence was measured in replicates (*n* = 3) using a PolarStar Optima and a FluoStar Omega plate reader (BMG Labtech, Germany) at 30 °C, equipped with an excitation filter at 440 nm and emission filter at 490 nm and using the Omega (v5.11 and MARS software (v3.32). To determine kinetic rates, derived spectra were normalised and fitted following:

3 $$Y={y}_{0}+\left(\frac{({y}_{{\max }}-{y}_{0})}{\left(\right.1+{\exp}(-(x-{t}_{1/2})* k )}\right)$$

where fluorescence intensity (Y) is represented as a function of time (*x*). *y*_max_ and *y*_0_ indicate maximum and starting fluorescence values, respectively, whereas *t*_1/2_ and *k* are the kinetic half times and elongation rates of the fitted curves. *t*_1/2_ were determined separately for each individual replicate per sample. For endpoint solubility analysis, following incubation for 7 days, peptide mixture preparations were subjected to ultracentrifugation at 76.000 g for 1 h at 4 °C. The isolated supernatant was mixed with 6 M Guanidine-HCl and 0.2% acetic acid and injected into an analytical HPLC. Peptide concentration was then calculating by integrating the AUC values of the peak corresponding to the WT APR peptide. All data were plotted using Prism 9.

### Transmission electron microscopy

Peptide mixtures were incubated for 7 days at room temperature in order to form mature amyloid-like fibrils. Suspensions (5 μL) of each peptide solution were added on 400-mesh carbon-coated copper grids (Agar Scientific Ltd., England), following a glow-discharging step of 30 s to improve sample adsorption. Grids were washed with milli-Q water and negatively stained using uranyl acetate (2% w/v in milli-Q water). Grids were examined with a JEM-1400 120 kV transmission electron microscope (JEOL, Japan), operated at 80 keV.

### Fluorescence dye binding

For fluorescence dye binding, we prepared equimolar mixtures, variant-only and PHF6 APR-only preparations at a concentration of 500 μM in milli-Q water. For statistical analysis, six individual preparations were each split into five aliquots, resulting in thirty in total replicates per sample (*n* = 30) that were left at ambient conditions for seven days to form amyloid fibrils. Suspensions (20 μL) of peptide solutions were then mixed with pFTAA and curcumin at 0.5 μM and 5 μM final concentration, respectively. Fluorescence emission spectra were recorder in low volume 384-well black plates with clear bottom (Corning) for pFTAA (465 nm–600 nm) and curcumin (450 nm–650 nm), after exciting at 440 nm and 420 nm, respectively, using a ClarioStar plate reader at 30 °C (BMG Labtech, Germany). The acquired spectra were background subtracted, normalised and plotted using Prism 9. The derived normalised spectra were then subjected to principal component analysis using the prcomp function in R.

### Fourier-Transform infrared spectroscopy

Similar equimolar mixtures, variant-only and PHF6 APR-only preparations were used for FTIR measurements. Each sample was split into equal aliquots and allowed to incubate for 7 days at ambient conditions. Droplets (5 μL) of peptide samples (*n* = 4) were cast onto a 96-well silicon microplate (Bruker) and dried to form thin films. FTIR spectra were recorded as averages of 64 spectral scans at 4 nm^−1^ resolution in transmission mode to improve signal-to-noise ratio, using an HTS-XT FTIR microplate reader (Bruker). Background correction was performed by subtracting spectra obtained from a blank position of the microplate. Spectral normalisation and 2^nd^ derivatives with a 13-point smoothing, using Savitzky-Golay filtering^131^, were calculated using the OPUS software (v8.5.29) after isolation of the amide I and amide II regions of the derived spectra (1700–1500 cm^−1^). The normalised spectra were subjected to principal component analysis using the prcomp function in R.

### Fluorescence recovery after photobleaching

Confocal microscopy was used to acquire images for fluorescence recovery. Instances were acquired as individual frames on a Nikon A1R Eclipse Ti confocal microscope, equipped with a Plan APO VC ×60 oil lens. For bleaching, we defined a region-of-interest (ROI) that was excited using the CFP-donor laser line (405 nm) at 100% laser power and emission was collected using the YFP acceptor filter (550 nm). FRAP was performed in pulses of successive time increments (0.06 s, 0.6 s and 1.2 s). Between pulses, total fluorescence of the ROI was measured for 10 s (ROI within spot) or 20 s (ROI in the cellular background) by acquiring single frames every 2 s (5 or 10 frames per window, respectively). Total fluorescence of the ROI in each frame was normalised to the total fluorescence of the same pre-bleached region (*t* = 0) to check for potential recovery.

### Preparation of recombinant Tau (rTau) fibrils and seeds

Recombinant full-length tau (tau^2N4R^) was produced following previous established protocols^132^. Lyophilised protein aliquots were freshly dissolved in 10 mM HEPES pH 7.4 supplemented with 100 mM NaCl at a final concentration of 10 μM. After filtration, using 0.2  μM PVDF filters, the protein solution was spiked with 5 μ μM of heparin (Sigma) and aggregation was monitored by adding 25 μM of Th-T in half-area black 96-well microplates (Corning, USA). Fluorescence was measured in triplicates, using a FluoStar Omega plate reader (BMG Labtech, Germany) at 37 °C, equipped with an excitation filter at 440 nm and emission filter at 490 nm. To generate seeds, endpoint amyloid fibrils were sonicated for 15 min (30 s on, 30 s off) at 10 °C, using a Bioruptor Pico sonication device (Diagenode).

### Extraction of tau filaments

Ethical approval to access and work on the human tissue samples was given by the UZ Leuven ethical committee (Leuven/Belgium; File-No. S63759). An informed consent for autopsy and scientific use of autopsy tissue with clinical information was granted from all subjects involved. Following approval, brain tissue from autopsy cases was received from UZ/KU Leuven Biobank. Sarkosyl-insoluble material was extracted from cortex tissue of three individual patients with Alzheimer’s disease (designated here as A1–A3, Supplementary Table 3), as shown previously^133^. Briefly, the tissue was homogenised with a FastPrep (MP Biomedicals) in 10 volumes (w/v) cold buffer consisting of 10 mM Tris-HCl pH 7.4, 0.8 M NaCl, 1 mM EGTA and 10% sucrose, followed by centrifugation at 20,000 × *g* for 20 min at 4 °C. Universal Nuclease (Pierce) was added to the supernatant, followed by a 30 min incubation at room temperature. The sample was then brought to 1% Sarkosyl (Sigma) and incubated for 1 h at room temperature while shaking (400 rpm), followed by centrifugation at 350,000 × *g* for 1 h at 4 °C. The pellet was washed once, resuspended in 50 mM Tris-HCl pH 7.4 (175 mg of starting material per 100 µl) and stored at −80 °C.

### FRET cellular transfection assays

HEK293 Tau^RD^-P301S-CFP/YFP expressing biosensor cells^109^ were purchased from ATCC and cultured in DMEM medium, supplemented with 10% FBS at 37 °C, and a 5% CO_2_ atmosphere. Gene constructs (Supplementary Methods) were generated and onboarded to a pTwist CMV expression vector by coupling double-tagged (N-terminal HA and C-terminal FLAG recognition sites) genes of interest to an mKO_2_ fluorescence reporter, separated by an IRES site to enable independent co-expression (Twist Biosciences). Due to restrictions imposed by the construct synthesis, for proteins longer than 500 residues we designed shorter domain-constructs containing the homologous sequences (Supplementary Table 2). Biosensor cells were plated in poly-l-lysine coated 96-well plates (PerkinElmer) at a density of 20000 cells/well. DNA transfection (100 ng) and tau seeds transfection was performed 6 h and 48 h later, respectively, using Lipofectamine 3000 according to the manufacturer guidelines. For seed transfections specifically, a volume of 4.8 μL of the seed sample, mixed with 0.2 μL of Reagent 3000, was added to a mixture of 0.3 μL of Lipofectamine 3000 with 4.7 μL of Opti-MEM medium. Cells were fixed with 4% formaldehyde 24 h after seeding. Fixed cells were stained with DAPI (Thermofisher, D1306) following the manufacturer protocol. For immunofluorescence staining, primary antibody staining at 1:1000 dilution was performed in 1% BSA with an HA-tag (C29F4) Rabbit mAb (Cell Signalling, #3724), followed by secondary staining with an Alexa Fluor 647 goat anti-rabbit antibody (ThermoFisher, A-21245) at 1:1000 dilution in 1% BSA for 1 h. Three individual plate preparations were performed for each construct gradient as independent experiments (*n* = 3). High-content screening was performed at the VIB Screening Core/C-BIOS, using an Opera Phenix HCS (PerkinElmer) equipped with proper filter channels to track tau aggregation through FRET (Ex:405, Em:550), construct colocalization through HA staining (Ex:647, Em:667) and DAPI staining (Ex:405 Em:430). Image storage and segmentation analysis was performed using the Columbus Plus digital platform (PerkinElmer). Quantification of colocalization was performed using Coloc2 in ImageJ.

### Microscale thermophoresis

MST measurements were performed to calculate binding affinities. Monomeric tau was labelled using amine reactive ATTO_633_ (ATTO_633_-NHS), following the manufacturer guidelines. Labelled tau aggregates were prepared using a 1:9 analogy of labelled to unlabelled monomeric tau, following the same aggregation protocol described for the unlabelled protein. 25 nM of ATTO_633_-monomeric tau or ATTO_633_-tau seeds were mixed against the CAP1 inhibitor, which was dissolved and titrated down starting from 50 μM, in tau buffer (HEPES 10 mM, 100 mM NaCl). Measurements were recorded on a Monolith NT automated instrument (NanoTemper Technologies GmbH, Germany) with a red-laser channel at 5% LED excitation power and medium MST power at ambient conditions. Affinity constants and experimental data fitting was performed using the NanoTemper analysis software (v2.2.4) and results were depicted as differentials between the bound and unbound state after baseline subtraction (ΔFnorm) over inhibitor concentration in the logarithmic scale.

### Structure-based inhibition using FRET tau biosensor cells

We co-incubated tau seeds, at a concentration of 125 nM, produced from recombinant full-length tau^2N4R^ as described above, with a titrated concentration gradient of the CAP1 peptide for 2 h at room temperature. HEK293 Tau^RD^-P301S-CFP/YFP expressing biosensor cells were plated in poly-L-lysine coated 96-well plates (PerkinElmer) at a density of 20000 cells/well and subsequently transfected with pre-incubated mixtures of rTau seeds/CAP1, using Lipofectamine 3000. Specifically, a volume of 4.8 μL of the seed/CAP1 pre-incubated sample, mixed with 0.2 μL of Reagent 3000, was added to a mixture of 0.3 μL of Lipofectamine 3000 with 4.7 μL of Opti-MEM medium. Cell fixation was performed 24 h after transfection using 4% formaldehyde and cellular imaging was performed using an Operetta CLS (PerkinElmer). Three individual plate preparations were used as independent experiments for statistical significance (*n* = 3). Data storage and analysis was performed using the Columbus Plus digital platform (PerkinElmer). For the seeding assays with the brain extracts, the stored extracts were first diluted in 10 mM HEPES pH 7.4 supplemented with 100 mM NaCl (500x) and then co-incubated for 10 min with the CAP1 peptide. The biosensor cells were then plated in poly-L-lysine coated 96-well plates (PerkinElmer) at a density of 5000 cells per well and allowed to attach for 24 h. Subsequent transfection, fixation and measurements were performed as described for the recombinant seeds.

### Reporting summary

Further information on research design is available in the Nature Research Reporting Summary linked to this article.

## Supplementary information

Supplementary Information

Reporting Summary

## Data Availability

The data supporting the findings of this study are available from the corresponding authors upon reasonable request. The source data underlying Figs. 2, 5, 6b, c, 8c, d, e and 9b, d, e and Supplementary Figs. 3, 4, 5c, d, 6a and 8 are provided in a Source Data file and in Supplementary Tables 1–3. The dataset of APR core structures is provided in Supplementary Table 1. The protein constructs used in the cellular assays are shown in Supplementary Table 2. All clinical cases are listed in Supplementary Table 3 with the main parameters, with the main clinical diagnosis, age and gender. There are no reuse restrictions for the published data. Due to legislation and privacy protection any medical reports and files of the cases included in this study cannot be made available. Response time for additional requests is 2 months. Source data are provided with this paper.

## Source data

Source Data

## References

[1] Van Dam D, Vermeiren Y, Dekker AD, Naude PJ, Deyn PP (2016). Neuropsychiatric disturbances in Alzheimer’s Disease: what have we learned from neuropathological studies?. Curr. Alzheimer Res..

[2] Dugger, B. N. & Dickson, D. W. Pathology of neurodegenerative diseases. *Cold Spring Harb. Perspect. Biol.*10.1101/cshperspect.a028035 (2017). 10.1101/cshperspect.a028035 PMC5495060 28062563

[3] Eisenberg D, Jucker M (2012). The amyloid state of proteins in human diseases. Cell.

[4] Chiti F, Dobson CM (2017). Protein misfolding, amyloid formation, and human disease: a summary of progress over the last decade. Annu. Rev. Biochem..

[5] Benson MD (2018). Amyloid nomenclature 2018: recommendations by the International Society of Amyloidosis (ISA) nomenclature committee. Amyloid.

[6] Braak H (2013). Amyotrophic lateral sclerosis-a model of corticofugal axonal spread. Nat. Rev. Neurol..

[7] Goedert M, Eisenberg DS, Crowther RA (2017). Propagation of tau aggregates and neurodegeneration. Annu. Rev. Neurosci..

[8] Braak H, Alafuzoff I, Arzberger T, Kretzschmar H, Del Tredici K (2006). Staging of Alzheimer disease-associated neurofibrillary pathology using paraffin sections and immunocytochemistry. Acta Neuropathol..

[9] Fu H, Hardy J, Duff KE (2018). Selective vulnerability in neurodegenerative diseases. Nat. Neurosci..

[10] Bussiere T (2003). Progressive degeneration of nonphosphorylated neurofilament protein-enriched pyramidal neurons predicts cognitive impairment in Alzheimer’s disease: stereologic analysis of prefrontal cortex area 9. J. Comp. Neurol..

[11] Sepulcre J (2017). Hierarchical organization of tau and amyloid deposits in the cerebral cortex. JAMA Neurol..

[12] Hardy J (2016). Catastrophic cliffs: a partial suggestion for selective vulnerability in neurodegenerative diseases. Biochem. Soc. Trans..

[13] Roussarie JP (2020). Selective neuronal vulnerability in Alzheimer’s disease: a network-based analysis. Neuron.

[14] Muratore CR (2017). Cell-type dependent Alzheimer’s disease phenotypes: probing the biology of selective neuronal vulnerability. Stem Cell Rep..

[15] Jaunmuktane Z, Brandner S (2019). On the journey to uncover the causes of selective cellular and regional vulnerability in neurodegeneration. Acta Neuropathol..

[16] Akila Parvathy Dharshini S, Taguchi YH, Michael Gromiha M (2019). Exploring the selective vulnerability in Alzheimer disease using tissue specific variant analysis. Genomics.

[17] Keo A (2020). Transcriptomic signatures of brain regional vulnerability to Parkinson’s disease. Commun. Biol..

[18] Saxena S, Caroni P (2011). Selective neuronal vulnerability in neurodegenerative diseases: from stressor thresholds to degeneration. Neuron.

[19] Konstantoulea, K., Louros, N., Rousseau, F. & Schymkowitz, J. Heterotypic interactions in amyloid function and disease. *FEBS J*. 10.1111/febs.15719 (2021). 10.1111/febs.15719 33460517

[20] Chin J (2007). Reelin depletion in the entorhinal cortex of human amyloid precursor protein transgenic mice and humans with Alzheimer’s disease. J. Neurosci..

[21] Pujadas, L. et al. Reelin delays amyloid-beta fibril formation and rescues cognitive deficits in a model of Alzheimer’s disease. *Nat. Commun.* **5**, 3443 (2014). 10.1038/ncomms4443 24599114

[22] Cuchillo-Ibanez I (2016). The beta-amyloid peptide compromises Reelin signaling in Alzheimer’s disease. Sci. Rep..

[23] Banerjee S, Ferdosh S, Ghosh AN, Barat C (2020). Tau protein- induced sequestration of the eukaryotic ribosome: Implications in neurodegenerative disease. Sci. Rep..

[24] Pathak BK, Mondal S, Banerjee S, Ghosh AN, Barat C (2017). Sequestration of ribosome during protein aggregate formation: contribution of ribosomal RNA. Sci. Rep..

[25] Azizi SA, Azizi SA (2018). Synucleinopathies in neurodegenerative diseases: accomplices, an inside job and selective vulnerability. Neurosci. Lett..

[26] Horvath I (2018). Co-aggregation of pro-inflammatory S100A9 with alpha-synuclein in Parkinson’s disease: ex vivo and in vitro studies. J. Neuroinflammation.

[27] Ryan BJ, Hoek S, Fon EA, Wade-Martins R (2015). Mitochondrial dysfunction and mitophagy in Parkinson’s: from familial to sporadic disease. Trends Biochem Sci..

[28] Ciryam P, Kundra R, Morimoto RI, Dobson CM, Vendruscolo M (2015). Supersaturation is a major driving force for protein aggregation in neurodegenerative diseases. Trends Pharmacol. Sci..

[29] Hardy J (2005). Expression of normal sequence pathogenic proteins for neurodegenerative disease contributes to disease risk: ‘permissive templating’ as a general mechanism underlying neurodegeneration. Biochem. Soc. Trans..

[30] Ciryam P, Tartaglia GG, Morimoto RI, Dobson CM, Vendruscolo M (2013). Widespread aggregation and neurodegenerative diseases are associated with supersaturated proteins. Cell Rep..

[31] Freer R (2019). Supersaturated proteins are enriched at synapses and underlie cell and tissue vulnerability in Alzheimer’s disease. Heliyon.

[32] Ciryam P (2016). A transcriptional signature of Alzheimer’s disease is associated with a metastable subproteome at risk for aggregation. Proc. Natl Acad. Sci. USA.

[33] Fitzpatrick AWP (2017). Cryo-EM structures of tau filaments from Alzheimer’s disease. Nature.

[34] Scheres SH, Zhang W, Falcon B, Goedert M (2020). Cryo-EM structures of tau filaments. Curr. Opin. Struct. Biol..

[35] Strohaker T (2019). Structural heterogeneity of alpha-synuclein fibrils amplified from patient brain extracts. Nat. Commun..

[36] Landreh M (2016). The formation, function and regulation of amyloids: insights from structural biology. J. Intern. Med..

[37] Riek R, Eisenberg DS (2016). The activities of amyloids from a structural perspective. Nature.

[38] Lutter L, Serpell CJ, Tuite MF, Xue WF (2019). The molecular lifecycle of amyloid - Mechanism of assembly, mesoscopic organisation, polymorphism, suprastructures, and biological consequences. Biochim. Biophys. Acta Proteins Proteom..

[39] Gallardo R, Ranson NA, Radford SE (2020). Amyloid structures: much more than just a cross-beta fold. Curr. Opin. Struct. Biol..

[40] van der Kant, R., Louros, N., Schymkowitz, J. & Rousseau, F. A structural analysis of amyloid polymorphism in disease: clues for selective vulnerability? *bioRxiv*10.1101/2021.03.01.433317 (2021).

[41] Marshall KE (2016). A critical role for the self-assembly of Amyloid-beta1-42 in neurodegeneration. Sci. Rep..

[42] Ganesan A (2016). Structural hot spots for the solubility of globular proteins. Nat. Commun..

[43] Ventura S (2004). Short amino acid stretches can mediate amyloid formation in globular proteins: the Src homology 3 (SH3) case. Proc. Natl Acad. Sci. USA.

[44] Teng PK, Eisenberg D (2009). Short protein segments can drive a non-fibrillizing protein into the amyloid state. Protein Eng. Des. Sel..

[45] Louros N, Orlando G, De Vleeschouwer M, Rousseau F, Schymkowitz J (2020). Structure-based machine-guided mapping of amyloid sequence space reveals uncharted sequence clusters with higher solubilities. Nat. Commun..

[46] Wetzel R (2006). Kinetics and thermodynamics of amyloid fibril assembly. Acc. Chem. Res..

[47] O’Nuallain B, Shivaprasad S, Kheterpal I, Wetzel R (2005). Thermodynamics of A beta(1-40) amyloid fibril elongation. Biochemistry.

[48] O’Nuallain B, Williams AD, Westermark P, Wetzel R (2004). Seeding specificity in amyloid growth induced by heterologous fibrils. J. Biol. Chem..

[49] Krebs MR, Morozova-Roche LA, Daniel K, Robinson CV, Dobson CM (2004). Observation of sequence specificity in the seeding of protein amyloid fibrils. Protein Sci..

[50] Vanik DL, Surewicz KA, Surewicz WK (2004). Molecular basis of barriers for interspecies transmissibility of mammalian prions. Mol. Cell.

[51] Giasson BI (2003). Initiation and synergistic fibrillization of tau and alpha-synuclein. Science.

[52] Oskarsson ME (2015). In vivo seeding and cross-seeding of localized amyloidosis: a molecular link between type 2 diabetes and Alzheimer disease. Am. J. Pathol..

[53] Ly H (2021). The association of circulating amylin with β-amyloid in familial Alzheimer’s disease. Alzheimer’s Dement..

[54] Apostol MI, Wiltzius JJ, Sawaya MR, Cascio D, Eisenberg D (2011). Atomic structures suggest determinants of transmission barriers in mammalian prion disease. Biochemistry.

[55] Krotee P (2018). Common fibrillar spines of amyloid-beta and human islet amyloid polypeptide revealed by microelectron diffraction and structure-based inhibitors. J. Biol. Chem..

[56] Vasconcelos B (2016). Heterotypic seeding of Tau fibrillization by pre-aggregated Abeta provides potent seeds for prion-like seeding and propagation of Tau-pathology in vivo. Acta Neuropathol..

[57] Colom-Cadena M (2013). Confluence of alpha-synuclein, tau, and beta-amyloid pathologies in dementia with Lewy bodies. J. Neuropathol. Exp. Neurol..

[58] Pham, C. L. et al. Viral M45 and necroptosis-associated proteins form heteromeric amyloid assemblies. *EMBO Rep.*10.15252/embr.201846518 (2019). 10.15252/embr.201846518 PMC6362354 30498077

[59] Sampson, T. R. et al. A gut bacterial amyloid promotes alpha-synuclein aggregation and motor impairment in mice. *Elife*10.7554/eLife.53111 (2020). 10.7554/eLife.53111 PMC7012599 32043464

[60] Collinson SK, Parker JM, Hodges RS, Kay WW (1999). Structural predictions of AgfA, the insoluble fimbrial subunit of Salmonella thin aggregative fimbriae. J. Mol. Biol..

[61] Louros NN, Bolas GMP, Tsiolaki PL, Hamodrakas SJ, Iconomidou VA (2016). Intrinsic aggregation propensity of the CsgB nucleator protein is crucial for curli fiber formation. J. Struct. Biol..

[62] White AP (2001). Structure and characterization of AgfB from Salmonella enteritidis thin aggregative fimbriae. J. Mol. Biol..

[63] Louros NN (2016). A common ‘aggregation-prone’ interface possibly participates in the self-assembly of human zona pellucida proteins. FEBS Lett..

[64] Li J (2012). The RIP1/RIP3 necrosome forms a functional amyloid signaling complex required for programmed necrosis. Cell.

[65] Wu XN (2014). Distinct roles of RIP1-RIP3 hetero- and RIP3-RIP3 homo-interaction in mediating necroptosis. Cell Death Differ..

[66] Petrie EJ (2019). Viral MLKL homologs subvert necroptotic cell death by sequestering cellular RIPK3. Cell Rep..

[67] Wolozin B (2012). Regulated protein aggregation: stress granules and neurodegeneration. Mol. Neurodegener..

[68] Wolozin B, Ivanov P (2019). Stress granules and neurodegeneration. Nat. Rev. Neurosci..

[69] Fabiani C, Antollini SS (2019). Alzheimer’s disease as a membrane disorder: spatial cross-talk among beta-amyloid peptides, nicotinic acetylcholine receptors and lipid rafts. Front. Cell. Neurosci..

[70] Stewart KL (2016). Atomic details of the interactions of glycosaminoglycans with amyloid-beta fibrils. J. Am. Chem. Soc..

[71] Cohen SIA (2015). A molecular chaperone breaks the catalytic cycle that generates toxic Abeta oligomers. Nat. Struct. Mol. Biol..

[72] Derkatch IL (2004). Effects of Q/N-rich, polyQ, and non-polyQ amyloids on the de novo formation of the [PSI+] prion in yeast and aggregation of Sup35 in vitro. Proc. Natl Acad. Sci. USA.

[73] Mompean M (2018). The structure of the necrosome RIPK1-RIPK3 core, a human hetero-amyloid signaling complex. Cell.

[74] Sidhu A, Segers-Nolten I, Subramaniam V (2016). Conformational compatibility is essential for heterologous aggregation of alpha-synuclein. ACS Chem. Neurosci..

[75] Wasmer C (2010). Structural similarity between the prion domain of HET-s and a homologue can explain amyloid cross-seeding in spite of limited sequence identity. J. Mol. Biol..

[76] Benkemoun L (2011). Two structurally similar fungal prions efficiently cross-seed in vivo but form distinct polymers when coexpressed. Mol. Microbiol.

[77] Schymkowitz J (2005). The FoldX web server: an online force field. Nucleic Acids Res..

[78] Khodaparast L (2018). Aggregating sequences that occur in many proteins constitute weak spots of bacterial proteostasis. Nat. Commun..

[79] Michiels E (2020). Reverse engineering synthetic antiviral amyloids. Nat. Commun..

[80] Houben B (2020). Autonomous aggregation suppression by acidic residues explains why chaperones favour basic residues. EMBO J..

[81] Richardson JS, Richardson DC (2002). Natural beta-sheet proteins use negative design to avoid edge-to-edge aggregation. Proc. Natl Acad. Sci. USA.

[82] Banach, M. & Roterman, I. in *From Globular Proteins to Amyloids* (ed. Roterman-Konieczna, I.) 95–115 (Elsevier, 2020).

[83] Kajava AV, Steven AC (2006). Beta-rolls, beta-helices, and other beta-solenoid proteins. Adv. Protein Chem..

[84] Louros NN, Baltoumas FA, Hamodrakas SJ, Iconomidou VA (2016). A beta-solenoid model of the Pmel17 repeat domain: insights to the formation of functional amyloid fibrils. J. Comput Aided Mol. Des..

[85] Sawaya MR (2007). Atomic structures of amyloid cross-beta spines reveal varied steric zippers. Nature.

[86] von Bergen M, Barghorn S, Biernat J, Mandelkow EM, Mandelkow E (2005). Tau aggregation is driven by a transition from random coil to beta sheet structure. Biochim. Biophys. Acta.

[87] Seidler PM (2018). Structure-based inhibitors of tau aggregation. Nat. Chem..

[88] Louros N (2020). WALTZ-DB 2.0: an updated database containing structural information of experimentally determined amyloid-forming peptides. Nucleic Acids Res..

[89] Louros NN (2015). Chameleon ‘aggregation-prone’ segments of apoA-I: A model of amyloid fibrils formed in apoA-I amyloidosis. Int. J. Biol. Macromol..

[90] Blancas-Mejia LM, Ramirez-Alvarado M (2016). Recruitment of light chains by homologous and heterologous fibrils shows distinctive kinetic and conformational specificity. Biochemistry.

[91] Liberta F (2019). Cryo-EM fibril structures from systemic AA amyloidosis reveal the species complementarity of pathological amyloids. Nat. Commun..

[92] Cao Q, Boyer DR, Sawaya MR, Ge P, Eisenberg DS (2020). Cryo-EM structure and inhibitor design of human IAPP (amylin) fibrils. Nat. Struct. Mol. Biol..

[93] Sun Y (2020). Cryo-EM structure of full-length alpha-synuclein amyloid fibril with Parkinson’s disease familial A53T mutation. Cell Res..

[94] Sgourakis NG, Yau WM, Qiang W (2015). Modeling an in-register, parallel “iowa” Aβ fibril structure using solid-state NMR data from labeled samples with rosetta. Structure.

[95] Schutz AK (2015). Atomic-resolution three-dimensional structure of amyloid beta fibrils bearing the Osaka mutation. Angew. Chem. Int. Ed. Engl..

[96] Gallardo, R. et al. Fibril structures of diabetes-related amylin variants reveal a basis for surface-templated assembly. *Nat. Struct. Mol. Biolo.*10.1038/s41594-020-0496-3 (2020). 10.1038/s41594-020-0496-3 PMC7617688 32929282

[97] Condello C (2018). Structural heterogeneity and intersubject variability of Abeta in familial and sporadic Alzheimer’s disease. Proc. Natl Acad. Sci. USA.

[98] Kar K, Arduini I, Drombosky KW, van der Wel PC, Wetzel R (2014). D-polyglutamine amyloid recruits L-polyglutamine monomers and kills cells. J. Mol. Biol..

[99] Kapurniotu A, Schmauder A, Tenidis K (2002). Structure-based design and study of non-amyloidogenic, double N-methylated IAPP amyloid core sequences as inhibitors of IAPP amyloid formation and cytotoxicity. J. Mol. Biol..

[100] Tatarek-Nossol M (2005). Inhibition of hIAPP amyloid-fibril formation and apoptotic cell death by a designed hIAPP amyloid- core-containing hexapeptide. Chem. Biol..

[101] Yan LM (2013). Selectively N-methylated soluble IAPP mimics as potent IAPP receptor agonists and nanomolar inhibitors of cytotoxic self-assembly of both IAPP and Abeta40. Angew. Chem. Int. Ed. Engl..

[102] Sangwan, S. et al. Inhibition of synucleinopathic seeding by rationally designed inhibitors. *Elife*10.7554/eLife.46775 (2020). 10.7554/eLife.46775 PMC6977966 31895037

[103] Seidler PM (2019). Structure-based inhibitors halt prion-like seeding by Alzheimer’s disease-and tauopathy-derived brain tissue samples. J. Biol. Chem..

[104] Saelices L (2019). A pair of peptides inhibits seeding of the hormone transporter transthyretin into amyloid fibrils. J. Biol. Chem..

[105] Lu J (2019). Structure-based peptide inhibitor design of amyloid-beta aggregation. Front. Mol. Neurosci..

[106] Griner, S. L. et al. Structure-based inhibitors of amyloid beta core suggest a common interface with tau. *Elife*10.7554/eLife.46924 (2019). 10.7554/eLife.46924 PMC6850776 31612856

[107] Cheng PN, Liu C, Zhao M, Eisenberg D, Nowick JS (2012). Amyloid beta-sheet mimics that antagonize protein aggregation and reduce amyloid toxicity. Nat. Chem..

[108] Gallardo, R. et al. De novo design of a biologically active amyloid. *Science*10.1126/science.aah4949 (2016). 10.1126/science.aah4949 27846578

[109] Holmes BB (2014). Proteopathic tau seeding predicts tauopathy in vivo. Proc. Natl Acad. Sci. USA.

[110] Mathieu C, Pappu RV, Taylor JP (2020). Beyond aggregation: pathological phase transitions in neurodegenerative disease. Science.

[111] Brunello CA, Yan X, Huttunen HJ (2016). Internalized Tau sensitizes cells to stress by promoting formation and stability of stress granules. Sci. Rep..

[112] Gui X (2019). Structural basis for reversible amyloids of hnRNPA1 elucidates their role in stress granule assembly. Nat. Commun..

[113] Ray S (2020). alpha-Synuclein aggregation nucleates through liquid-liquid phase separation. Nat. Chem..

[114] Jha NK (2015). Impact of insulin degrading enzyme and neprilysin in alzheimer’s disease biology: characterization of putative cognates for therapeutic applications. J. Alzheimers Dis..

[115] Kurochkin IV, Guarnera E, Berezovsky IN (2018). Insulin-degrading enzyme in the fight against Alzheimer’s disease. Trends Pharmacol. Sci..

[116] Sahoo BR (2021). Degradation of Alzheimer’s amyloid-beta by a catalytically inactive insulin-degrading enzyme. J. Mol. Biol..

[117] Chen Q (2001). Presenilin binding protein is associated with neurofibrillary alterations in Alzheimer’s disease and stimulates tau phosphorylation. Am. J. Pathol..

[118] Liu XY (2015). Regulation of RAGE splicing by hnRNP A1 and Tra2beta-1 and its potential role in AD pathogenesis. J. Neurochem..

[119] Wong J (2013). Altered expression of RNA splicing proteins in Alzheimer’s disease patients: evidence from two microarray studies. Dement Geriatr. Cogn. Dis. Extra.

[120] Storbeck M (2014). Neuronal-specific deficiency of the splicing factor Tra2b causes apoptosis in neurogenic areas of the developing mouse brain. PLoS ONE.

[121] Lester, E. et al. Tau aggregates are RNA-protein assemblies that mislocalize multiple nuclear speckle components. *Neuron*10.1016/j.neuron.2021.03.026 (2021). 10.1016/j.neuron.2021.03.026 PMC8141031 33848474

[122] Öhrfelt A (2016). The pre-synaptic vesicle protein synaptotagmin is a novel biomarker for Alzheimer’s disease. Alzheimer’s Res. Ther..

[123] Keller LJ (2020). Presenilin 1 increases association with synaptotagmin 1 during normal aging. Neurobiol. Aging.

[124] Zoltowska KM (2017). Dynamic presenilin 1 and synaptotagmin 1 interaction modulates exocytosis and amyloid β production. Mol. Neurodegener..

[125] Iwasawa S (2019). Recurrent de novo MAPK8IP3 variants cause neurological phenotypes. Ann. Neurol..

[126] Gowrishankar, S. et al. Overlapping roles of JIP3 and JIP4 in promoting axonal transport of lysosomes in human iPSC-derived neurons. *bioRxiv*10.1101/2020.06.13.149443 (2021). 10.1091/mbc.E20-06-0382 PMC8351540 33788575

[127] Berman HM (2000). The Protein Data Bank. Nucleic Acids Res..

[128] Tien MZ, Meyer AG, Sydykova DK, Spielman SJ, Wilke CO (2013). Maximum allowed solvent accessibilites of residues in proteins. PLoS ONE.

[129] Tao P, Wang R, Lai L (1999). Calculating partition coefficients of peptides by the addition method. Mol. Modeling Annu..

[130] Chou PY, Fasman GD (1978). Prediction of the secondary structure of proteins from their amino acid sequence. Adv. Enzymol. Relat. Areas Mol. Biol..

[131] Savitzky A, Golay MJE (1964). Smoothing and differentiation of data by simplified least squares procedures. Anal. Chem..

[132] Mirbaha, H. et al. Inert and seed-competent tau monomers suggest structural origins of aggregation. *Elife*10.7554/eLife.36584 (2018). 10.7554/eLife.36584 PMC6039173 29988016

[133] Goedert M, Spillantini MG, Cairns NJ, Crowther RA (1992). Tau proteins of Alzheimer paired helical filaments: abnormal phosphorylation of all six brain isoforms. Neuron.

