# Supplementary material for: Mapping the sequence specificity of heterotypic amyloid interactions enables the identification of aggregation modifiers
Source: Nat Commun. 2022 Mar 15;13:1351. doi: 10.1038/s41467-022-28955-9 (PMC8924238; doi:10.1038/s41467-022-28955-9)
Supplement: Supplementary file 1 — Supplementary Information [file 41467_2022_28955_MOESM1_ESM.pdf]

Supplementary Information

Mapping the sequence specificity of heterotypic amyloid interactions enables the identification of aggregation modifiers

Nikolaos Louros[1,2], Meine Ramakers[1,2], Emiel Michiels[1,2], Katerina Konstantoulea[1,2], Chiara Morelli[1,2], Teresa Garcia[1,2], Nele Moonen[1,2], Sam D'Haeyer[3,4], Vera Goossens[3,4], Dietmar Rudolf Thal[5,6], Dominique Audenaert[3,4], Frederic Rousseau[1,2]*, Joost Schymkowitz[1,2]*

[1]Switch Laboratory, VIB Center for Brain and Disease Research, Herestraat 49, 3000, Leuven, Belgium.

[2]Switch Laboratory, Department of Cellular and Molecular Medicine, KULeuven, Herestraat 49, 3000, Leuven, Belgium.

[3]Screening Core, VIB, Ghent, Belgium

[4]Centre for Bioassay Development and Screening (C-BIOS), Ghent University, Ghent, Belgium

[5]KU Leuven, Leuven Brain Institute, 3000 Leuven, Belgium

[6]Laboratory for Neuropathology, KU Leuven, and Department of Pathology, UZ Leuven, 3000 Leuven, Belgium

This PDF file includes:

Supplementary Figures 1-8
Supplementary Tables 1-3
Supplementary Methods
Supplementary Reference List

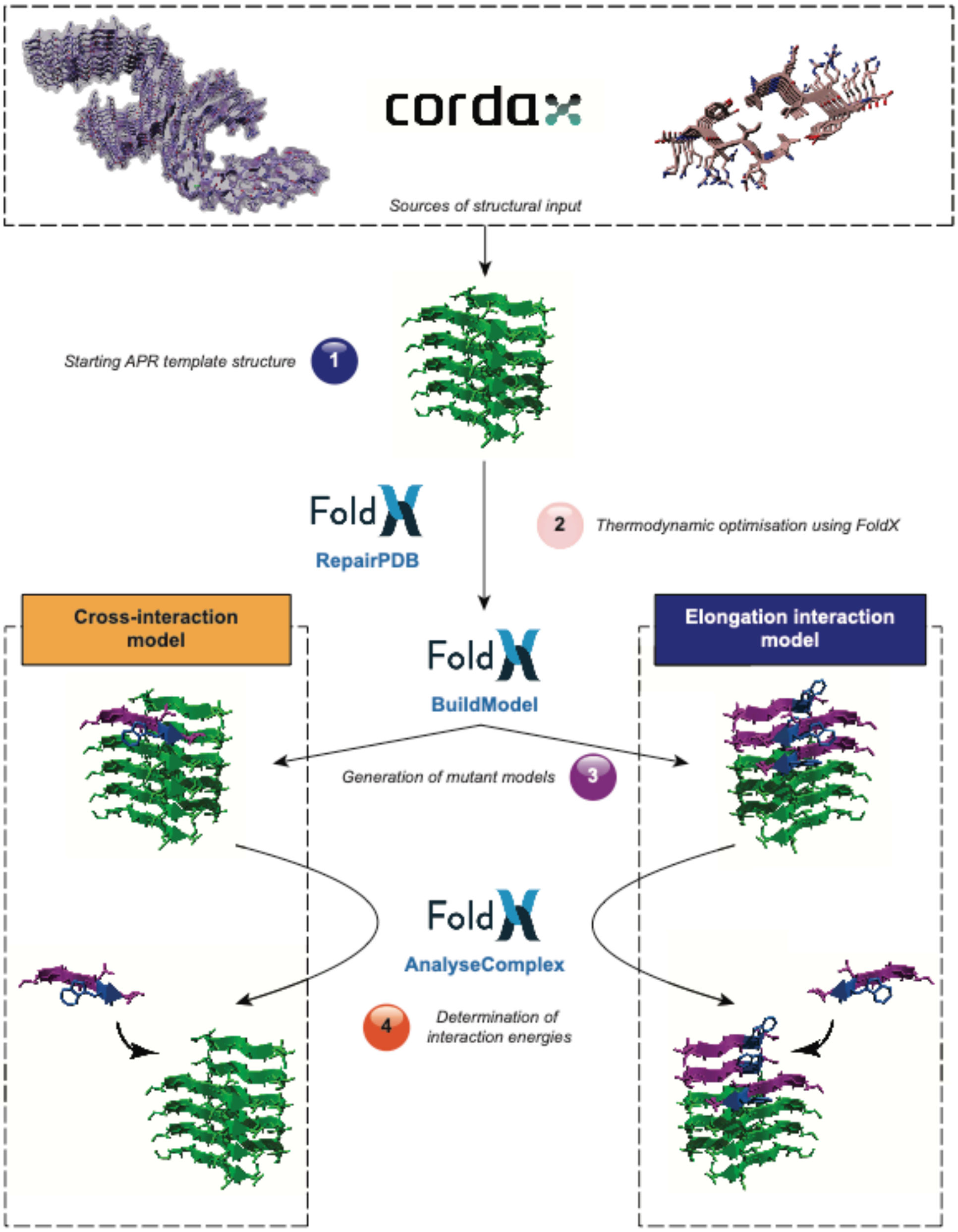

Supplementary Figure 1. Protocol capture of the approach used to determine the thermodynamic potential of heterotypic amyloid interactions. Briefly, (1) the structural architecture of an APR segment can be isolated from various sources, including steric zipper structures, structures of full-length amyloid fibrils or with the use of state-of-art computational tools that provide structural topologies of APRs, such as CORDAX[1]. (2) Subsequent side chain optimisation repairs potential bad torsion angles and clashes using the rotamers library included in FoldX[2]. (3) The derived template is then used to generate one cross-interaction and one elongation model per mutant by variating the corresponding positions of the edge (cross-interaction model, purple chain) and edge and next-to-edge peptide chains (elongation model, purple chains), respectively. Finally, the generated models are utilised to calculate the cross-interaction and elongation interaction potentials for the edge mutated chains, using the AnalyseComplex function available in FoldX[2].

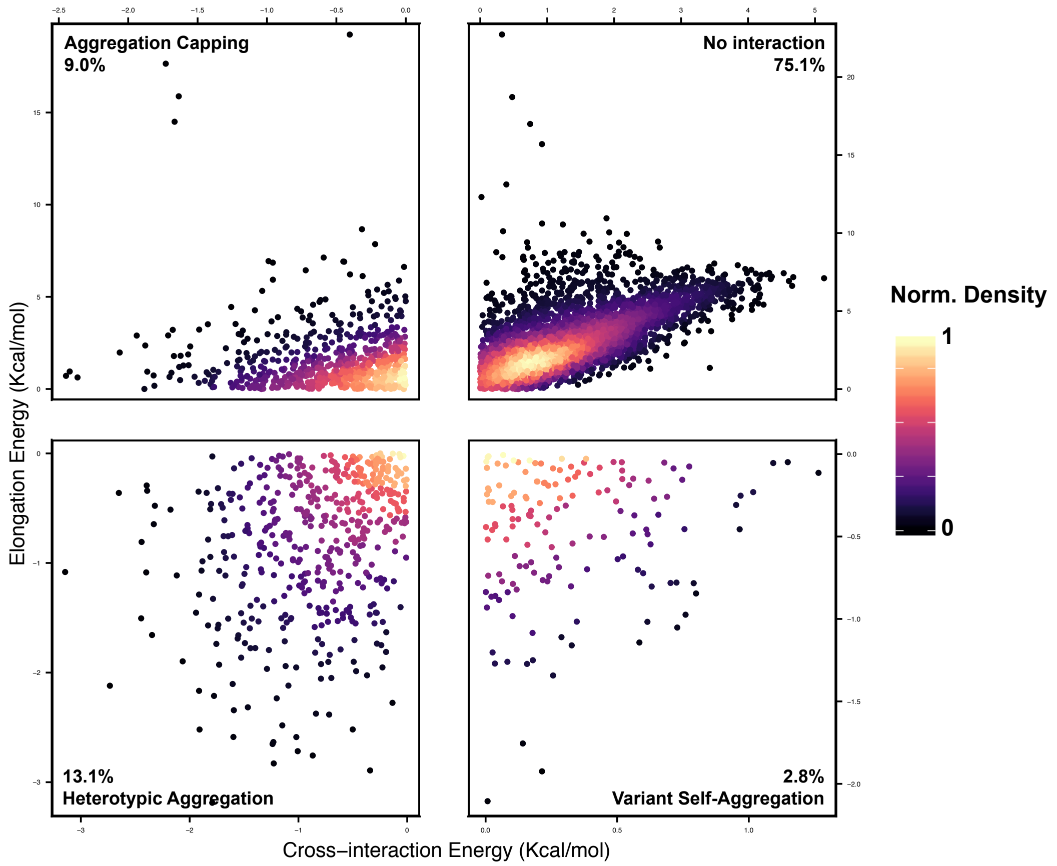

Supplementary Figure 2. Thermodynamic profile of all possible double variants for VQIVYK. Distribution analysis indicates that a smaller fraction of double variants is compatible to heterotypic or even self-associating interactions.

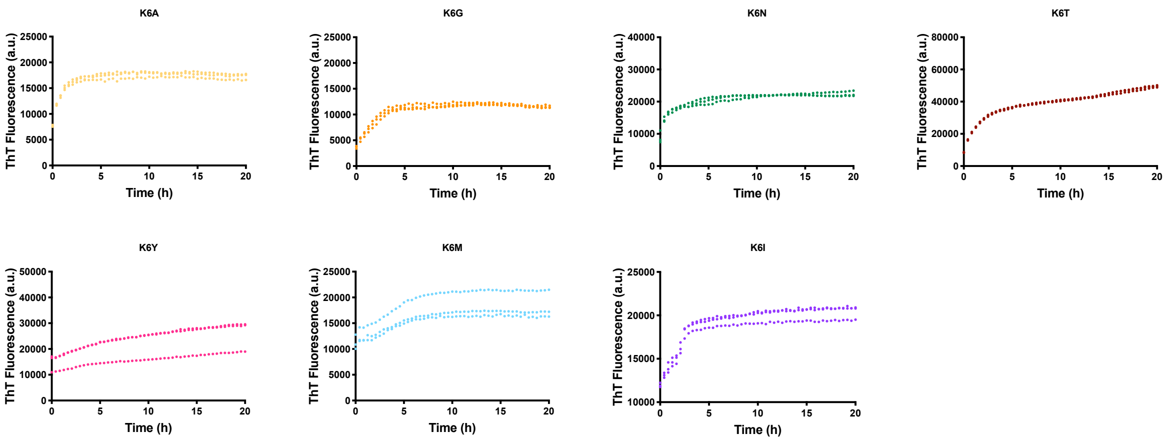

Supplementary Figure 3. Th-T kinetics, performed in triplicates at a concentration of 25μM, for variants corresponding to the exposed Lys residue of VQIVYK. Source data are provided as a Source Data file.

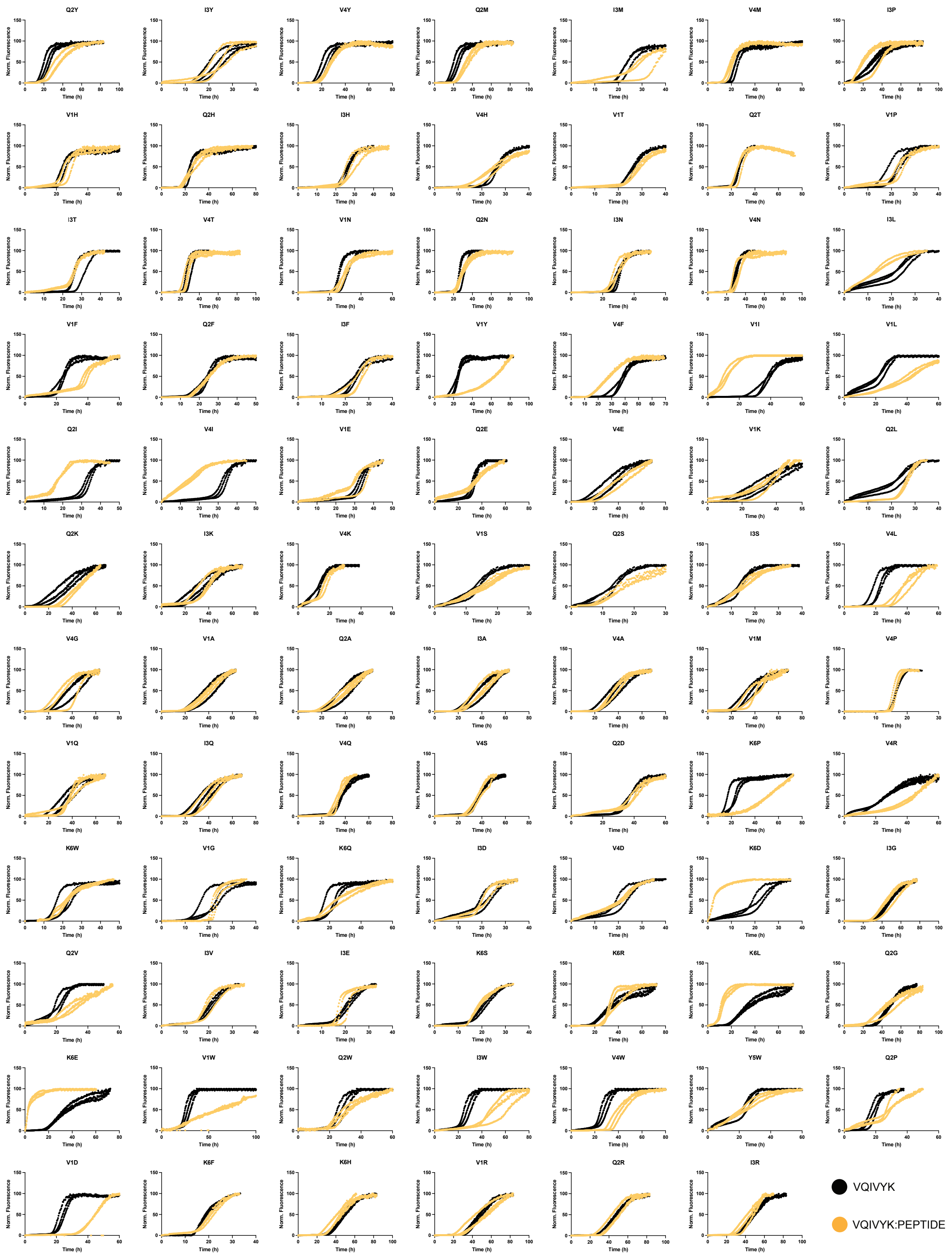

Supplementary Figure 4. Th-T curves of the VQIVYK peptide mixed (1:5) with the library variants.

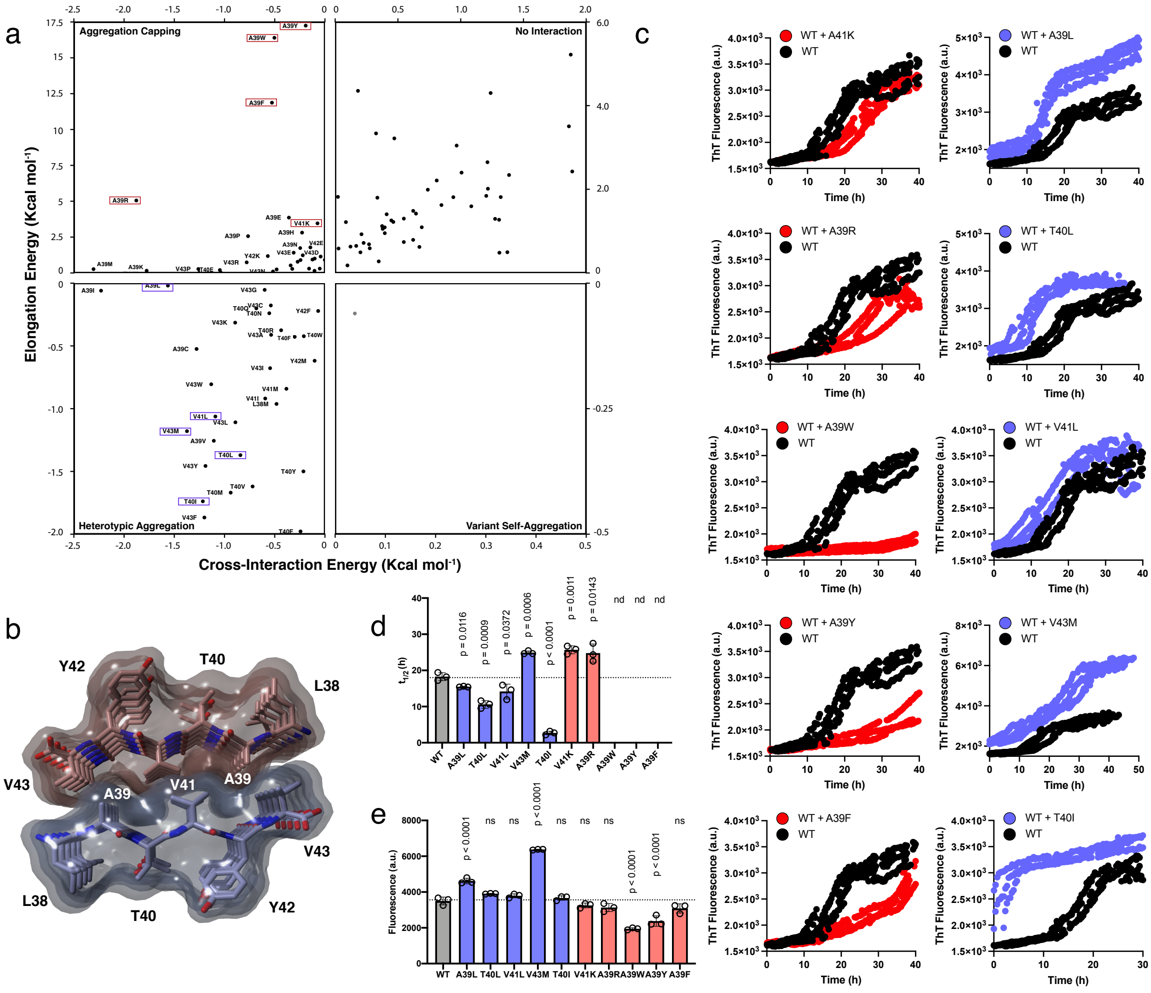

Supplementary Figure 5. Thermodynamic profiling using a three-dimensional model template of the ^{38}LATVYV^{43} APR from ApoA-I. (a) Thermodynamic profiling of cross-interaction propensities for single position homologs. Highlighted in red and purple boxes are randomly selected sequences that were experimentally tested. (b) Topological model of the aggregation core formed by the ^{38}LATVYV^{43} APR, developed using CORDAX[1]. (c) Th-T kinetics, performed in triplicates, as well as (d) halftime and (e) endpoint fluorescence analysis, validated that all 10 peptides either capped or promoted aggregation of the APR, as depicted by the thermodynamic analysis. Data are shown as mean values +/- SD (n= 3 biologically independent samples). Statistical significance was determined using one-way ANOVA with Tukey's test for multiple comparisons to the WT. Source data are provided as a Source Data file.

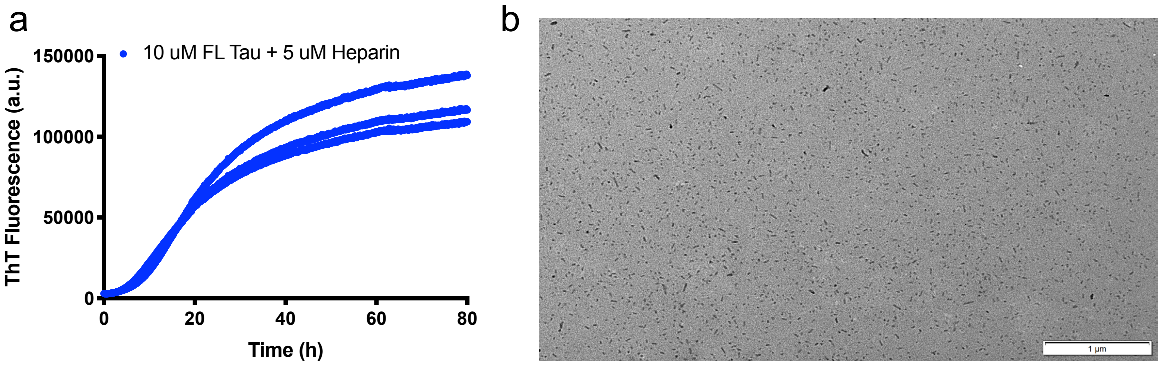

Supplementary Figure 6. Preparation of recombinant full-length (2N4R) tau seeds. (a) Th-T kinetics were performed in triplicates to monitor the aggregation of full-length tau^{2N4R} at 10μM over time. (b) Electron micrographs (n= 3 independent repeats) validate that uniform tau seeds are formed after sonication of end-state tau amyloid fibrils. Source data are provided as a Source Data file.

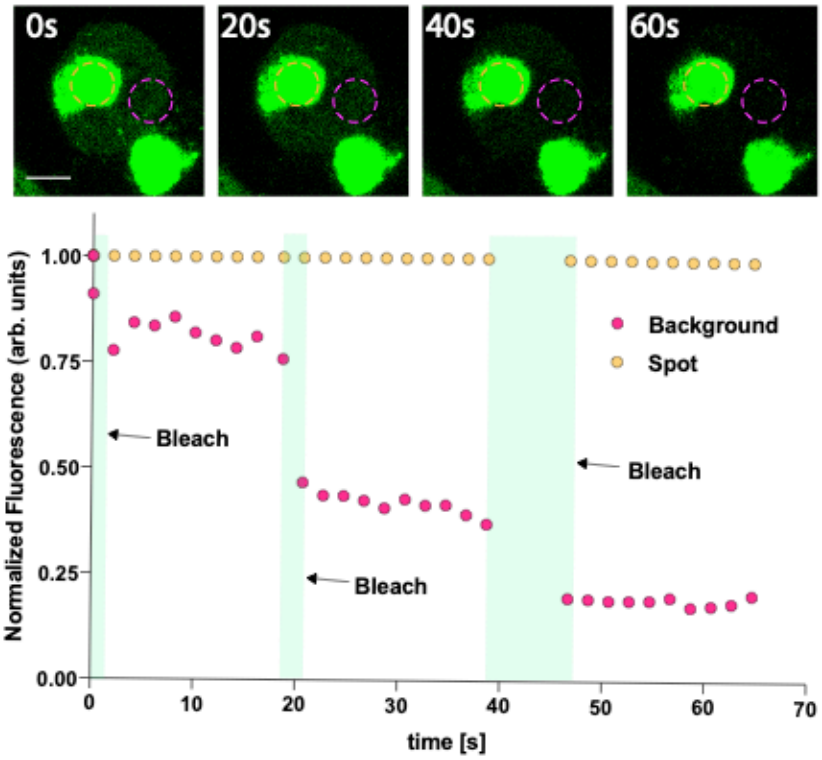

Supplementary Figure 7. Fluorescence recovery after photobleaching (FRAP) measurements of tau inclusions in the FRET biosensors. Fluorescence recovery was measured for a region defined within a tau spot (yellow ROI) and a region within the cytoplasm of the cell (magenta ROI), after successive bleaching steps performed in the cytoplasm (Scale bar = 5μm). Individual timeframes validate that the background of the cell containing soluble tau is bleached, whereas fluorescence of the puncta remains unaffected indicating that there is minimal trade-off with the soluble protein in the cell.

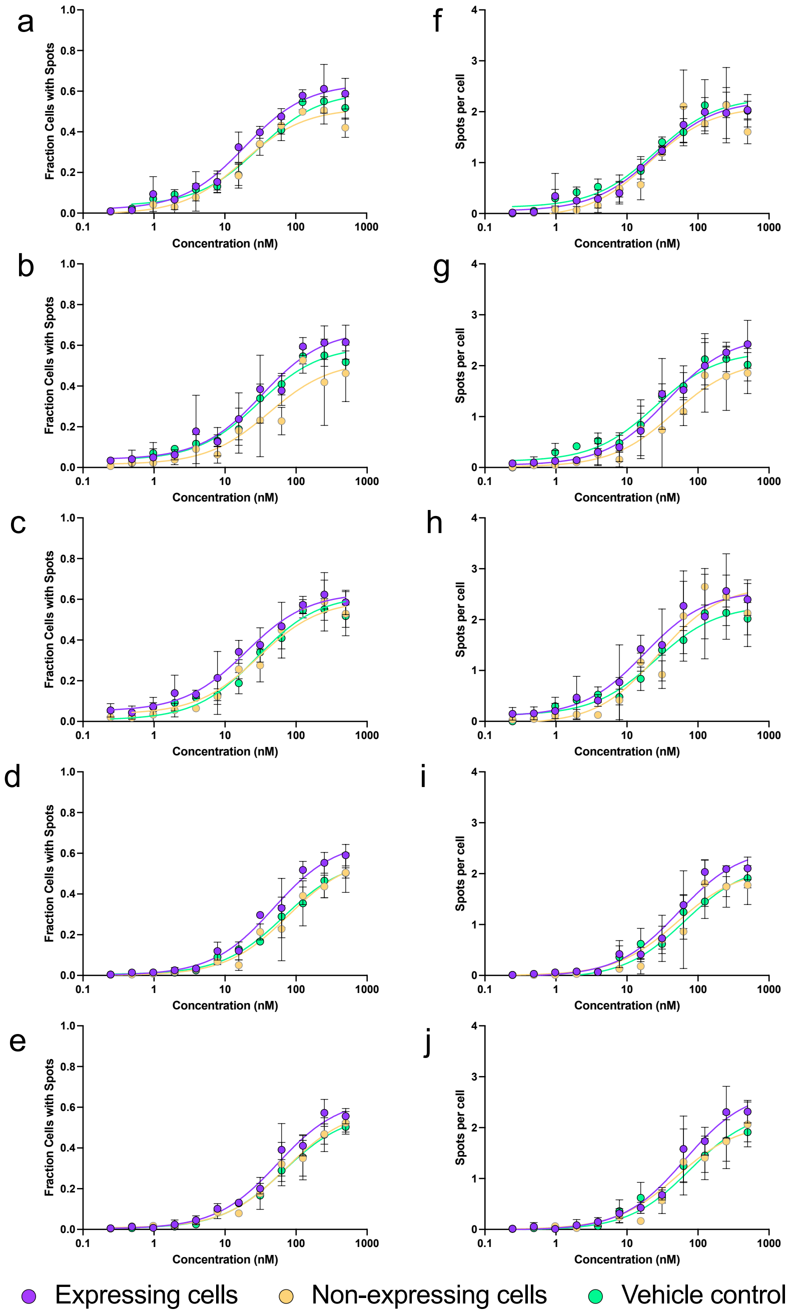

Supplementary Figure 8. Construct co-expression in the FRET biosensor, followed by concentration-dependent tau seeding. Number of cells with spots (a-e) and number of spots per cell (f-j) that were identified in the FRET cell line for a concentration gradient of tau seeds, following transfection with (a, f) RAB3GAP1, (b, g) CALY, (c, h) NPAS3(d, i) MSR1 and (e, j) TIAM1, respectively. Minimal differences compared to the vehicle and non-expressing cells indicate that expression of these constructs does not affect tau aggregation and spreading. Experiments were performed in triplicates as individual 96-well plate preparations. Data are represented as mean values +/- SD. Source data are provided as a Source Data file.

Supplementary Tables

Supplementary Table 1. List of the amyloid core fibril structures[1].

PDB ID	Sequence	Protein
1yjo [https://www.rcsb.org/structure/1yjo]	NNQQNY	Sup35
1yjp [https://www.rcsb.org/structure/1yjp]	GNNQQNY	Sup35
2m5n [https://www.rcsb.org/structure/2m5n]	YTIAALLSPYS	Transthyretin (TTR)
2okz [https://www.rcsb.org/structure/2okz]	MVGGVV	Amyloid-beta (Aβ)
2ol9 [https://www.rcsb.org/structure/2ol9]	SNQNNF	Major Prion protein (PrP)
2olx [https://www.rcsb.org/structure/2olx]	NNQQ	Sup35
2omm [https://www.rcsb.org/structure/2omm]	GNNQQNY	Sup35
2omp [https://www.rcsb.org/structure/2omp]	LYQLEN	Insulin
2omq [https://www.rcsb.org/structure/2omq]	VEALYL	Insulin
2on9 [https://www.rcsb.org/structure/2on9]	VQIVYK	Tau
2ona [https://www.rcsb.org/structure/2ona]	MVGGVV	Amyloid-beta (Aβ)
2onv [https://www.rcsb.org/structure/2onv]	GGVVIA	Amyloid-beta (Aβ)
2onw [https://www.rcsb.org/structure/2onw]	SSTSSA	Bovine Pancreatic Ribonuclease (RNase A)
2onx [https://www.rcsb.org/structure/2onx]	NNQQ	Sup35
2y29 [https://www.rcsb.org/structure/2y29]	KLVFFA	Amyloid-beta (Aβ)
2y2a [https://www.rcsb.org/structure/2y2a]	KLVFFA	Amyloid-beta (Aβ)
2y3j [https://www.rcsb.org/structure/2y3j]	AIIGLM	Amyloid-beta (Aβ)
2y3k [https://www.rcsb.org/structure/2y3k]	MVGGVVIA	Amyloid-beta (Aβ)
2y3l [https://www.rcsb.org/structure/2y3l]	MVGGVVIA	Amyloid-beta (Aβ)
3dg1_a [https://www.rcsb.org/structure/3dg1]	SSTNVG	Islet Amyloid Polypeptide (IAPP) – Interface a
3dg1_b [https://www.rcsb.org/structure/3dg1]	SSTNVG	Islet Amyloid Polypeptide (IAPP) – Interface b
3dgj [https://www.rcsb.org/structure/3dgj]	NNFGAIL	Islet Amyloid Polypeptide (IAPP)
3fod [https://www.rcsb.org/structure/3fod]	AILSST	Islet Amyloid Polypeptide (IAPP)
3fpo [https://www.rcsb.org/structure/3fpo]	HSSNNF	Islet Amyloid Polypeptide (IAPP)
3fr1 [https://www.rcsb.org/structure/3fr1]	NFLVHS	Islet Amyloid Polypeptide (IAPP)
3fth [https://www.rcsb.org/structure/3fth]	NFLVHSS	Islet Amyloid Polypeptide (IAPP)
3ftk [https://www.rcsb.org/structure/3ftk]	NVGSNTY	Islet Amyloid Polypeptide (IAPP)
3ftl_a [https://www.rcsb.org/structure/3ftl]	NVGSNTY	Islet Amyloid Polypeptide (IAPP) – Interface a
3ftl_b [https://www.rcsb.org/structure/3ftl]	NVGSNTY	Islet Amyloid Polypeptide (IAPP) – Interface b
3ftr [https://www.rcsb.org/structure/3ftr]	SSTNVG	Islet Amyloid Polypeptide (IAPP)
3fva_a [https://www.rcsb.org/structure/3fva]	NNQNTF	Elk Prion – Interface a
3fva_b [https://www.rcsb.org/structure/3fva]	NNQNTF	Elk Prion – Interface b
3hyd_a [https://www.rcsb.org/structure/3hyd]	LVEALYL	Insulin – Interface a
3hyd_b [https://www.rcsb.org/structure/3hyd]	LVEALYL	Insulin – Interface b
3loz [https://www.rcsb.org/structure/3loz]	LSFSKD	Beta-2-Microglobulin (β2m)
3nhc [https://www.rcsb.org/structure/3nhc]	GYMLGS	Major Prion protein (PrP-M129)
3nhd [https://www.rcsb.org/structure/3nhd]	GYVLGS	Major Prion protein (PrP-V129)

3nve [https://www.rcsb.org/structure/3nve]	MMHFGN	Syrian hamster prion
3ow9 [https://www.rcsb.org/structure/3ow9]	KLVFFA	Amyloid-beta (Aβ)
3ppd_a [https://www.rcsb.org/structure/3ppd]	GGVLVN	Human Prostatic Acid Phosphatase (SEVI) – Interface a
3ppd_b [https://www.rcsb.org/structure/3ppd]	GGVLVN	Human Prostatic Acid Phosphatase (SEVI) – Interface b
3pzz [https://www.rcsb.org/structure/3pzz]	GAIIGL	Amyloid-beta (Aβ)
3q2x_a [https://www.rcsb.org/structure/3q2x]	NKGAII	Amyloid-beta (Aβ) – Interface a
3q2x_b [https://www.rcsb.org/structure/3q2x]	NKGAII	Amyloid-beta (Aβ) – Interface b
3sgs [https://www.rcsb.org/structure/3sgs]	GDVIEV	αB-crystallin
4nin [https://www.rcsb.org/structure/4nin]	DSVISLS	Human Superoxide Dismutase (SOD1)
4nio_a [https://www.rcsb.org/structure/4nio]	GVTGIAQ	Human Superoxide Dismutase (SOD1) – Interface a
4nio_b [https://www.rcsb.org/structure/4nio]	GVTGIAQ	Human Superoxide Dismutase (SOD1) – Interface b
4nip_a [https://www.rcsb.org/structure/4nip]	GVIGIAQ	Human Superoxide Dismutase (SOD1) – Interface a
4nip_b [https://www.rcsb.org/structure/4nip]	GVIGIAQ	Human Superoxide Dismutase (SOD1) – Interface b
4qxx [https://www.rcsb.org/structure/4qxx]	GNLVS	Eosinophil major basic protein (EMBP)
4r0p_a [https://www.rcsb.org/structure/4r0p]	IFQINS	Human Lysozyme – Interface a
4r0p_b [https://www.rcsb.org/structure/4r0p]	IFQINS	Human Lysozyme – Interface b
4r0u_a [https://www.rcsb.org/structure/4r0u]	TGVTAVA	α-synuclein – Interface a
4r0u_b [https://www.rcsb.org/structure/4r0u]	TGVTAVA	α-synuclein – Interface b
4r0w_a [https://www.rcsb.org/structure/4r0w]	VVTGVTA	α-synuclein – Interface a
4r0w_b [https://www.rcsb.org/structure/4r0w]	VVTGVTA	α-synuclein – Interface b
4rik_a [https://www.rcsb.org/structure/4rik]	AVVTGVTAV	α-synuclein – Interface a
4rik_b [https://www.rcsb.org/structure/4rik]	AVVTGVTAV	α-synuclein – Interface b
4ril_a [https://www.rcsb.org/structure/4ril]	GAVVTGVTAVA	α-synuclein – Interface a
4ril_b [https://www.rcsb.org/structure/4ril]	GAVVTGVTAVA	α-synuclein – Interface b
4rp6 [https://www.rcsb.org/structure/4rp6]	LTIITLE	p53
4rp7_a [https://www.rcsb.org/structure/4rp7]	TIITLE	p53 – Interface a
4rp7_b [https://www.rcsb.org/structure/4rp7]	TIITLE	p53 – Interface b
4tut [https://www.rcsb.org/structure/4tut]	GGYMLG	Major Prion protein (PrP)
4uby [https://www.rcsb.org/structure/4uby]	GGYVLG	Major Prion protein (PrP)
4ubz [https://www.rcsb.org/structure/4ubz]	GGYLLG	Major Prion protein (PrP)
4w5l [https://www.rcsb.org/structure/4w5l]	GGYLLGS	Major Prion protein (PrP)
4w5m [https://www.rcsb.org/structure/4w5m]	GGYMLGS	Major Prion protein (PrP)
4w5p [https://www.rcsb.org/structure/4w5p]	GGYVLGS	Major Prion protein (PrP)
4w5y [https://www.rcsb.org/structure/4w5y]	GYMLGSA	Major Prion protein (PrP)
4w67 [https://www.rcsb.org/structure/4w67]	GYVLGSA	Major Prion protein (PrP)
4w71 [https://www.rcsb.org/structure/4w71]	GYLLGSA	Major Prion protein (PrP)
4wbu [https://www.rcsb.org/structure/4wbu]	GYMLGS	Major Prion protein (PrP)
4wbv [https://www.rcsb.org/structure/4wbv]	GYVLGS	Major Prion protein (PrP)
4xfn [https://www.rcsb.org/structure/4xfn]	AEVVFT	Transthyretin (TTR)
4xfo [https://www.rcsb.org/structure/4xfo]	TAVVTN	Transthyretin (TTR)

4znn_a [https://www.rcsb.org/structure/4znn]	GVVHGVTTVA	α-synuclein (A53T) – Interface a
4znn_b [https://www.rcsb.org/structure/4znn]	GVVHGVTTVA	α-synuclein (A53T) – Interface b
5e5v [https://www.rcsb.org/structure/5e5v]	NFGAILS	Islet Amyloid Polypeptide (IAPP)
5e5x [https://www.rcsb.org/structure/5e5x]	ANFLVH	Islet Amyloid Polypeptide (IAPP)
5e5z [https://www.rcsb.org/structure/5e5z]	LVHSSN	Islet Amyloid Polypeptide (IAPP)
5v5c [https://www.rcsb.org/structure/5v5c]	VQIINK	Tau

Supplementary Table 2. List of protein constructs used for transient transfection.

Uniprot ID	Gene	Construct	Construct Length (aa)	Homologous segment	Starting AA	Protein	Description
Q15042 [https://www.uniprot.org/uniprot/Q15042]	RAB3GAP1	HA-RAB3GAP1-3xFLAG-IRES-mKO2	400 (aa 1-400)	VQIHHK	167	Rab3 GTPase-activating protein catalytic subunit	Macroautophagy - Regulated exocytosis of neurotransmitters and hormones
P14735 [https://www.uniprot.org/uniprot/P14735]	IDE	HA-IDE-3xFLAG-IRES-mKO2	300 (aa 403-702) Metalloenzyme, LuxS/M16 peptidase-like domain	VQIVSK	478	Insulin-degrading enzyme	Degrades multiple amyloidogenic proteins (including Aβ)
Q9NYX4 [https://www.uniprot.org/uniprot/Q9NYX4]	CALY	HA-CALY-3xFLAG-IRES-mKO2	217	VLIMYK	99	Neuron-specific vesicular protein calcyon	Postsynaptic neurotransmitter receptor internalization
Q8IXF0 [https://www.uniprot.org/uniprot/Q8IXF0]	NPAS3	HA-NPAS3-3xFLAG-IRES-mKO2	420 (aa 1-420) bHLH+PAS1+PAS2+PAC domain	VDIVGK	359	Neuronal PAS domain-containing protein 3	Transcription factor involved in neurogenesis
P62995 [https://www.uniprot.org/uniprot/P62995]	TRA2B	HA-TRA2B-3xFLAG-IRES-mKO2	288	VSIVYD	147	Transformer-2 protein homolog beta	Activates the splicing of MAPT/Tau - Implicated in AD and PD
Q8IZD9 [https://www.uniprot.org/uniprot/Q8IZD9]	DOCK3	HA-DOCK3-3xFLAG-IRES-mKO2	420 (aa 1-420) SH3 domain-containing N-terminal	VQILEK	33	Dedicator of cytokinesis protein 3	Presenilin interactor - stimulates Tau/MAPT phosphorylation - NFT accumulation
Q9NSN8 [https://www.uniprot.org/uniprot/Q9NSN8]	SNTG1	HA-SNTG1-3xFLAG-IRES-mKO2	517	QQIVYM	282	Synaptotagmin-1 (Gamma-1-syntrophin)	Synaptic transmission - Presenilin binding & stabilisation - AD biomarker
P21757 [https://www.uniprot.org/uniprot/P21757]	MSR1	HA-MSR1-3xFLAG-IRES-mKO2	451	VQAVHK	396	Macrophage scavenger receptor types I and II	Aβ binding - Microglia activation - Aβ clearance
Q13009 [https://www.uniprot.org/uni	TIAM1	HA-TIAM1-3xFLAG-IRES-mKO2	136 (aa 1261-1397) PH2 domain	VVLVYK	1300	T-lymphoma invasion and metastasis-inducing protein 1	Glutamatergic Synapse Structure and Function in the Hippocampus - Microtubule binding

prot/Q13009]							
P54652 [https://www.uniprot.org/uniprot/P54652]	HSPA2	HA-HSPA2-3xFLAG-IRES-mKO2	500 (aa 1-500) NBD + SBD domain	VQVEYK	104	Heat shock-related 70 kDa protein 2	Molecular chaperone
Q9UPT6 [https://www.uniprot.org/uniprot/Q9UPT6]	MAPK8IP3	HA-MAPK8IP3-3xFLAG-IRES-mKO2	110 (aa 500-610) RH2 domain	VNIHYK	596	C-Jun-amino-terminal kinase-interacting protein 3	Critical regulator of axonal lysosome abundance -MAPK cascade
**Controls**							
P10636-8 [https://www.uniprot.org/uniprot/P10636-8]	MAPT	HA-tau2N4R-3xFLAG-IRES-mKO2	441	VQIVYK	306	Microtubule-associated protein tau	Tau full-length protein
P10636-8 [https://www.uniprot.org/uniprot/P10636-8]	MAPT	HA-tauRD-3xFLAG-IRES-mKO2	128 (244-372) C-terminal Repeat domain	VQIVYK	306	Microtubule-associated protein tau (Repeat Domain)	Tau repeat domain

Supplementary Table 3. Basic information of the patients and disease stage from which the brain extracts were isolated. An informed consent for autopsy and scientific use of autopsy tissue with clinical information was granted from all subjects involved.

Sample ID	Age (years)	Sex	Neuropathological diagnosis	Braak NFT stage	Aβ phase	CERAD score	NIA-AA degree of AD pathology	Post Mortem Interval/hours
A1	71	Female	AD	VI	5	2	high	24
A2	87	Male	AD	VI	5	2	high	12
A3	71	Male	AD	V	5	3	high	12

Supplementary Methods.

Insert DNA sequences.

P14735

GCAGAGGGTCCGCAGGAGTGGGTCTTTCAGGAGTGTAAGGACTTGAATGCCGTTGCATTCCGGTTTAAGGATAAAGAGAGACCCCGAGGTTACACCAGC
AAAATTGCAGGGATTCTTCACTATTACCCACTGGAGGAAGTGCTTACCGCAGAGTACCTGCTGGAAGAGTTCCGCCCAGACTTAATCGAAATGGTTCTGG
ACAAATTGCGTCCCGAGAACGTGCGGGTGGCTATTGTAAGCAAATCTTTTGAGGGGAAAACCGACCGGACAGAAGAGTGGTACGGAACGCAGTATAAG
CAGGAAGCCATTCCTGACGAGGTGATAAAGAAGTGGCAAAACGCCGATTTGAATGGGAAATTCAAGCTGCCTACTAAGAACGAATTTATTCCAACCAATT
TCGAAATACTTCCACTCGAGAAGGAGGCCACTCCATATCCCGCTCTGATTAAGGACACCGCGATGTCAAAGCTGTGGTTTAAACAAGACGATAAATTCTTC
TTGCCTAAGGCATGTCTTAATTTCGAGTTCTTTAGTCCCTTTGCTTATGTGGACCCGCTCCACTGTAATATGGCTTATCTGTACTTGGAGCTTCTGAAGGACT
CCCTCAATGAATATGCCTATGCCGCTGAACTGGCAGGTCTGTCATATGACCTGCAAAACACCATTTACGGGATGTATCTGTCAGTTAAAGGCTACAACGAC
AAGCAGCCAATCCTGCTCAAGAAGATTATAGAGAAAATGGCCACATTTGAAATCGACGAGAAGAGATTTGAAATCATCAAAGAAGCGTATATGAGGTCTT
TGAACAATTTCAGGGCTGAGCAGCCACACCAGCACGCCATGTACTATCTGAGGCTGCTGATGACTGAGGTGGCGTGGACAAAGGATGAGCTCAAAGAGG
GCAGTGGGTCCGATTACAAAGACCATGATGGCGATTATAAGGACCACGACATCGATTATAAAGATGACGATGATAAATAGGTCGAGCCGCGGCAATTCC
GACGTTACTGGCCGAAGCCGCTTGGAATAAGGCCGGTGTGCGTTTGTCTATATGTTATTTTCCACCATATTGCCGTCTTTTGGCAATGTGAGGGCCCGGAA
ACCTGGCCCTGTCTTCTTGACGAGCATTCCTAGGGGTCTTTCCCCTCTCGCCAAAGGAATGCAAGGTCTGTTGAATGTCGTGAAGGAAGCAGTTCCTCTGG
AAGCTTCTTGAAGACAAACAACGTCTGTAGCGACCCTTTGCAGGCAGCGGAACCCCCCACCTGGCAACAGGTGCCTCTGCGGCCAAAAGCCACGTGTATA

AGATACACCTGCAAAGGCGGCACAACCCCAGTGCCACGTTGTGAGTTGGATAGTTGTGGAAAGAGTCAAATGGCTCTCCTCAAGCGTATTCAACAAGGG
GCTGAAGGATGCCCAGAAGGTACCCCATTGTATGGGATCTGATCTGGGGCCTCGGTGCACATGCTTTACATGTGTTTAGTCGAGGTTAAAAAAACGTCTA
GGCCCCCCGAACCACGGGGACGTGGTTTTCCTTTGAAAAACACGATGATAATATGGTGTCAGTGATTAAGCCCGAGATGAAAATGAGATATTATATGGAC
GGGAGCGTCAATGGGCATGAGTTCACTATCGAAGGCGAGGGAACGGGACGCCCTTATGAGGGGCACCAAGAAATGACACTCAGAGTAACTATGGCAGA
AGGCGGTCCAATGCCATTTGCATTCGATCTGGTTAGCCACGTATTTTGCTACGGGCATAGAGTTTTTACAAAATACCCCGAAGAGATCCCAGATTACTTTAA
GCAGGCATTCCCTGAAGGCCTGTCCTGGGAACGATCCCTGGAGTTCGAGGACGGGGGCAGCGCGAGTGTCTCCGCTCACATTAGTCTGAGGGGGAACAC
CTTTTATCATAAATCTAAATTCACAGGAGTCAATTTCCCAGCAGATGGCCCAATTATGCAGAATCAGTCAGTTGATTGGGAGCCTTCTACGGAAAAGATCA
CTGCAAGCGACGGCGTGCTTAAGGGGGATGTCACCATGTATCTGAAACTGGAAGGGGGCGGAAACCATAAGTGTCAAATGAAAACTACCTATAAGGCCG
CAAAGGAGATTCTCGAGATGCCCGGCGATCATTACATCGGTCATCGATTAGTTCGTAAGACCGAGGGAAATATCACTGAACAGGTCGAGGACGCTGTTGC
ACACTCATGAGCTAGCTTGACTGACTGAGATACAGCGTACCTTCAGCTCACAGACATGATAAGATACATTGATGAGTTTGGACAAACCACAACTAGAATGC
AGTGAAAAAAATGCTTTATTTGTGAAATTTGTGATGCTATTGCTTTATTTGTAACCATTATAAGCTGCAATAAACAAGTTAACAACAACAATTGCATTCATTT
TATGTTTCAGGTTCAGGGGGAGGTGTGGGAGGTTTTTTAAAGCAAGTAAAACCTCTACAAATGTGGTATTGGCCCATCTCTATCGGTATCGTAGCATAACC
CCTTGGGGCCTCTAAACGGGTCTTGAGGGGTTTTTTGTGCCCCTCGGGCCGGATTGCTATCTACCGGCATTGGCGCAGAAAAAAATGCCTGATGCGACGC
TGCGCGTCTTATACTCCCACATATGCCAGATTCAGCAACGGATACGGCTTCCCCAACTTGCCCACTTCCATACGTGTCCTCCTTACCAGAAATTTATCCTTAA
GGTCGTCAGCTATCCTGCAGGCGATCTCTCGATTTCGATCAAGACATTCCTTTAATGGTCTTTTCTGGACACCACTAGGGGTCAGAAGTAGTTCATCAAACT
TTCTTCCCTCCCTAATCTCATTGGTTACCTTGGGCTATCGAAACTTAATTAACCAGTCAAGTCAGCTACTTGGCGAGATCGACTTGTCTGGGTTTCGACTACG
CTCAGAATTGCGTCAGTCAAGTTCGATCTGGTCCTTGCTATTGCACCCGTTCTCCGATTACGAGTTTCATTTAAATCATGTGAGCAAAAGGCCAGCAAAAG
GCCAGGAACCGTAAAAAGGCCGCGTTGCTGGCGTTTTTCCATAGGCTCCGCCCCCCTGACGAGCATCACAAAAATCGACGCTCAAGTCAGAGGTGGCGAA
ACCCGACAGGACTATAAAGATACCAGGCGTTTCCCCCTGGAAGCTCCCTCGTGCGCTCTCCTGTTCCGACCCTGCCGCTTACCGGATACCTGTCCGCCTTTC
TCCCTTCGGGAAGCGTGGCGCTTTCTCATAGCTCACGCTGTAGGTATCTCAGTTCGGTGTAGGTCGTTCGCTCCAAGCTGGGCTGTGTGCACGAACCCCCC
GTTCAGCCCGACCGCTGCGCCTTATCCGGTAACTATCGTCTTGAGTCCAACCCGGTAAGACACGACTTATCGCCACTGGCAGCAGCCACTGGTAACAGGAT
TAGCAGAGCGAGGTATGTAGGCGGTGCTACAGAGTTCTTGAAGTGGTGGCCTAACTACGGCTACACTAGAAGAACAGTATTTGGTATCTGCGCTCTGCTG
AAGCCAGTTACCTTCGGAAAAAGAGTTGGTAGCTCTTGATCCGGCAAACAAACCACCGCTGGTAGCGGTGGTTTTTTTGTTTGCAAGCAGCAGATTACGC
GCAGAAAAAAAGGATCTCAAGAAGATCCTTTGATCTTTTCTACGGGGTCTGACGCTCAGTGGAACGAAAACTCACGTTAAGGGATTTTGGTCATGAGATT
ATCAAAAAGGATCTTCACCTAGATCCTTTTAAATTAAAAATGAAGTTTTAAATCAATCTAAAGTATATATGAGTAAACTTGGTCTGACAGTTACCAATGCTT
AATCAGTGAGGCACCTATCTCAGCGATCTGTCTATTTCGTTCATCCATAGTTGCATTTAAATTTCCGAACTCTCCAAGGCCCTCGTCGGAAAATCTTCAAACC
TTTCGTCCGATCCATCTTGCAGGCTACCTCTCGAACGAACTATCGCAAGTCTCTTGGCCGGCCTTGCGCCTTGGCTATTGCTTGGCAGCGCCTATCGCCAGG
TATTACTCCAATCCCGAATATCCGAGATCGGGATCACCCGAGAGAAGTTCAACCTACATCCTCAATCCCGATCTATCCGAGATCCGAGGAATATCGAAATC
GGGGCGCGCCTGGCCTCCGCGCCGGGTTTTGGCGCCTCCCGCGGGCGCCCCCCTCGTCACGGCGAGCGCTGCCACGTCAGACGAAGGGCGCAGGAGCG
TCCTGATCCTTCCGCCCGGACGCTCAGGACAGCGGCCCGCTGCTCATAAGACTCGGCCTTAGAACCCCAGTATCAGCAGAAGGACATTTTAGGACGGGAC
TTGGGTGACTCTAGGGCACTGGTTTTCTTTCCAGAGAGCGGAACAGGCGAGGAAAAGTAGTCCCTTCTCGGCGATTCTGCGGAGGGATCTCCGTGGGGC
GGTGAACGCCGATGATTATATAAGGACGCGCCGGGTGTGGCACAGCTAGTTCCGTCGCAGCCGGGATTTGGGTCGCGGTTCTTGTTTGTGGATCGCTGT
GATCGTCACTTGGTGAGTAGCGGGCTGCTGGGCTGGCCGGGGCTTTCGTGGCCGCCGGGCCGCTCGGTGGGACGGAAGCGTGTGGAGAGACCGCCAAG
GGCTGTAGTCTGGGTCCGCGAGCAAGGTTGCCCTGAACTGGGGGTTGGGGGGAGCGCAGCAAAATGGCGGCTGTTCCCGAGTCTTGAATGGAAGACGC
TTGTGAGGCGGGCTGTGAGGTCGTTGAAACAAGGTGGGGGGCATGGTGGGCGGCAAGAACCCAAGGTCTTGAGCCCTTCGCTAATGCGGGAAAGCTCT
TATTCGGGTGAGATGGGCTGGGCACCATCTGGGGACCCTGACGTGAAGTTTGTCACTGACTGGAGAACTCGGTTTGTCGTCTGTTGCGGGGGCGGCAGT
TATGGCGGTGCCGTTGGGCAGTGCACCCGTACCTTTGGGAGCGCGCGCCCTCGTCGTGTCGTGACGTCACCCGTTCTGTTGGCTTATAATGCAGGGTGGG
GCCACCTGCCGGTAGGTGTGCGGTAGGCTTTTCTCCGTCGCAGGACGCAGGGTTCGGGCCTAGGGTAGGCTCTCCTGAATCGACAGGCGCCGGACCTCT
GGTGAGGGGAGGGATAAGTGAGGCGTCAGTTTCTTTGGTCGGTTTTATGTACCTATCTTCTTAAGTAGCTGAAGCTCCGGTTTTGAACTATGCGCTCGGG
GTTGGCGAGTGTGTTTTGTGAAGTTTTTTAGGCACCTTTTGAAATGTAATCATTTGGGTCAATATGTAATTTTCAGTGTTAGACTTGTAAATTGTCCGCTAA
ATTCTGGCCGTTTTTGGCTTTTTTGTTAGACAACATGACCGAGTACAAGCCCACGGTGCGCCTCGCCACCCGCGACGACGTCCCCAGGGCCGTACGCACCC
TCGCCGCCGCGTTCGCCGACTACCCCGCCACGCGCCACACCGTCGATCCGGACCGCCACATCGAGCGGGTCACCGAGCTGCAAGAACTCTTCCTCACGCG
CGTCGGGCTCGACATCGGCAAGGTGTGGGTCGCGGACGACGGCGCCGCGGTGGCGGTCTGGACCACGCCGGAGAGCGTCGAAGCGGGGGCGGTGTTC
GCCGAGATCGGCCCGCGCATGGCCGAGTTGAGCGGTTCCCGGCTGGCCGCGCAGCAACAGATGGAGGGCCTCCTGGCGCCGCACCGGCCCAAGGAGCC
CGCGTGGTTCCTGGCCACCGTCGGCGTCTCGCCCGACCACCAGGGCAAGGGTCTGGGCAGCGCCGTCGTGCTCCCCGGAGTGGAGGCCGCCGAGCGCGC
CGGGGTGCCCGCCTTCCTGGAGACCTCCGCGCCCCGCAACCTCCCCTTCTACGAGCGGCTCGGCTTCACCGTCACCGCCGACGTCGAGGTGCCCGAAGGA
CCGCGCACCTGGTGCATGACCCGCAAGCCCGGTGCCTAGGCTAGTATGTAAGCCTAGTCTTAGATAATAAAATCGCTATCCATCGAAGATGGATGTGTGT
TGGTTTTTTGTGTGTGTAACGCTAGGCGCGCCTGGTGTACCGAGAACGATCCTCTCAGTGCGAGTCTCGACGATCCATATCGTTGCTTGGCAGTCAGCCAG
TCGGAATCCAGCTTGGGACCCAGGAAGTCCAATCGTCAGATATTGTACTCAAGCCTGGTCACGGCAGCGTACCGATCTGTTTAAACCTAGATATTGATAGT
CTGATCGGTCAACGTATAATCGAGTCCTAGCTTTTGCAAACATCTATCAAGAGACAGGATCAGCAGGAGGCTTTCGCATGAGTATTCAACATTTCCGTGTC
GCCCTTATTCCCTTTTTTGCGGCATTTTGCCTTCCTGTTTTTGCTCACCCAGAAACGCTGGTGAAAGTAAAAGATGCTGAAGATCAGTTGGGTGCGCGAGTG
GGTTACATCGAACTGGATCTCAACAGCGGTAAGATCCTTGAGAGTTTTCGCCCCGAAGAACGCTTTCCAATGATGAGCACTTTTAAAGTTCTGCTATGTGG
CGCGGTATTATCCCGTATTGACGCCGGGCAAGAGCAACTCGGTCGCCGCATACACTATTCTCAGAATGACTTGGTTGAGTATTCACCAGTCACAGAAAAGC
ATCTTACGGATGGCATGACAGTAAGAGAATTATGCAGTGCTGCCATAACCATGAGTGATAACACTGCGGCCAACTTACTTCTGACAACGATTGGAGGACC
GAAGGAGCTAACCGCTTTTTTGCACAACATGGGGGATCATGTAACTCGCCTTGATCGTTGGGAACCGGAGCTGAATGAAGCCATACCAAACGACGAGCGT
GACACCACGATGCCTGTAGCAATGGCAACAACCTTGCGTAAACTATTAACTGGCGAACTACTTACTCTAGCTTCCCGGCAACAGTTGATAGACTGGATGGA
GGCGGATAAAGTTGCAGGACCACTTCTGCGCTCGGCCCTTCCGGCTGGCTGGTTTATTGCTGATAAATCTGGAGCCGGTGAGCGTGGGTCTCGCGGTATC
ATTGCAGCACTGGGGCCAGATGGTAAGCCCTCCCGTATCGTAGTTATCTACACGACGGGGAGTCAGGCAACTATGGATGAACGAAATAGACAGATCGCT
GAGATAGGTGCCTCACTGATTAAGCATTGGTAACCGATTCTAGGTGCATTGGCGCAGAAAAAAATGCCTGATGCGACGCTGCGCGTCTTATACTCCCACAT
ATGCCAGATTCAGCAACGGATACGGCTTCCCCAACTTGCCCACTTCCATACGTGTCCTCCTTACCAGAAATTTATCCTTAAGATCGTTTAAACTCGACTCTGG
CTCTATCGAATCTCCGTCGTTTCGAGCTTACGCGAACAGCCGTGGCGCTCATTTGCTCGTCGGGCATCGAATCTCGTCAGCTATCGTCAGCTTACCTTTTTG
GCAGCGATCGCGGCTCCCGACATCTTGGACCATTAGCTCCACAGGTATCTTCTTCCCTCTAGTGGTCATAACAGCAGCTTCAGCTACCTCTCAATTCAAAAA
ACCCCTCAAGACCCGTTTAGAGGCCCCAAGGGGTTATGCTATCAATCGTTGCGTTACACACACAAAAAACCAACACACATCCATCTTCGATGGATAGCGAT
TTTATTATCTAACTGCTGATCGAGTGTAGCCAGATCTAGTAATCAATTACGGGGTCATTAGTTCATAGCCCATATATGGAGTTCCGCGTTACATAACTTACG
GTAAATGGCCCGCCTGGCTGACCGCCCAACGACCCCCGCCCATTGACGTCAATAATGACGTATGTTCCCATAGTAACGCCAATAGGGACTTTCCATTGACG
TCAATGGGTGGAGTATTTACGGTAAACTGCCCACTTGGCAGTACATCAAGTGTATCATATGCCAAGTACGCCCCCTATTGACGTCAATGACGGTAAATGGC
CCGCCTGGCATTATGCCCAGTACATGACCTTATGGGACTTTCCTACTTGGCAGTACATCTACGTATTAGTCATCGCTATTACCATGCTGATGCGGTTTTGGC
AGTACATCAATGGGCGTGGATAGCGGTTTGACTCACGGGGATTTCCAAGTCTCCACCCCATTGACGTCAATGGGAGTTTGTTTTGGCACCAAAATCAACG
GGACTTTCCAAAATGTCGTAACAACTCCGCCCCATTGACGCAAATGGGCGGTAGGCGTGTACGGTGGGAGGTCTATATAAGCAGAGCTGGTTTAGTGAAC
CGTCAGATCAGATCTTTGTCGATCCTACCATCCACTCGACACACCCGCCAGCGGCCGCATGTACCCGTACGATGTCCCCGACTACGCCGGATCAGGCTCT

Q15042

GCTGCAGACTCCGAACCTGAGTCAGAGGTGTTTGAGATCACGGACTTCACCACAGCCTCTGAATGGGAACGCTTCATTTCCAAGGTCGAGGAAGTTTTGA
ACGATTGGAAACTCATTGGAAATTCCCTGGGGAAACCGTTAGAGAAAGGGATCTTTACCTCTGGCACTTGGGAAGAGAAATCCGACGAGATTTCCTTTGC
AGATTTTAAGTTTTCCGTGACTCATCACTACCTGGTGCAGGAGTCCACAGATAAGGAGGGCAAGGACGAACTGCTCGAGGACGTAGTCCCACAAAGCATG
CAGGATCTCCTTGGGATGAACAACGACTTCCCGCCAAGGGCGCACTGCCTGGTTCGCTGGTATGGTCTCCGAGAGTTCGTGGTAATTGCACCAGCCGCAC
ATTCAGACGCCGTGCTGAGCGAGTCTAAGTGTAATCTTTTGCTCTCTTCTGTGTCTATTGCTCTGGGGAACACTGGTTGCCAGGTTCCCCTGTTTGTGCAGA
TCCACCATAAATGGCGACGGATGTACGTGGGCGAATGCCAGGGTCCTGGGGTGCGGACAGACTTCGAAATGGTACACCTGCGCAAGGTGCCAAACCAGT
ACACTCATCTGTCAGGCCTGCTGGATATTTTCAAGAGCAAAATCGGGTGCCCTTTAACTCCCTTACCTCCCGTGTCAATTGCTATTCGGTTCACTTACGTCCT
TCAGGATTGGCAGCAGTACTTTTGGCCCCAGCAGCCTCCAGACATTGACGCTCTTGTGGGCGGGGAGGTTGGCGGACTGGAGTTCGGCAAACTGCCTTTC
GGGGCCTGCGAAGATCCCATAAGTGAACTGCACTTGGCAACCACCTGGCCCCACCTGACGGAGGGCATAATAGTGGATAACGACGTGTATTCCGATCTG
GATCCTATTCAAGCCCCTCACTGGAGCGTCCGGGTGAGGAAGGCCGAAAATCCTCAGTGCCTTCTGGGCGACTTCGTGACCGAATTCTTCAAAATTTGCCG
GCGAAAGGAGTCCACTGACGAGATCCTCGGACGAAGTGCCTTCGAAGAAGAAGGGAAAGAGACAGCCGACATTACTCATGCCCTTTCTAAGCTGACAGA
ACCCGCTAGTGTTCCTATTCATAAACTGAGCGTCAGTAACATGGTGCACACCGCCAAGAAAAAGATCCGGAAGCATAGGGGCGTCGAGGAGTCCGGCAG
TGGGTCCGATTACAAAGACCATGATGGCGATTATAAGGACCACGACATCGATTATAAAGATGACGATGATAAATAGGTCGAGCCGCGGCAATTCCGACG
TTACTGGCCGAAGCCGCTTGGAATAAGGCCGGTGTGCGTTTGTCTATATGTTATTTTCCACCATATTGCCGTCTTTTGGCAATGTGAGGGCCCGGAAACCT
GGCCCTGTCTTCTTGACGAGCATTCCTAGGGGTCTTTCCCCTCTCGCCAAAGGAATGCAAGGTCTGTTGAATGTCGTGAAGGAAGCAGTTCCTCTGGAAGC
TTCTTGAAGACAAACAACGTCTGTAGCGACCCTTTGCAGGCAGCGGAACCCCCCACCTGGCAACAGGTGCCTCTGCGGCCAAAAGCCACGTGTATAAGAT
ACACCTGCAAAGGCGGCACAACCCCAGTGCCACGTTGTGAGTTGGATAGTTGTGGAAAGAGTCAAATGGCTCTCCTCAAGCGTATTCAACAAGGGGCTG
AAGGATGCCCAGAAGGTACCCCATTGTATGGGATCTGATCTGGGGCCTCGGTGCACATGCTTTACATGTGTTTAGTCGAGGTTAAAAAAACGTCTAGGCC
CCCCGAACCACGGGGACGTGGTTTTCCTTTGAAAAACACGATGATAATATGGTGTCAGTGATTAAGCCCGAGATGAAAATGAGATATTATATGGACGGGA
GCGTCAATGGGCATGAGTTCACTATCGAAGGCGAGGGAACGGGACGCCCTTATGAGGGGCACCAAGAAATGACACTCAGAGTAACTATGGCAGAAGGC
GGTCCAATGCCATTTGCATTCGATCTGGTTAGCCACGTATTTTGCTACGGGCATAGAGTTTTTACAAAATACCCCGAAGAGATCCCAGATTACTTTAAGCAG
GCATTCCCTGAAGGCCTGTCCTGGGAACGATCCCTGGAGTTCGAGGACGGGGGCAGCGCGAGTGTCTCCGCTCACATTAGTCTGAGGGGGAACACCTTTT
ATCATAAATCTAAATTCACAGGAGTCAATTTCCCAGCAGATGGCCCAATTATGCAGAATCAGTCAGTTGATTGGGAGCCTTCTACGGAAAAGATCACTGCA
AGCGACGGCGTGCTTAAGGGGGATGTCACCATGTATCTGAAACTGGAAGGGGGCGGAAACCATAAGTGTCAAATGAAAACTACCTATAAGGCCGCAAA
GGAGATTCTCGAGATGCCCGGCGATCATTACATCGGTCATCGATTAGTTCGTAAGACCGAGGGAAATATCACTGAACAGGTCGAGGACGCTGTTGCACAC
TCATGAGCTAGCTTGACTGACTGAGATACAGCGTACCTTCAGCTCACAGACATGATAAGATACATTGATGAGTTTGGACAAACCACAACTAGAATGCAGT
GAAAAAAATGCTTTATTTGTGAAATTTGTGATGCTATTGCTTTATTTGTAACCATTATAAGCTGCAATAAACAAGTTAACAACAACAATTGCATTCATTTTAT
GTTTCAGGTTCAGGGGGAGGTGTGGGAGGTTTTTTAAAGCAAGTAAAACCTCTACAAATGTGGTATTGGCCCATCTCTATCGGTATCGTAGCATAACCCCT
TGGGGCCTCTAAACGGGTCTTGAGGGGTTTTTTGTGCCCCTCGGGCCGGATTGCTATCTACCGGCATTGGCGCAGAAAAAAATGCCTGATGCGACGCTGC
GCGTCTTATACTCCCACATATGCCAGATTCAGCAACGGATACGGCTTCCCCAACTTGCCCACTTCCATACGTGTCCTCCTTACCAGAAATTTATCCTTAAGGT
CGTCAGCTATCCTGCAGGCGATCTCTCGATTTCGATCAAGACATTCCTTTAATGGTCTTTTCTGGACACCACTAGGGGTCAGAAGTAGTTCATCAAACTTTC
TTCCCTCCCTAATCTCATTGGTTACCTTGGGCTATCGAAACTTAATTAACCAGTCAAGTCAGCTACTTGGCGAGATCGACTTGTCTGGGTTTCGACTACGCTC
AGAATTGCGTCAGTCAAGTTCGATCTGGTCCTTGCTATTGCACCCGTTCTCCGATTACGAGTTTCATTTAAATCATGTGAGCAAAAGGCCAGCAAAAGGCC
AGGAACCGTAAAAAGGCCGCGTTGCTGGCGTTTTTCCATAGGCTCCGCCCCCCTGACGAGCATCACAAAAATCGACGCTCAAGTCAGAGGTGGCGAAACC
CGACAGGACTATAAAGATACCAGGCGTTTCCCCCTGGAAGCTCCCTCGTGCGCTCTCCTGTTCCGACCCTGCCGCTTACCGGATACCTGTCCGCCTTTCTCC
CTTCGGGAAGCGTGGCGCTTTCTCATAGCTCACGCTGTAGGTATCTCAGTTCGGTGTAGGTCGTTCGCTCCAAGCTGGGCTGTGTGCACGAACCCCCCGTT
CAGCCCGACCGCTGCGCCTTATCCGGTAACTATCGTCTTGAGTCCAACCCGGTAAGACACGACTTATCGCCACTGGCAGCAGCCACTGGTAACAGGATTAG
CAGAGCGAGGTATGTAGGCGGTGCTACAGAGTTCTTGAAGTGGTGGCCTAACTACGGCTACACTAGAAGAACAGTATTTGGTATCTGCGCTCTGCTGAAG
CCAGTTACCTTCGGAAAAAGAGTTGGTAGCTCTTGATCCGGCAAACAAACCACCGCTGGTAGCGGTGGTTTTTTTGTTTGCAAGCAGCAGATTACGCGCA
GAAAAAAAGGATCTCAAGAAGATCCTTTGATCTTTTCTACGGGGTCTGACGCTCAGTGGAACGAAAACTCACGTTAAGGGATTTTGGTCATGAGATTATCA
AAAAGGATCTTCACCTAGATCCTTTTAAATTAAAAATGAAGTTTTAAATCAATCTAAAGTATATATGAGTAAACTTGGTCTGACAGTTACCAATGCTTAATC
AGTGAGGCACCTATCTCAGCGATCTGTCTATTTCGTTCATCCATAGTTGCATTTAAATTTCCGAACTCTCCAAGGCCCTCGTCGGAAAATCTTCAAACCTTTC
GTCCGATCCATCTTGCAGGCTACCTCTCGAACGAACTATCGCAAGTCTCTTGGCCGGCCTTGCGCCTTGGCTATTGCTTGGCAGCGCCTATCGCCAGGTATT
ACTCCAATCCCGAATATCCGAGATCGGGATCACCCGAGAGAAGTTCAACCTACATCCTCAATCCCGATCTATCCGAGATCCGAGGAATATCGAAATCGGG
GCGCGCCTGGCCTCCGCGCCGGGTTTTGGCGCCTCCCGCGGGCGCCCCCCTCGTCACGGCGAGCGCTGCCACGTCAGACGAAGGGCGCAGGAGCGTCCT
GATCCTTCCGCCCGGACGCTCAGGACAGCGGCCCGCTGCTCATAAGACTCGGCCTTAGAACCCCAGTATCAGCAGAAGGACATTTTAGGACGGGACTTGG
GTGACTCTAGGGCACTGGTTTTCTTTCCAGAGAGCGGAACAGGCGAGGAAAAGTAGTCCCTTCTCGGCGATTCTGCGGAGGGATCTCCGTGGGGCGGTG
AACGCCGATGATTATATAAGGACGCGCCGGGTGTGGCACAGCTAGTTCCGTCGCAGCCGGGATTTGGGTCGCGGTTCTTGTTTGTGGATCGCTGTGATCG
TCACTTGGTGAGTAGCGGGCTGCTGGGCTGGCCGGGGCTTTCGTGGCCGCCGGGCCGCTCGGTGGGACGGAAGCGTGTGGAGAGACCGCCAAGGGCTG
TAGTCTGGGTCCGCGAGCAAGGTTGCCCTGAACTGGGGGTTGGGGGGAGCGCAGCAAAATGGCGGCTGTTCCCGAGTCTTGAATGGAAGACGCTTGTG
AGGCGGGCTGTGAGGTCGTTGAAACAAGGTGGGGGGCATGGTGGGCGGCAAGAACCCAAGGTCTTGAGCCCTTCGCTAATGCGGGAAAGCTCTTATTC
GGGTGAGATGGGCTGGGCACCATCTGGGGACCCTGACGTGAAGTTTGTCACTGACTGGAGAACTCGGTTTGTCGTCTGTTGCGGGGGCGGCAGTTATGG
CGGTGCCGTTGGGCAGTGCACCCGTACCTTTGGGAGCGCGCGCCCTCGTCGTGTCGTGACGTCACCCGTTCTGTTGGCTTATAATGCAGGGTGGGGCCAC
CTGCCGGTAGGTGTGCGGTAGGCTTTTCTCCGTCGCAGGACGCAGGGTTCGGGCCTAGGGTAGGCTCTCCTGAATCGACAGGCGCCGGACCTCTGGTGA
GGGGAGGGATAAGTGAGGCGTCAGTTTCTTTGGTCGGTTTTATGTACCTATCTTCTTAAGTAGCTGAAGCTCCGGTTTTGAACTATGCGCTCGGGGTTGGC
GAGTGTGTTTTGTGAAGTTTTTTAGGCACCTTTTGAAATGTAATCATTTGGGTCAATATGTAATTTTCAGTGTTAGACTTGTAAATTGTCCGCTAAATTCTGG
CCGTTTTTGGCTTTTTTGTTAGACAACATGACCGAGTACAAGCCCACGGTGCGCCTCGCCACCCGCGACGACGTCCCCAGGGCCGTACGCACCCTCGCCGC
CGCGTTCGCCGACTACCCCGCCACGCGCCACACCGTCGATCCGGACCGCCACATCGAGCGGGTCACCGAGCTGCAAGAACTCTTCCTCACGCGCGTCGGG
CTCGACATCGGCAAGGTGTGGGTCGCGGACGACGGCGCCGCGGTGGCGGTCTGGACCACGCCGGAGAGCGTCGAAGCGGGGGCGGTGTTCGCCGAGA
TCGGCCCGCGCATGGCCGAGTTGAGCGGTTCCCGGCTGGCCGCGCAGCAACAGATGGAGGGCCTCCTGGCGCCGCACCGGCCCAAGGAGCCCGCGTGG
TTCCTGGCCACCGTCGGCGTCTCGCCCGACCACCAGGGCAAGGGTCTGGGCAGCGCCGTCGTGCTCCCCGGAGTGGAGGCCGCCGAGCGCGCCGGGGT
GCCCGCCTTCCTGGAGACCTCCGCGCCCCGCAACCTCCCCTTCTACGAGCGGCTCGGCTTCACCGTCACCGCCGACGTCGAGGTGCCCGAAGGACCGCGC
ACCTGGTGCATGACCCGCAAGCCCGGTGCCTAGGCTAGTATGTAAGCCTAGTCTTAGATAATAAAATCGCTATCCATCGAAGATGGATGTGTGTTGGTTTT
TTGTGTGTGTAACGCTAGGCGCGCCTGGTGTACCGAGAACGATCCTCTCAGTGCGAGTCTCGACGATCCATATCGTTGCTTGGCAGTCAGCCAGTCGGAA
TCCAGCTTGGGACCCAGGAAGTCCAATCGTCAGATATTGTACTCAAGCCTGGTCACGGCAGCGTACCGATCTGTTTAAACCTAGATATTGATAGTCTGATC
GGTCAACGTATAATCGAGTCCTAGCTTTTGCAAACATCTATCAAGAGACAGGATCAGCAGGAGGCTTTCGCATGAGTATTCAACATTTCCGTGTCGCCCTT
ATTCCCTTTTTTGCGGCATTTTGCCTTCCTGTTTTTGCTCACCCAGAAACGCTGGTGAAAGTAAAAGATGCTGAAGATCAGTTGGGTGCGCGAGTGGGTTA
CATCGAACTGGATCTCAACAGCGGTAAGATCCTTGAGAGTTTTCGCCCCGAAGAACGCTTTCCAATGATGAGCACTTTTAAAGTTCTGCTATGTGGCGCGG

TATTATCCCGTATTGACGCCGGGCAAGAGCAACTCGGTCGCCGCATACACTATTCTCAGAATGACTTGGTTGAGTATTCACCAGTCACAGAAAAGCATCTT
ACGGATGGCATGACAGTAAGAGAATTATGCAGTGCTGCCATAACCATGAGTGATAACACTGCGGCCAACTTACTTCTGACAACGATTGGAGGACCGAAG
GAGCTAACCGCTTTTTTGCACAACATGGGGGATCATGTAACTCGCCTTGATCGTTGGGAACCGGAGCTGAATGAAGCCATACCAAACGACGAGCGTGACA
CCACGATGCCTGTAGCAATGGCAACAACCTTGCGTAAACTATTAACTGGCGAACTACTTACTCTAGCTTCCCGGCAACAGTTGATAGACTGGATGGAGGC
GGATAAAGTTGCAGGACCACTTCTGCGCTCGGCCCTTCCGGCTGGCTGGTTTATTGCTGATAAATCTGGAGCCGGTGAGCGTGGGTCTCGCGGTATCATT
GCAGCACTGGGGCCAGATGGTAAGCCCTCCCGTATCGTAGTTATCTACACGACGGGGAGTCAGGCAACTATGGATGAACGAAATAGACAGATCGCTGAG
ATAGGTGCCTCACTGATTAAGCATTGGTAACCGATTCTAGGTGCATTGGCGCAGAAAAAAATGCCTGATGCGACGCTGCGCGTCTTATACTCCCACATATG
CCAGATTCAGCAACGGATACGGCTTCCCCAACTTGCCCACTTCCATACGTGTCCTCCTTACCAGAAATTTATCCTTAAGATCGTTTAAACTCGACTCTGGCTC
TATCGAATCTCCGTCGTTTCGAGCTTACGCGAACAGCCGTGGCGCTCATTTGCTCGTCGGGCATCGAATCTCGTCAGCTATCGTCAGCTTACCTTTTTGGCA
GCGATCGCGGCTCCCGACATCTTGGACCATTAGCTCCACAGGTATCTTCTTCCCTCTAGTGGTCATAACAGCAGCTTCAGCTACCTCTCAATTCAAAAAACC
CCTCAAGACCCGTTTAGAGGCCCCAAGGGGTTATGCTATCAATCGTTGCGTTACACACACAAAAAACCAACACACATCCATCTTCGATGGATAGCGATTTT
ATTATCTAACTGCTGATCGAGTGTAGCCAGATCTAGTAATCAATTACGGGGTCATTAGTTCATAGCCCATATATGGAGTTCCGCGTTACATAACTTACGGTA
AATGGCCCGCCTGGCTGACCGCCCAACGACCCCCGCCCATTGACGTCAATAATGACGTATGTTCCCATAGTAACGCCAATAGGGACTTTCCATTGACGTCA
ATGGGTGGAGTATTTACGGTAAACTGCCCACTTGGCAGTACATCAAGTGTATCATATGCCAAGTACGCCCCCTATTGACGTCAATGACGGTAAATGGCCCG
CCTGGCATTATGCCCAGTACATGACCTTATGGGACTTTCCTACTTGGCAGTACATCTACGTATTAGTCATCGCTATTACCATGCTGATGCGGTTTTGGCAGT
ACATCAATGGGCGTGGATAGCGGTTTGACTCACGGGGATTTCCAAGTCTCCACCCCATTGACGTCAATGGGAGTTTGTTTTGGCACCAAAATCAACGGGA
CTTTCCAAAATGTCGTAACAACTCCGCCCCATTGACGCAAATGGGCGGTAGGCGTGTACGGTGGGAGGTCTATATAAGCAGAGCTGGTTTAGTGAACCGT
CAGATCAGATCTTTGTCGATCCTACCATCCACTCGACACACCCGCCAGCGGCCGCATGTACCCGTACGATGTCCCCGACTACGCCGGATCAGGCTCT

Q13009

GATTTGAGCATGGGAGACCTTTTACTGCACACCACAGTCATTTGGCTGAATCCACCGGCCAGCCTCGGGAAGTGGAAGAAGGAACCCGAGCTGGCTGCCT
TTGTCTTCAAGACAGCTGTTGTACTGGTGTACAAAGACGGGTCTAAGCAGAAGAAAAAGTTGGTGGGGAGCCATCGCCTGAGTATCTATGAGGACTGGG
ACCCATTTAGGTTCCGACACATGATCCCCACAGAAGCCCTGCAGGTGCGTGCCTTAGCGAGTGCTGATGCTGAGGCCAACGCCGTGTGCGAGATTGTGCA
TGTGAAGAGCGAAAGCGAAGGGCGGCCCGAACGGGTGTTTCATCTCTGTTGCTCCTCCCCTGAAAGTAGAAAGGACTTCCTGAAAGCCGTCCACTCTATT
CTGCGGGACAAGGGCAGTGGGTCCGATTACAAAGACCATGATGGCGATTATAAGGACCACGACATCGATTATAAAGATGACGATGATAAATAGGTCGAG
CCGCGGCAATTCCGACGTTACTGGCCGAAGCCGCTTGGAATAAGGCCGGTGTGCGTTTGTCTATATGTTATTTTCCACCATATTGCCGTCTTTTGGCAATGT
GAGGGCCCGGAAACCTGGCCCTGTCTTCTTGACGAGCATTCCTAGGGGTCTTTCCCCTCTCGCCAAAGGAATGCAAGGTCTGTTGAATGTCGTGAAGGAA
GCAGTTCCTCTGGAAGCTTCTTGAAGACAAACAACGTCTGTAGCGACCCTTTGCAGGCAGCGGAACCCCCCACCTGGCAACAGGTGCCTCTGCGGCCAAA
AGCCACGTGTATAAGATACACCTGCAAAGGCGGCACAACCCCAGTGCCACGTTGTGAGTTGGATAGTTGTGGAAAGAGTCAAATGGCTCTCCTCAAGCGT
ATTCAACAAGGGGCTGAAGGATGCCCAGAAGGTACCCCATTGTATGGGATCTGATCTGGGGCCTCGGTGCACATGCTTTACATGTGTTTAGTCGAGGTTA
AAAAAACGTCTAGGCCCCCCGAACCACGGGGACGTGGTTTTCCTTTGAAAAACACGATGATAATATGGTGTCAGTGATTAAGCCCGAGATGAAAATGAGA
TATTATATGGACGGGAGCGTCAATGGGCATGAGTTCACTATCGAAGGCGAGGGAACGGGACGCCCTTATGAGGGGCACCAAGAAATGACACTCAGAGT
AACTATGGCAGAAGGCGGTCCAATGCCATTTGCATTCGATCTGGTTAGCCACGTATTTTGCTACGGGCATAGAGTTTTTACAAAATACCCCGAAGAGATCC
CAGATTACTTTAAGCAGGCATTCCCTGAAGGCCTGTCCTGGGAACGATCCCTGGAGTTCGAGGACGGGGGCAGCGCGAGTGTCTCCGCTCACATTAGTCT
GAGGGGGAACACCTTTTATCATAAATCTAAATTCACAGGAGTCAATTTCCCAGCAGATGGCCCAATTATGCAGAATCAGTCAGTTGATTGGGAGCCTTCTA
CGGAAAAGATCACTGCAAGCGACGGCGTGCTTAAGGGGGATGTCACCATGTATCTGAAACTGGAAGGGGGCGGAAACCATAAGTGTCAAATGAAAACT
ACCTATAAGGCCGCAAAGGAGATTCTCGAGATGCCCGGCGATCATTACATCGGTCATCGATTAGTTCGTAAGACCGAGGGAAATATCACTGAACAGGTCG
AGGACGCTGTTGCACACTCATGAGCTAGCTTGACTGACTGAGATACAGCGTACCTTCAGCTCACAGACATGATAAGATACATTGATGAGTTTGGACAAAC
CACAACTAGAATGCAGTGAAAAAAATGCTTTATTTGTGAAATTTGTGATGCTATTGCTTTATTTGTAACCATTATAAGCTGCAATAAACAAGTTAACAACAA
CAATTGCATTCATTTTATGTTTCAGGTTCAGGGGGAGGTGTGGGAGGTTTTTTAAAGCAAGTAAAACCTCTACAAATGTGGTATTGGCCCATCTCTATCGG
TATCGTAGCATAACCCCTTGGGGCCTCTAAACGGGTCTTGAGGGGTTTTTTGTGCCCCTCGGGCCGGATTGCTATCTACCGGCATTGGCGCAGAAAAAAAT
GCCTGATGCGACGCTGCGCGTCTTATACTCCCACATATGCCAGATTCAGCAACGGATACGGCTTCCCCAACTTGCCCACTTCCATACGTGTCCTCCTTACCA
GAAATTTATCCTTAAGGTCGTCAGCTATCCTGCAGGCGATCTCTCGATTTCGATCAAGACATTCCTTTAATGGTCTTTTCTGGACACCACTAGGGGTCAGAA
GTAGTTCATCAAACTTTCTTCCCTCCCTAATCTCATTGGTTACCTTGGGCTATCGAAACTTAATTAACCAGTCAAGTCAGCTACTTGGCGAGATCGACTTGTC
TGGGTTTCGACTACGCTCAGAATTGCGTCAGTCAAGTTCGATCTGGTCCTTGCTATTGCACCCGTTCTCCGATTACGAGTTTCATTTAAATCATGTGAGCAA
AAGGCCAGCAAAAGGCCAGGAACCGTAAAAAGGCCGCGTTGCTGGCGTTTTTCCATAGGCTCCGCCCCCCTGACGAGCATCACAAAAATCGACGCTCAA
GTCAGAGGTGGCGAAACCCGACAGGACTATAAAGATACCAGGCGTTTCCCCCTGGAAGCTCCCTCGTGCGCTCTCCTGTTCCGACCCTGCCGCTTACCGG
ATACCTGTCCGCCTTTCTCCCTTCGGGAAGCGTGGCGCTTTCTCATAGCTCACGCTGTAGGTATCTCAGTTCGGTGTAGGTCGTTCGCTCCAAGCTGGGCTG
TGTGCACGAACCCCCCGTTCAGCCCGACCGCTGCGCCTTATCCGGTAACTATCGTCTTGAGTCCAACCCGGTAAGACACGACTTATCGCCACTGGCAGCAG
CCACTGGTAACAGGATTAGCAGAGCGAGGTATGTAGGCGGTGCTACAGAGTTCTTGAAGTGGTGGCCTAACTACGGCTACACTAGAAGAACAGTATTTG
GTATCTGCGCTCTGCTGAAGCCAGTTACCTTCGGAAAAAGAGTTGGTAGCTCTTGATCCGGCAAACAAACCACCGCTGGTAGCGGTGGTTTTTTTGTTTGC
AAGCAGCAGATTACGCGCAGAAAAAAAGGATCTCAAGAAGATCCTTTGATCTTTTCTACGGGGTCTGACGCTCAGTGGAACGAAAACTCACGTTAAGGGA
TTTTGGTCATGAGATTATCAAAAAGGATCTTCACCTAGATCCTTTTAAATTAAAAATGAAGTTTTAAATCAATCTAAAGTATATATGAGTAAACTTGGTCTG
ACAGTTACCAATGCTTAATCAGTGAGGCACCTATCTCAGCGATCTGTCTATTTCGTTCATCCATAGTTGCATTTAAATTTCCGAACTCTCCAAGGCCCTCGTC
GGAAAATCTTCAAACCTTTCGTCCGATCCATCTTGCAGGCTACCTCTCGAACGAACTATCGCAAGTCTCTTGGCCGGCCTTGCGCCTTGGCTATTGCTTGGC
AGCGCCTATCGCCAGGTATTACTCCAATCCCGAATATCCGAGATCGGGATCACCCGAGAGAAGTTCAACCTACATCCTCAATCCCGATCTATCCGAGATCC
GAGGAATATCGAAATCGGGGCGCGCCTGGCCTCCGCGCCGGGTTTTGGCGCCTCCCGCGGGCGCCCCCCTCGTCACGGCGAGCGCTGCCACGTCAGACG
AAGGGCGCAGGAGCGTCCTGATCCTTCCGCCCGGACGCTCAGGACAGCGGCCCGCTGCTCATAAGACTCGGCCTTAGAACCCCAGTATCAGCAGAAGGA
CATTTTAGGACGGGACTTGGGTGACTCTAGGGCACTGGTTTTCTTTCCAGAGAGCGGAACAGGCGAGGAAAAGTAGTCCCTTCTCGGCGATTCTGCGGAG
GGATCTCCGTGGGGCGGTGAACGCCGATGATTATATAAGGACGCGCCGGGTGTGGCACAGCTAGTTCCGTCGCAGCCGGGATTTGGGTCGCGGTTCTTG
TTTGTGGATCGCTGTGATCGTCACTTGGTGAGTAGCGGGCTGCTGGGCTGGCCGGGGCTTTCGTGGCCGCCGGGCCGCTCGGTGGGACGGAAGCGTGTG
GAGAGACCGCCAAGGGCTGTAGTCTGGGTCCGCGAGCAAGGTTGCCCTGAACTGGGGGTTGGGGGGAGCGCAGCAAAATGGCGGCTGTTCCCGAGTCT
TGAATGGAAGACGCTTGTGAGGCGGGCTGTGAGGTCGTTGAAACAAGGTGGGGGGCATGGTGGGCGGCAAGAACCCAAGGTCTTGAGCCCTTCGCTAA
TGCGGGAAAGCTCTTATTCGGGTGAGATGGGCTGGGCACCATCTGGGGACCCTGACGTGAAGTTTGTCACTGACTGGAGAACTCGGTTTGTCGTCTGTTG
CGGGGGCGGCAGTTATGGCGGTGCCGTTGGGCAGTGCACCCGTACCTTTGGGAGCGCGCGCCCTCGTCGTGTCGTGACGTCACCCGTTCTGTTGGCTTAT
AATGCAGGGTGGGGCCACCTGCCGGTAGGTGTGCGGTAGGCTTTTCTCCGTCGCAGGACGCAGGGTTCGGGCCTAGGGTAGGCTCTCCTGAATCGACAG
GCGCCGGACCTCTGGTGAGGGGAGGGATAAGTGAGGCGTCAGTTTCTTTGGTCGGTTTTATGTACCTATCTTCTTAAGTAGCTGAAGCTCCGGTTTTGAAC
TATGCGCTCGGGGTTGGCGAGTGTGTTTTGTGAAGTTTTTTAGGCACCTTTTGAAATGTAATCATTTGGGTCAATATGTAATTTTCAGTGTTAGACTTGTAA
ATTGTCCGCTAAATTCTGGCCGTTTTTGGCTTTTTTGTTAGACAACATGACCGAGTACAAGCCCACGGTGCGCCTCGCCACCCGCGACGACGTCCCCAGGG

CCGTACGCACCCTCGCCGCCGCGTTCGCCGACTACCCCGCCACGCGCCACACCGTCGATCCGGACCGCCACATCGAGCGGGTCACCGAGCTGCAAGAACT
CTTCCTCACGCGCGTCGGGCTCGACATCGGCAAGGTGTGGGTCGCGGACGACGGCGCCGCGGTGGCGGTCTGGACCACGCCGGAGAGCGTCGAAGCGG
GGGCGGTGTTCGCCGAGATCGGCCCGCGCATGGCCGAGTTGAGCGGTTCCCGGCTGGCCGCGCAGCAACAGATGGAGGGCCTCCTGGCGCCGCACCGG
CCCAAGGAGCCCGCGTGGTTCCTGGCCACCGTCGGCGTCTCGCCCGACCACCAGGGCAAGGGTCTGGGCAGCGCCGTCGTGCTCCCCGGAGTGGAGGCC
GCCGAGCGCGCCGGGGTGCCCGCCTTCCTGGAGACCTCCGCGCCCCGCAACCTCCCCTTCTACGAGCGGCTCGGCTTCACCGTCACCGCCGACGTCGAGG
TGCCCGAAGGACCGCGCACCTGGTGCATGACCCGCAAGCCCGGTGCCTAGGCTAGTATGTAAGCCTAGTCTTAGATAATAAAATCGCTATCCATCGAAGA
TGGATGTGTGTTGGTTTTTTGTGTGTGTAACGCTAGGCGCGCCTGGTGTACCGAGAACGATCCTCTCAGTGCGAGTCTCGACGATCCATATCGTTGCTTGG
CAGTCAGCCAGTCGGAATCCAGCTTGGGACCCAGGAAGTCCAATCGTCAGATATTGTACTCAAGCCTGGTCACGGCAGCGTACCGATCTGTTTAAACCTA
GATATTGATAGTCTGATCGGTCAACGTATAATCGAGTCCTAGCTTTTGCAAACATCTATCAAGAGACAGGATCAGCAGGAGGCTTTCGCATGAGTATTCAA
CATTTCCGTGTCGCCCTTATTCCCTTTTTTGCGGCATTTTGCCTTCCTGTTTTTGCTCACCCAGAAACGCTGGTGAAAGTAAAAGATGCTGAAGATCAGTTGG
GTGCGCGAGTGGGTTACATCGAACTGGATCTCAACAGCGGTAAGATCCTTGAGAGTTTTCGCCCCGAAGAACGCTTTCCAATGATGAGCACTTTTAAAGTT
CTGCTATGTGGCGCGGTATTATCCCGTATTGACGCCGGGCAAGAGCAACTCGGTCGCCGCATACACTATTCTCAGAATGACTTGGTTGAGTATTCACCAGT
CACAGAAAAGCATCTTACGGATGGCATGACAGTAAGAGAATTATGCAGTGCTGCCATAACCATGAGTGATAACACTGCGGCCAACTTACTTCTGACAACG
ATTGGAGGACCGAAGGAGCTAACCGCTTTTTTGCACAACATGGGGGATCATGTAACTCGCCTTGATCGTTGGGAACCGGAGCTGAATGAAGCCATACCAA
ACGACGAGCGTGACACCACGATGCCTGTAGCAATGGCAACAACCTTGCGTAAACTATTAACTGGCGAACTACTTACTCTAGCTTCCCGGCAACAGTTGATA
GACTGGATGGAGGCGGATAAAGTTGCAGGACCACTTCTGCGCTCGGCCCTTCCGGCTGGCTGGTTTATTGCTGATAAATCTGGAGCCGGTGAGCGTGGG
TCTCGCGGTATCATTGCAGCACTGGGGCCAGATGGTAAGCCCTCCCGTATCGTAGTTATCTACACGACGGGGAGTCAGGCAACTATGGATGAACGAAATA
GACAGATCGCTGAGATAGGTGCCTCACTGATTAAGCATTGGTAACCGATTCTAGGTGCATTGGCGCAGAAAAAAATGCCTGATGCGACGCTGCGCGTCTT
ATACTCCCACATATGCCAGATTCAGCAACGGATACGGCTTCCCCAACTTGCCCACTTCCATACGTGTCCTCCTTACCAGAAATTTATCCTTAAGATCGTTTAA
ACTCGACTCTGGCTCTATCGAATCTCCGTCGTTTCGAGCTTACGCGAACAGCCGTGGCGCTCATTTGCTCGTCGGGCATCGAATCTCGTCAGCTATCGTCAG
CTTACCTTTTTGGCAGCGATCGCGGCTCCCGACATCTTGGACCATTAGCTCCACAGGTATCTTCTTCCCTCTAGTGGTCATAACAGCAGCTTCAGCTACCTCT
CAATTCAAAAAACCCCTCAAGACCCGTTTAGAGGCCCCAAGGGGTTATGCTATCAATCGTTGCGTTACACACACAAAAAACCAACACACATCCATCTTCGA
TGGATAGCGATTTTATTATCTAACTGCTGATCGAGTGTAGCCAGATCTAGTAATCAATTACGGGGTCATTAGTTCATAGCCCATATATGGAGTTCCGCGTTA
CATAACTTACGGTAAATGGCCCGCCTGGCTGACCGCCCAACGACCCCCGCCCATTGACGTCAATAATGACGTATGTTCCCATAGTAACGCCAATAGGGACT
TTCCATTGACGTCAATGGGTGGAGTATTTACGGTAAACTGCCCACTTGGCAGTACATCAAGTGTATCATATGCCAAGTACGCCCCCTATTGACGTCAATGA
CGGTAAATGGCCCGCCTGGCATTATGCCCAGTACATGACCTTATGGGACTTTCCTACTTGGCAGTACATCTACGTATTAGTCATCGCTATTACCATGCTGAT
GCGGTTTTGGCAGTACATCAATGGGCGTGGATAGCGGTTTGACTCACGGGGATTTCCAAGTCTCCACCCCATTGACGTCAATGGGAGTTTGTTTTGGCACC
AAAATCAACGGGACTTTCCAAAATGTCGTAACAACTCCGCCCCATTGACGCAAATGGGCGGTAGGCGTGTACGGTGGGAGGTCTATATAAGCAGAGCTG
GTTTAGTGAACCGTCAGATCAGATCTTTGTCGATCCTACCATCCACTCGACACACCCGCCAGCGGCCGCATGTACCCGTACGATGTCCCCGACTACGCCGG
ATCAGGCTCT

Q9NSN8

GATTTCCGAACCGCCTGCGAGGAAACCAAGACAGGGATTTGCCTCTTACAGGATGGAAATCAGGAGCCATTCAAAGTGAGGTTACACTTAGCTAAAGACA
TCCTTATGATACAGGAGCAAGATGTGATCTGCGTGTCTGGAGAACCCTTTTACTCCGGAGAACGTACCGTAACAATCCGCCGCCAGACCGTGGGGGGCTT
TGGCCTCTCAATCAAGGGCGGCGCCGAACATAATATTCCTGTTGTGGTTAGCAAGATCTCCAAAGAGCAGCGCGCTGAGCTGTCTGGACTTCTTTTTATAG
GAGACGCAATCCTCCAGATCAACGGTATCAATGTGCGCAAATGTAGACACGAGGAGGTCGTTCAGGTGTTAAGAAACGCCGGAGAAGAGGTAACCTTGA
CCGTTTCCTTCTTAAAGCGGGCACCCGCCTTTTTAAAACTGCCACTCAACGAGGACTGTGCCTGCGCACCGAGCGATCAGTCTTCCGGGACCAGTAGTCCC
CTGTGCGACTCCGGACTGCACCTGAATTATCACCCAAACAACACTGACACATTGAGCTGTTCATCTTGGCCTACATCACCTGGACTGCGGTGGGAAAAGCG
GTGGTGTGACCTGCGACTTATACCGCTGCTGCACAGTCGCTTCTCCCAATACGTTCCCGGCACCGATCTCTCACGTCAAAACGCCTTTCAGGTGATAGCGG
TTGACGGAGTGTGCACTGGCATCATTCAGTGTCTGAGCGCGGAAGACTGTGTAGATTGGCTGCAAGCCATCGCCACCAACATCTCCAACTTAACCAAACAT
AATATTAAGAAGATCAATCGGAATTTTCCCGTCAACCAGCAGATAGTATACATGGGGTGGTGCGAAGCACGTGAACAGGATCCGCTGCAGGACCGCGTG
TATAGCCCCACTTTTCTCGCTCTTAGAGGCTCCTGTCTCTACAAGTTCCTGGCTCCTCCCGTCACCACGTGGGACTGGACTAGGGCTGAAAAGACTTTTAGC
GTTTACGAGATTATGTGTAAAATTCTGAAAGACTCTGACCTCCTGGACCGACGAAAGCAGTGTTTCACCGTGCAGTCTGAGAGCGGGGAGGACTTATACT
TCTCTGTTGAGCTGGAGTCAGATCTGGCTCAATGGGAAAGGGCCTTCCAAACAGCCACCTTTCTGGAGGTGGAGCGCATTCAGTGTAAAACCTACGCATG
CGTGCTGGAATCCCATCTTATGGGCCTCACCATAGACTTCAGCACTGGCTTCATTTGTTTTGACGCGGCAACAAAAGCTGTGCTTTGGAGGTATAAGTTTA
GCCAACTGAAAGGATCTTCAGACGACGGGAAATCTAAGATCAAGTTTCTGTTCCAGAACCCCGATACCAAGCAGATAGAGGCTAAGGAGCTCGAATTCTC
CAATCTCTTCGCTGTGCTCCATTGCATCCATTCTTTTTTCGCCGCAAAAGTGGCATGCCTGGACCCTCTGTTCTTGGGGAATCAGGCCACGGCATCCACCGC
CGCAAGTTCAGCCACCACCTCTAAAGCCAAGTATACCACAGGCAGTGGGTCCGATTACAAAGACCATGATGGCGATTATAAGGACCACGACATCGATTAT
AAAGATGACGATGATAAATAGGTCGAGCCGCGGCAATTCCGACGTTACTGGCCGAAGCCGCTTGGAATAAGGCCGGTGTGCGTTTGTCTATATGTTATTT
TCCACCATATTGCCGTCTTTTGGCAATGTGAGGGCCCGGAAACCTGGCCCTGTCTTCTTGACGAGCATTCCTAGGGGTCTTTCCCCTCTCGCCAAAGGAATG
CAAGGTCTGTTGAATGTCGTGAAGGAAGCAGTTCCTCTGGAAGCTTCTTGAAGACAAACAACGTCTGTAGCGACCCTTTGCAGGCAGCGGAACCCCCCAC
CTGGCAACAGGTGCCTCTGCGGCCAAAAGCCACGTGTATAAGATACACCTGCAAAGGCGGCACAACCCCAGTGCCACGTTGTGAGTTGGATAGTTGTGG
AAAGAGTCAAATGGCTCTCCTCAAGCGTATTCAACAAGGGGCTGAAGGATGCCCAGAAGGTACCCCATTGTATGGGATCTGATCTGGGGCCTCGGTGCAC
ATGCTTTACATGTGTTTAGTCGAGGTTAAAAAAACGTCTAGGCCCCCCGAACCACGGGGACGTGGTTTTCCTTTGAAAAACACGATGATAATATGGTGTCA
GTGATTAAGCCCGAGATGAAAATGAGATATTATATGGACGGGAGCGTCAATGGGCATGAGTTCACTATCGAAGGCGAGGGAACGGGACGCCCTTATGA
GGGGCACCAAGAAATGACACTCAGAGTAACTATGGCAGAAGGCGGTCCAATGCCATTTGCATTCGATCTGGTTAGCCACGTATTTTGCTACGGGCATAGA
GTTTTTACAAAATACCCCGAAGAGATCCCAGATTACTTTAAGCAGGCATTCCCTGAAGGCCTGTCCTGGGAACGATCCCTGGAGTTCGAGGACGGGGGCA
GCGCGAGTGTCTCCGCTCACATTAGTCTGAGGGGGAACACCTTTTATCATAAATCTAAATTCACAGGAGTCAATTTCCCAGCAGATGGCCCAATTATGCAG
AATCAGTCAGTTGATTGGGAGCCTTCTACGGAAAAGATCACTGCAAGCGACGGCGTGCTTAAGGGGGATGTCACCATGTATCTGAAACTGGAAGGGGGC
GGAAACCATAAGTGTCAAATGAAAACTACCTATAAGGCCGCAAAGGAGATTCTCGAGATGCCCGGCGATCATTACATCGGTCATCGATTAGTTCGTAAGA
CCGAGGGAAATATCACTGAACAGGTCGAGGACGCTGTTGCACACTCATGAGCTAGCTTGACTGACTGAGATACAGCGTACCTTCAGCTCACAGACATGAT
AAGATACATTGATGAGTTTGGACAAACCACAACTAGAATGCAGTGAAAAAAATGCTTTATTTGTGAAATTTGTGATGCTATTGCTTTATTTGTAACCATTAT
AAGCTGCAATAAACAAGTTAACAACAACAATTGCATTCATTTTATGTTTCAGGTTCAGGGGGAGGTGTGGGAGGTTTTTTAAAGCAAGTAAAACCTCTACA
AATGTGGTATTGGCCCATCTCTATCGGTATCGTAGCATAACCCCTTGGGGCCTCTAAACGGGTCTTGAGGGGTTTTTTGTGCCCCTCGGGCCGGATTGCTA
TCTACCGGCATTGGCGCAGAAAAAAATGCCTGATGCGACGCTGCGCGTCTTATACTCCCACATATGCCAGATTCAGCAACGGATACGGCTTCCCCAACTTG
CCCACTTCCATACGTGTCCTCCTTACCAGAAATTTATCCTTAAGGTCGTCAGCTATCCTGCAGGCGATCTCTCGATTTCGATCAAGACATTCCTTTAATGGTC

TTTTCTGGACACCACTAGGGGTCAGAAGTAGTTCATCAAACTTTCTTCCCTCCCTAATCTCATTGGTTACCTTGGGCTATCGAAACTTAATTAACCAGTCAAG
TCAGCTACTTGGCGAGATCGACTTGTCTGGGTTTCGACTACGCTCAGAATTGCGTCAGTCAAGTTCGATCTGGTCCTTGCTATTGCACCCGTTCTCCGATTA
CGAGTTTCATTTAAATCATGTGAGCAAAAGGCCAGCAAAAGGCCAGGAACCGTAAAAAGGCCGCGTTGCTGGCGTTTTTCCATAGGCTCCGCCCCCCTGA
CGAGCATCACAAAAATCGACGCTCAAGTCAGAGGTGGCGAAACCCGACAGGACTATAAAGATACCAGGCGTTTCCCCCTGGAAGCTCCCTCGTGCGCTCT
CCTGTTCCGACCCTGCCGCTTACCGGATACCTGTCCGCCTTTCTCCCTTCGGGAAGCGTGGCGCTTTCTCATAGCTCACGCTGTAGGTATCTCAGTTCGGTG
TAGGTCGTTCGCTCCAAGCTGGGCTGTGTGCACGAACCCCCCGTTCAGCCCGACCGCTGCGCCTTATCCGGTAACTATCGTCTTGAGTCCAACCCGGTAAG
ACACGACTTATCGCCACTGGCAGCAGCCACTGGTAACAGGATTAGCAGAGCGAGGTATGTAGGCGGTGCTACAGAGTTCTTGAAGTGGTGGCCTAACTA
CGGCTACACTAGAAGAACAGTATTTGGTATCTGCGCTCTGCTGAAGCCAGTTACCTTCGGAAAAAGAGTTGGTAGCTCTTGATCCGGCAAACAAACCACC
GCTGGTAGCGGTGGTTTTTTTGTTTGCAAGCAGCAGATTACGCGCAGAAAAAAAGGATCTCAAGAAGATCCTTTGATCTTTTCTACGGGGTCTGACGCTCA
GTGGAACGAAAACTCACGTTAAGGGATTTTGGTCATGAGATTATCAAAAAGGATCTTCACCTAGATCCTTTTAAATTAAAAATGAAGTTTTAAATCAATCTA
AAGTATATATGAGTAAACTTGGTCTGACAGTTACCAATGCTTAATCAGTGAGGCACCTATCTCAGCGATCTGTCTATTTCGTTCATCCATAGTTGCATTTAA
ATTTCCGAACTCTCCAAGGCCCTCGTCGGAAAATCTTCAAACCTTTCGTCCGATCCATCTTGCAGGCTACCTCTCGAACGAACTATCGCAAGTCTCTTGGCC
GGCCTTGCGCCTTGGCTATTGCTTGGCAGCGCCTATCGCCAGGTATTACTCCAATCCCGAATATCCGAGATCGGGATCACCCGAGAGAAGTTCAACCTACA
TCCTCAATCCCGATCTATCCGAGATCCGAGGAATATCGAAATCGGGGCGCGCCTGGCCTCCGCGCCGGGTTTTGGCGCCTCCCGCGGGCGCCCCCCTCGT
CACGGCGAGCGCTGCCACGTCAGACGAAGGGCGCAGGAGCGTCCTGATCCTTCCGCCCGGACGCTCAGGACAGCGGCCCGCTGCTCATAAGACTCGGCC
TTAGAACCCCAGTATCAGCAGAAGGACATTTTAGGACGGGACTTGGGTGACTCTAGGGCACTGGTTTTCTTTCCAGAGAGCGGAACAGGCGAGGAAAAG
TAGTCCCTTCTCGGCGATTCTGCGGAGGGATCTCCGTGGGGCGGTGAACGCCGATGATTATATAAGGACGCGCCGGGTGTGGCACAGCTAGTTCCGTCG
CAGCCGGGATTTGGGTCGCGGTTCTTGTTTGTGGATCGCTGTGATCGTCACTTGGTGAGTAGCGGGCTGCTGGGCTGGCCGGGGCTTTCGTGGCCGCCG
GGCCGCTCGGTGGGACGGAAGCGTGTGGAGAGACCGCCAAGGGCTGTAGTCTGGGTCCGCGAGCAAGGTTGCCCTGAACTGGGGGTTGGGGGGAGCG
CAGCAAAATGGCGGCTGTTCCCGAGTCTTGAATGGAAGACGCTTGTGAGGCGGGCTGTGAGGTCGTTGAAACAAGGTGGGGGGCATGGTGGGCGGCAA
GAACCCAAGGTCTTGAGCCCTTCGCTAATGCGGGAAAGCTCTTATTCGGGTGAGATGGGCTGGGCACCATCTGGGGACCCTGACGTGAAGTTTGTCACTG
ACTGGAGAACTCGGTTTGTCGTCTGTTGCGGGGGCGGCAGTTATGGCGGTGCCGTTGGGCAGTGCACCCGTACCTTTGGGAGCGCGCGCCCTCGTCGTG
TCGTGACGTCACCCGTTCTGTTGGCTTATAATGCAGGGTGGGGCCACCTGCCGGTAGGTGTGCGGTAGGCTTTTCTCCGTCGCAGGACGCAGGGTTCGGG
CCTAGGGTAGGCTCTCCTGAATCGACAGGCGCCGGACCTCTGGTGAGGGGAGGGATAAGTGAGGCGTCAGTTTCTTTGGTCGGTTTTATGTACCTATCTT
CTTAAGTAGCTGAAGCTCCGGTTTTGAACTATGCGCTCGGGGTTGGCGAGTGTGTTTTGTGAAGTTTTTTAGGCACCTTTTGAAATGTAATCATTTGGGTC
AATATGTAATTTTCAGTGTTAGACTTGTAAATTGTCCGCTAAATTCTGGCCGTTTTTGGCTTTTTTGTTAGACAACATGACCGAGTACAAGCCCACGGTGCG
CCTCGCCACCCGCGACGACGTCCCCAGGGCCGTACGCACCCTCGCCGCCGCGTTCGCCGACTACCCCGCCACGCGCCACACCGTCGATCCGGACCGCCAC
ATCGAGCGGGTCACCGAGCTGCAAGAACTCTTCCTCACGCGCGTCGGGCTCGACATCGGCAAGGTGTGGGTCGCGGACGACGGCGCCGCGGTGGCGGT
CTGGACCACGCCGGAGAGCGTCGAAGCGGGGGCGGTGTTCGCCGAGATCGGCCCGCGCATGGCCGAGTTGAGCGGTTCCCGGCTGGCCGCGCAGCAAC
AGATGGAGGGCCTCCTGGCGCCGCACCGGCCCAAGGAGCCCGCGTGGTTCCTGGCCACCGTCGGCGTCTCGCCCGACCACCAGGGCAAGGGTCTGGGC
AGCGCCGTCGTGCTCCCCGGAGTGGAGGCCGCCGAGCGCGCCGGGGTGCCCGCCTTCCTGGAGACCTCCGCGCCCCGCAACCTCCCCTTCTACGAGCGG
CTCGGCTTCACCGTCACCGCCGACGTCGAGGTGCCCGAAGGACCGCGCACCTGGTGCATGACCCGCAAGCCCGGTGCCTAGGCTAGTATGTAAGCCTAGT
CTTAGATAATAAAATCGCTATCCATCGAAGATGGATGTGTGTTGGTTTTTTGTGTGTGTAACGCTAGGCGCGCCTGGTGTACCGAGAACGATCCTCTCAGT
GCGAGTCTCGACGATCCATATCGTTGCTTGGCAGTCAGCCAGTCGGAATCCAGCTTGGGACCCAGGAAGTCCAATCGTCAGATATTGTACTCAAGCCTGG
TCACGGCAGCGTACCGATCTGTTTAAACCTAGATATTGATAGTCTGATCGGTCAACGTATAATCGAGTCCTAGCTTTTGCAAACATCTATCAAGAGACAGG
ATCAGCAGGAGGCTTTCGCATGAGTATTCAACATTTCCGTGTCGCCCTTATTCCCTTTTTTGCGGCATTTTGCCTTCCTGTTTTTGCTCACCCAGAAACGCTG
GTGAAAGTAAAAGATGCTGAAGATCAGTTGGGTGCGCGAGTGGGTTACATCGAACTGGATCTCAACAGCGGTAAGATCCTTGAGAGTTTTCGCCCCGAA
GAACGCTTTCCAATGATGAGCACTTTTAAAGTTCTGCTATGTGGCGCGGTATTATCCCGTATTGACGCCGGGCAAGAGCAACTCGGTCGCCGCATACACTA
TTCTCAGAATGACTTGGTTGAGTATTCACCAGTCACAGAAAAGCATCTTACGGATGGCATGACAGTAAGAGAATTATGCAGTGCTGCCATAACCATGAGT
GATAACACTGCGGCCAACTTACTTCTGACAACGATTGGAGGACCGAAGGAGCTAACCGCTTTTTTGCACAACATGGGGGATCATGTAACTCGCCTTGATC
GTTGGGAACCGGAGCTGAATGAAGCCATACCAAACGACGAGCGTGACACCACGATGCCTGTAGCAATGGCAACAACCTTGCGTAAACTATTAACTGGCG
AACTACTTACTCTAGCTTCCCGGCAACAGTTGATAGACTGGATGGAGGCGGATAAAGTTGCAGGACCACTTCTGCGCTCGGCCCTTCCGGCTGGCTGGTTT
ATTGCTGATAAATCTGGAGCCGGTGAGCGTGGGTCTCGCGGTATCATTGCAGCACTGGGGCCAGATGGTAAGCCCTCCCGTATCGTAGTTATCTACACGA
CGGGGAGTCAGGCAACTATGGATGAACGAAATAGACAGATCGCTGAGATAGGTGCCTCACTGATTAAGCATTGGTAACCGATTCTAGGTGCATTGGCGC
AGAAAAAAATGCCTGATGCGACGCTGCGCGTCTTATACTCCCACATATGCCAGATTCAGCAACGGATACGGCTTCCCCAACTTGCCCACTTCCATACGTGT
CCTCCTTACCAGAAATTTATCCTTAAGATCGTTTAAACTCGACTCTGGCTCTATCGAATCTCCGTCGTTTCGAGCTTACGCGAACAGCCGTGGCGCTCATTTG
CTCGTCGGGCATCGAATCTCGTCAGCTATCGTCAGCTTACCTTTTTGGCAGCGATCGCGGCTCCCGACATCTTGGACCATTAGCTCCACAGGTATCTTCTTC
CCTCTAGTGGTCATAACAGCAGCTTCAGCTACCTCTCAATTCAAAAAACCCCTCAAGACCCGTTTAGAGGCCCCAAGGGGTTATGCTATCAATCGTTGCGTT
ACACACACAAAAAACCAACACACATCCATCTTCGATGGATAGCGATTTTATTATCTAACTGCTGATCGAGTGTAGCCAGATCTAGTAATCAATTACGGGGT
CATTAGTTCATAGCCCATATATGGAGTTCCGCGTTACATAACTTACGGTAAATGGCCCGCCTGGCTGACCGCCCAACGACCCCCGCCCATTGACGTCAATA
ATGACGTATGTTCCCATAGTAACGCCAATAGGGACTTTCCATTGACGTCAATGGGTGGAGTATTTACGGTAAACTGCCCACTTGGCAGTACATCAAGTGTA
TCATATGCCAAGTACGCCCCCTATTGACGTCAATGACGGTAAATGGCCCGCCTGGCATTATGCCCAGTACATGACCTTATGGGACTTTCCTACTTGGCAGT
ACATCTACGTATTAGTCATCGCTATTACCATGCTGATGCGGTTTTGGCAGTACATCAATGGGCGTGGATAGCGGTTTGACTCACGGGGATTTCCAAGTCTC
CACCCCATTGACGTCAATGGGAGTTTGTTTTGGCACCAAAATCAACGGGACTTTCCAAAATGTCGTAACAACTCCGCCCCATTGACGCAAATGGGCGGTAG
GCGTGTACGGTGGGAGGTCTATATAAGCAGAGCTGGTTTAGTGAACCGTCAGATCAGATCTTTGTCGATCCTACCATCCACTCGACACACCCGCCAGCGG
CCGCATGTACCCGTACGATGTCCCCGACTACGCCGGATCAGGCTCT

Q9UPT6

GAAGCCGAGGATGTGAGCTCTTACCTGTGTACAGAATCTGACAAGATCCCCATGGCACAGAGACGGCGGTTTACTCGTGTGGAAATGGCCCGGGTTCTCA
TGGAAAGGAACCAATATAAGGAGCGGCTCATGGAATTGCAAGAGGCCGTCAGGTGGACCGAGATGATCCGAGCCTCCAGAGAGCACCCATCCGTGCAG
GAGAAGAAGAAATCAACGATTTGGCAGTTTTTCAGTCGCCTCTTCTCAAGTTCTTCATCACCCCCTCCCGCCAAACGCCCTTACCCTTCTGTGAATATTCATT
ATAAGAGCCCTACCACAGCCGGGTTTAGCCAGGGCAGTGGGTCCGATTACAAAGACCATGATGGCGATTATAAGGACCACGACATCGATTATAAAGATG
ACGATGATAAATAGGTCGAGCCGCGGCAATTCCGACGTTACTGGCCGAAGCCGCTTGGAATAAGGCCGGTGTGCGTTTGTCTATATGTTATTTTCCACCAT
ATTGCCGTCTTTTGGCAATGTGAGGGCCCGGAAACCTGGCCCTGTCTTCTTGACGAGCATTCCTAGGGGTCTTTCCCCTCTCGCCAAAGGAATGCAAGGTC
TGTTGAATGTCGTGAAGGAAGCAGTTCCTCTGGAAGCTTCTTGAAGACAAACAACGTCTGTAGCGACCCTTTGCAGGCAGCGGAACCCCCCACCTGGCAA
CAGGTGCCTCTGCGGCCAAAAGCCACGTGTATAAGATACACCTGCAAAGGCGGCACAACCCCAGTGCCACGTTGTGAGTTGGATAGTTGTGGAAAGAGT

CAAATGGCTCTCCTCAAGCGTATTCAACAAGGGGCTGAAGGATGCCCAGAAGGTACCCCATTGTATGGGATCTGATCTGGGGCCTCGGTGCACATGCTTT
ACATGTGTTTAGTCGAGGTTAAAAAAACGTCTAGGCCCCCCGAACCACGGGGACGTGGTTTTCCTTTGAAAAACACGATGATAATATGGTGTCAGTGATT
AAGCCCGAGATGAAAATGAGATATTATATGGACGGGAGCGTCAATGGGCATGAGTTCACTATCGAAGGCGAGGGAACGGGACGCCCTTATGAGGGGCA
CCAAGAAATGACACTCAGAGTAACTATGGCAGAAGGCGGTCCAATGCCATTTGCATTCGATCTGGTTAGCCACGTATTTTGCTACGGGCATAGAGTTTTTA
CAAAATACCCCGAAGAGATCCCAGATTACTTTAAGCAGGCATTCCCTGAAGGCCTGTCCTGGGAACGATCCCTGGAGTTCGAGGACGGGGGCAGCGCGA
GTGTCTCCGCTCACATTAGTCTGAGGGGGAACACCTTTTATCATAAATCTAAATTCACAGGAGTCAATTTCCCAGCAGATGGCCCAATTATGCAGAATCAG
TCAGTTGATTGGGAGCCTTCTACGGAAAAGATCACTGCAAGCGACGGCGTGCTTAAGGGGGATGTCACCATGTATCTGAAACTGGAAGGGGGCGGAAAC
CATAAGTGTCAAATGAAAACTACCTATAAGGCCGCAAAGGAGATTCTCGAGATGCCCGGCGATCATTACATCGGTCATCGATTAGTTCGTAAGACCGAGG
GAAATATCACTGAACAGGTCGAGGACGCTGTTGCACACTCATGAGCTAGCTTGACTGACTGAGATACAGCGTACCTTCAGCTCACAGACATGATAAGATA
CATTGATGAGTTTGGACAAACCACAACTAGAATGCAGTGAAAAAAATGCTTTATTTGTGAAATTTGTGATGCTATTGCTTTATTTGTAACCATTATAAGCTG
CAATAAACAAGTTAACAACAACAATTGCATTCATTTTATGTTTCAGGTTCAGGGGGAGGTGTGGGAGGTTTTTTAAAGCAAGTAAAACCTCTACAAATGTG
GTATTGGCCCATCTCTATCGGTATCGTAGCATAACCCCTTGGGGCCTCTAAACGGGTCTTGAGGGGTTTTTTGTGCCCCTCGGGCCGGATTGCTATCTACC
GGCATTGGCGCAGAAAAAAATGCCTGATGCGACGCTGCGCGTCTTATACTCCCACATATGCCAGATTCAGCAACGGATACGGCTTCCCCAACTTGCCCACT
TCCATACGTGTCCTCCTTACCAGAAATTTATCCTTAAGGTCGTCAGCTATCCTGCAGGCGATCTCTCGATTTCGATCAAGACATTCCTTTAATGGTCTTTTCT
GGACACCACTAGGGGTCAGAAGTAGTTCATCAAACTTTCTTCCCTCCCTAATCTCATTGGTTACCTTGGGCTATCGAAACTTAATTAACCAGTCAAGTCAGC
TACTTGGCGAGATCGACTTGTCTGGGTTTCGACTACGCTCAGAATTGCGTCAGTCAAGTTCGATCTGGTCCTTGCTATTGCACCCGTTCTCCGATTACGAGT
TTCATTTAAATCATGTGAGCAAAAGGCCAGCAAAAGGCCAGGAACCGTAAAAAGGCCGCGTTGCTGGCGTTTTTCCATAGGCTCCGCCCCCCTGACGAGC
ATCACAAAAATCGACGCTCAAGTCAGAGGTGGCGAAACCCGACAGGACTATAAAGATACCAGGCGTTTCCCCCTGGAAGCTCCCTCGTGCGCTCTCCTGT
TCCGACCCTGCCGCTTACCGGATACCTGTCCGCCTTTCTCCCTTCGGGAAGCGTGGCGCTTTCTCATAGCTCACGCTGTAGGTATCTCAGTTCGGTGTAGGT
CGTTCGCTCCAAGCTGGGCTGTGTGCACGAACCCCCCGTTCAGCCCGACCGCTGCGCCTTATCCGGTAACTATCGTCTTGAGTCCAACCCGGTAAGACACG
ACTTATCGCCACTGGCAGCAGCCACTGGTAACAGGATTAGCAGAGCGAGGTATGTAGGCGGTGCTACAGAGTTCTTGAAGTGGTGGCCTAACTACGGCT
ACACTAGAAGAACAGTATTTGGTATCTGCGCTCTGCTGAAGCCAGTTACCTTCGGAAAAAGAGTTGGTAGCTCTTGATCCGGCAAACAAACCACCGCTGG
TAGCGGTGGTTTTTTTGTTTGCAAGCAGCAGATTACGCGCAGAAAAAAAGGATCTCAAGAAGATCCTTTGATCTTTTCTACGGGGTCTGACGCTCAGTGGA
ACGAAAACTCACGTTAAGGGATTTTGGTCATGAGATTATCAAAAAGGATCTTCACCTAGATCCTTTTAAATTAAAAATGAAGTTTTAAATCAATCTAAAGTA
TATATGAGTAAACTTGGTCTGACAGTTACCAATGCTTAATCAGTGAGGCACCTATCTCAGCGATCTGTCTATTTCGTTCATCCATAGTTGCATTTAAATTTCC
GAACTCTCCAAGGCCCTCGTCGGAAAATCTTCAAACCTTTCGTCCGATCCATCTTGCAGGCTACCTCTCGAACGAACTATCGCAAGTCTCTTGGCCGGCCTT
GCGCCTTGGCTATTGCTTGGCAGCGCCTATCGCCAGGTATTACTCCAATCCCGAATATCCGAGATCGGGATCACCCGAGAGAAGTTCAACCTACATCCTCA
ATCCCGATCTATCCGAGATCCGAGGAATATCGAAATCGGGGCGCGCCTGGCCTCCGCGCCGGGTTTTGGCGCCTCCCGCGGGCGCCCCCCTCGTCACGGC
GAGCGCTGCCACGTCAGACGAAGGGCGCAGGAGCGTCCTGATCCTTCCGCCCGGACGCTCAGGACAGCGGCCCGCTGCTCATAAGACTCGGCCTTAGAA
CCCCAGTATCAGCAGAAGGACATTTTAGGACGGGACTTGGGTGACTCTAGGGCACTGGTTTTCTTTCCAGAGAGCGGAACAGGCGAGGAAAAGTAGTCC
CTTCTCGGCGATTCTGCGGAGGGATCTCCGTGGGGCGGTGAACGCCGATGATTATATAAGGACGCGCCGGGTGTGGCACAGCTAGTTCCGTCGCAGCCG
GGATTTGGGTCGCGGTTCTTGTTTGTGGATCGCTGTGATCGTCACTTGGTGAGTAGCGGGCTGCTGGGCTGGCCGGGGCTTTCGTGGCCGCCGGGCCGCT
CGGTGGGACGGAAGCGTGTGGAGAGACCGCCAAGGGCTGTAGTCTGGGTCCGCGAGCAAGGTTGCCCTGAACTGGGGGTTGGGGGGAGCGCAGCAAA
ATGGCGGCTGTTCCCGAGTCTTGAATGGAAGACGCTTGTGAGGCGGGCTGTGAGGTCGTTGAAACAAGGTGGGGGGCATGGTGGGCGGCAAGAACCCA
AGGTCTTGAGCCCTTCGCTAATGCGGGAAAGCTCTTATTCGGGTGAGATGGGCTGGGCACCATCTGGGGACCCTGACGTGAAGTTTGTCACTGACTGGAG
AACTCGGTTTGTCGTCTGTTGCGGGGGCGGCAGTTATGGCGGTGCCGTTGGGCAGTGCACCCGTACCTTTGGGAGCGCGCGCCCTCGTCGTGTCGTGAC
GTCACCCGTTCTGTTGGCTTATAATGCAGGGTGGGGCCACCTGCCGGTAGGTGTGCGGTAGGCTTTTCTCCGTCGCAGGACGCAGGGTTCGGGCCTAGG
GTAGGCTCTCCTGAATCGACAGGCGCCGGACCTCTGGTGAGGGGAGGGATAAGTGAGGCGTCAGTTTCTTTGGTCGGTTTTATGTACCTATCTTCTTAAGT
AGCTGAAGCTCCGGTTTTGAACTATGCGCTCGGGGTTGGCGAGTGTGTTTTGTGAAGTTTTTTAGGCACCTTTTGAAATGTAATCATTTGGGTCAATATGT
AATTTTCAGTGTTAGACTTGTAAATTGTCCGCTAAATTCTGGCCGTTTTTGGCTTTTTTGTTAGACAACATGACCGAGTACAAGCCCACGGTGCGCCTCGCC
ACCCGCGACGACGTCCCCAGGGCCGTACGCACCCTCGCCGCCGCGTTCGCCGACTACCCCGCCACGCGCCACACCGTCGATCCGGACCGCCACATCGAGC
GGGTCACCGAGCTGCAAGAACTCTTCCTCACGCGCGTCGGGCTCGACATCGGCAAGGTGTGGGTCGCGGACGACGGCGCCGCGGTGGCGGTCTGGACC
ACGCCGGAGAGCGTCGAAGCGGGGGCGGTGTTCGCCGAGATCGGCCCGCGCATGGCCGAGTTGAGCGGTTCCCGGCTGGCCGCGCAGCAACAGATGG
AGGGCCTCCTGGCGCCGCACCGGCCCAAGGAGCCCGCGTGGTTCCTGGCCACCGTCGGCGTCTCGCCCGACCACCAGGGCAAGGGTCTGGGCAGCGCCG
TCGTGCTCCCCGGAGTGGAGGCCGCCGAGCGCGCCGGGGTGCCCGCCTTCCTGGAGACCTCCGCGCCCCGCAACCTCCCCTTCTACGAGCGGCTCGGCTT
CACCGTCACCGCCGACGTCGAGGTGCCCGAAGGACCGCGCACCTGGTGCATGACCCGCAAGCCCGGTGCCTAGGCTAGTATGTAAGCCTAGTCTTAGATA
ATAAAATCGCTATCCATCGAAGATGGATGTGTGTTGGTTTTTTGTGTGTGTAACGCTAGGCGCGCCTGGTGTACCGAGAACGATCCTCTCAGTGCGAGTCT
CGACGATCCATATCGTTGCTTGGCAGTCAGCCAGTCGGAATCCAGCTTGGGACCCAGGAAGTCCAATCGTCAGATATTGTACTCAAGCCTGGTCACGGCA
GCGTACCGATCTGTTTAAACCTAGATATTGATAGTCTGATCGGTCAACGTATAATCGAGTCCTAGCTTTTGCAAACATCTATCAAGAGACAGGATCAGCAG
GAGGCTTTCGCATGAGTATTCAACATTTCCGTGTCGCCCTTATTCCCTTTTTTGCGGCATTTTGCCTTCCTGTTTTTGCTCACCCAGAAACGCTGGTGAAAGT
AAAAGATGCTGAAGATCAGTTGGGTGCGCGAGTGGGTTACATCGAACTGGATCTCAACAGCGGTAAGATCCTTGAGAGTTTTCGCCCCGAAGAACGCTTT
CCAATGATGAGCACTTTTAAAGTTCTGCTATGTGGCGCGGTATTATCCCGTATTGACGCCGGGCAAGAGCAACTCGGTCGCCGCATACACTATTCTCAGAA
TGACTTGGTTGAGTATTCACCAGTCACAGAAAAGCATCTTACGGATGGCATGACAGTAAGAGAATTATGCAGTGCTGCCATAACCATGAGTGATAACACT
GCGGCCAACTTACTTCTGACAACGATTGGAGGACCGAAGGAGCTAACCGCTTTTTTGCACAACATGGGGGATCATGTAACTCGCCTTGATCGTTGGGAAC
CGGAGCTGAATGAAGCCATACCAAACGACGAGCGTGACACCACGATGCCTGTAGCAATGGCAACAACCTTGCGTAAACTATTAACTGGCGAACTACTTAC
TCTAGCTTCCCGGCAACAGTTGATAGACTGGATGGAGGCGGATAAAGTTGCAGGACCACTTCTGCGCTCGGCCCTTCCGGCTGGCTGGTTTATTGCTGAT
AAATCTGGAGCCGGTGAGCGTGGGTCTCGCGGTATCATTGCAGCACTGGGGCCAGATGGTAAGCCCTCCCGTATCGTAGTTATCTACACGACGGGGAGT
CAGGCAACTATGGATGAACGAAATAGACAGATCGCTGAGATAGGTGCCTCACTGATTAAGCATTGGTAACCGATTCTAGGTGCATTGGCGCAGAAAAAA
ATGCCTGATGCGACGCTGCGCGTCTTATACTCCCACATATGCCAGATTCAGCAACGGATACGGCTTCCCCAACTTGCCCACTTCCATACGTGTCCTCCTTAC
CAGAAATTTATCCTTAAGATCGTTTAAACTCGACTCTGGCTCTATCGAATCTCCGTCGTTTCGAGCTTACGCGAACAGCCGTGGCGCTCATTTGCTCGTCGG
GCATCGAATCTCGTCAGCTATCGTCAGCTTACCTTTTTGGCAGCGATCGCGGCTCCCGACATCTTGGACCATTAGCTCCACAGGTATCTTCTTCCCTCTAGTG
GTCATAACAGCAGCTTCAGCTACCTCTCAATTCAAAAAACCCCTCAAGACCCGTTTAGAGGCCCCAAGGGGTTATGCTATCAATCGTTGCGTTACACACAC
AAAAAACCAACACACATCCATCTTCGATGGATAGCGATTTTATTATCTAACTGCTGATCGAGTGTAGCCAGATCTAGTAATCAATTACGGGGTCATTAGTTC
ATAGCCCATATATGGAGTTCCGCGTTACATAACTTACGGTAAATGGCCCGCCTGGCTGACCGCCCAACGACCCCCGCCCATTGACGTCAATAATGACGTAT
GTTCCCATAGTAACGCCAATAGGGACTTTCCATTGACGTCAATGGGTGGAGTATTTACGGTAAACTGCCCACTTGGCAGTACATCAAGTGTATCATATGCC
AAGTACGCCCCCTATTGACGTCAATGACGGTAAATGGCCCGCCTGGCATTATGCCCAGTACATGACCTTATGGGACTTTCCTACTTGGCAGTACATCTACG
TATTAGTCATCGCTATTACCATGCTGATGCGGTTTTGGCAGTACATCAATGGGCGTGGATAGCGGTTTGACTCACGGGGATTTCCAAGTCTCCACCCCATT
GACGTCAATGGGAGTTTGTTTTGGCACCAAAATCAACGGGACTTTCCAAAATGTCGTAACAACTCCGCCCCATTGACGCAAATGGGCGGTAGGCGTGTAC
GGTGGGAGGTCTATATAAGCAGAGCTGGTTTAGTGAACCGTCAGATCAGATCTTTGTCGATCCTACCATCCACTCGACACACCCGCCAGCGGCCGCATGT
ACCCGTACGATGTCCCCGACTACGCCGGATCAGGCTCT

Q8IXF0

GCCCCTACAAAGCCTAGCTTCCAACAGGATCCAAGTCGACGGGAACGGATCACAGCCCAGCATCCCCTGCCCAACCAGAGCGAGTGCAGAAAGATCTACC
GATATGACGGAATATATTGCGAGAGTACCTATCAAAATCTGCAGGCCCTCAGGAAAGAGAAGAGTAGAGACGCTGCTCGGTCTAGAAGGGGAAAAGAG
AACTTCGAATTTTATGAGCTCGCCAAATTACTGCCTCTGCCTGCCGCGATTACAAGCCAGCTCGACAAAGCAAGTATTATACGTTTGACCATCAGCTACCTG
AAGATGCGAGATTTTGCAAACCAGGGTGATCCTCCCTGGAACCTGAGAATGGAAGGACCCCCGCCCAATACTTCCGTAAAAGTGATTGGGGCGCAGCGA
CGACGATCTCCTAGTGCCCTGGCTATTGAGGTGTTCGAGGCCCACTTGGGATCCCATATCCTGCAGAGCCTTGATGGCTTCGTGTTCGCCCTGAACCAGGA
AGGGAAGTTTTTATACATCTCAGAGACCGTGTCCATCTATCTGGGTTTGTCCCAGGTTGAATTGACTGGGTCTAGCGTATTTGATTATGTGCACCCAGGCG
ATCATGTTGAAATGGCAGAACAATTGGGGATGAAGCTGCCACCCGGGAGGGGCCTCCTGAGTCAGGGGACGGCAGAAGACGGAGCGTCCTCTGCCTCC
AGTAGCTCTCAGAGTGAGACTCCCGAACCAGTGGAGTCCACATCCCCAAGCCTCTTGACTACGGATAATACCCTGGAACGGTCCTTTTTCATTCGTATGAA
AAGCACCCTTACTAAGCGAGGCGTGCATATTAAGTCTTCTGGGTACAAAGTCATCCACATCACAGGCAGGTTGAGGCTGCGAGTTAGCTTGAGTCACGGC
CGAACCGTGCCTTCCCAGATTATGGGGCTGGTCGTGGTTGCTCACGCACTTCCGCCACCAACTATCAATGAGGTGCGGATCGATTGCCACATGTTTGTAAC
TCGGGTCAATATGGATTTGAATATCATCTATTGCGAGAACCGCATTAGCGATTACATGGACCTTACGCCCGTCGATATCGTGGGCAAGAGATGCTACCACT
TTATCCACGCTGAGGACGTGGAAGGGATACGGCACTCCCATCTGGATCTTCTCAATAAAGGACAATGCGTGACCAAGTATTACCGATGGATGCAAAAAAA
TGGGGGATATATCTGGATACAGAGCTCTGCCACCATAGCAATTAACGCTAAGAATGGCAGTGGGTCCGATTACAAAGACCATGATGGCGATTATAAGGA
CCACGACATCGATTATAAAGATGACGATGATAAATAGGTCGAGCCGCGGCAATTCCGACGTTACTGGCCGAAGCCGCTTGGAATAAGGCCGGTGTGCGT
TTGTCTATATGTTATTTTCCACCATATTGCCGTCTTTTGGCAATGTGAGGGCCCGGAAACCTGGCCCTGTCTTCTTGACGAGCATTCCTAGGGGTCTTTCCCC
TCTCGCCAAAGGAATGCAAGGTCTGTTGAATGTCGTGAAGGAAGCAGTTCCTCTGGAAGCTTCTTGAAGACAAACAACGTCTGTAGCGACCCTTTGCAGG
CAGCGGAACCCCCCACCTGGCAACAGGTGCCTCTGCGGCCAAAAGCCACGTGTATAAGATACACCTGCAAAGGCGGCACAACCCCAGTGCCACGTTGTG
AGTTGGATAGTTGTGGAAAGAGTCAAATGGCTCTCCTCAAGCGTATTCAACAAGGGGCTGAAGGATGCCCAGAAGGTACCCCATTGTATGGGATCTGATC
TGGGGCCTCGGTGCACATGCTTTACATGTGTTTAGTCGAGGTTAAAAAAACGTCTAGGCCCCCCGAACCACGGGGACGTGGTTTTCCTTTGAAAAACACG
ATGATAATATGGTGTCAGTGATTAAGCCCGAGATGAAAATGAGATATTATATGGACGGGAGCGTCAATGGGCATGAGTTCACTATCGAAGGCGAGGGAA
CGGGACGCCCTTATGAGGGGCACCAAGAAATGACACTCAGAGTAACTATGGCAGAAGGCGGTCCAATGCCATTTGCATTCGATCTGGTTAGCCACGTATT
TTGCTACGGGCATAGAGTTTTTACAAAATACCCCGAAGAGATCCCAGATTACTTTAAGCAGGCATTCCCTGAAGGCCTGTCCTGGGAACGATCCCTGGAGT
TCGAGGACGGGGGCAGCGCGAGTGTCTCCGCTCACATTAGTCTGAGGGGGAACACCTTTTATCATAAATCTAAATTCACAGGAGTCAATTTCCCAGCAGA
TGGCCCAATTATGCAGAATCAGTCAGTTGATTGGGAGCCTTCTACGGAAAAGATCACTGCAAGCGACGGCGTGCTTAAGGGGGATGTCACCATGTATCTG
AAACTGGAAGGGGGCGGAAACCATAAGTGTCAAATGAAAACTACCTATAAGGCCGCAAAGGAGATTCTCGAGATGCCCGGCGATCATTACATCGGTCAT
CGATTAGTTCGTAAGACCGAGGGAAATATCACTGAACAGGTCGAGGACGCTGTTGCACACTCATGAGCTAGCTTGACTGACTGAGATACAGCGTACCTTC
AGCTCACAGACATGATAAGATACATTGATGAGTTTGGACAAACCACAACTAGAATGCAGTGAAAAAAATGCTTTATTTGTGAAATTTGTGATGCTATTGCT
TTATTTGTAACCATTATAAGCTGCAATAAACAAGTTAACAACAACAATTGCATTCATTTTATGTTTCAGGTTCAGGGGGAGGTGTGGGAGGTTTTTTAAAG
CAAGTAAAACCTCTACAAATGTGGTATTGGCCCATCTCTATCGGTATCGTAGCATAACCCCTTGGGGCCTCTAAACGGGTCTTGAGGGGTTTTTTGTGCCCC
TCGGGCCGGATTGCTATCTACCGGCATTGGCGCAGAAAAAAATGCCTGATGCGACGCTGCGCGTCTTATACTCCCACATATGCCAGATTCAGCAACGGAT
ACGGCTTCCCCAACTTGCCCACTTCCATACGTGTCCTCCTTACCAGAAATTTATCCTTAAGGTCGTCAGCTATCCTGCAGGCGATCTCTCGATTTCGATCAAG
ACATTCCTTTAATGGTCTTTTCTGGACACCACTAGGGGTCAGAAGTAGTTCATCAAACTTTCTTCCCTCCCTAATCTCATTGGTTACCTTGGGCTATCGAAAC
TTAATTAACCAGTCAAGTCAGCTACTTGGCGAGATCGACTTGTCTGGGTTTCGACTACGCTCAGAATTGCGTCAGTCAAGTTCGATCTGGTCCTTGCTATTG
CACCCGTTCTCCGATTACGAGTTTCATTTAAATCATGTGAGCAAAAGGCCAGCAAAAGGCCAGGAACCGTAAAAAGGCCGCGTTGCTGGCGTTTTTCCATA
GGCTCCGCCCCCCTGACGAGCATCACAAAAATCGACGCTCAAGTCAGAGGTGGCGAAACCCGACAGGACTATAAAGATACCAGGCGTTTCCCCCTGGAA
GCTCCCTCGTGCGCTCTCCTGTTCCGACCCTGCCGCTTACCGGATACCTGTCCGCCTTTCTCCCTTCGGGAAGCGTGGCGCTTTCTCATAGCTCACGCTGTA
GGTATCTCAGTTCGGTGTAGGTCGTTCGCTCCAAGCTGGGCTGTGTGCACGAACCCCCCGTTCAGCCCGACCGCTGCGCCTTATCCGGTAACTATCGTCTT
GAGTCCAACCCGGTAAGACACGACTTATCGCCACTGGCAGCAGCCACTGGTAACAGGATTAGCAGAGCGAGGTATGTAGGCGGTGCTACAGAGTTCTTG
AAGTGGTGGCCTAACTACGGCTACACTAGAAGAACAGTATTTGGTATCTGCGCTCTGCTGAAGCCAGTTACCTTCGGAAAAAGAGTTGGTAGCTCTTGAT
CCGGCAAACAAACCACCGCTGGTAGCGGTGGTTTTTTTGTTTGCAAGCAGCAGATTACGCGCAGAAAAAAAGGATCTCAAGAAGATCCTTTGATCTTTTCT
ACGGGGTCTGACGCTCAGTGGAACGAAAACTCACGTTAAGGGATTTTGGTCATGAGATTATCAAAAAGGATCTTCACCTAGATCCTTTTAAATTAAAAATG
AAGTTTTAAATCAATCTAAAGTATATATGAGTAAACTTGGTCTGACAGTTACCAATGCTTAATCAGTGAGGCACCTATCTCAGCGATCTGTCTATTTCGTTC
ATCCATAGTTGCATTTAAATTTCCGAACTCTCCAAGGCCCTCGTCGGAAAATCTTCAAACCTTTCGTCCGATCCATCTTGCAGGCTACCTCTCGAACGAACTA
TCGCAAGTCTCTTGGCCGGCCTTGCGCCTTGGCTATTGCTTGGCAGCGCCTATCGCCAGGTATTACTCCAATCCCGAATATCCGAGATCGGGATCACCCGA
GAGAAGTTCAACCTACATCCTCAATCCCGATCTATCCGAGATCCGAGGAATATCGAAATCGGGGCGCGCCTGGCCTCCGCGCCGGGTTTTGGCGCCTCCC
GCGGGCGCCCCCCTCGTCACGGCGAGCGCTGCCACGTCAGACGAAGGGCGCAGGAGCGTCCTGATCCTTCCGCCCGGACGCTCAGGACAGCGGCCCGCT
GCTCATAAGACTCGGCCTTAGAACCCCAGTATCAGCAGAAGGACATTTTAGGACGGGACTTGGGTGACTCTAGGGCACTGGTTTTCTTTCCAGAGAGCGG
AACAGGCGAGGAAAAGTAGTCCCTTCTCGGCGATTCTGCGGAGGGATCTCCGTGGGGCGGTGAACGCCGATGATTATATAAGGACGCGCCGGGTGTGG
CACAGCTAGTTCCGTCGCAGCCGGGATTTGGGTCGCGGTTCTTGTTTGTGGATCGCTGTGATCGTCACTTGGTGAGTAGCGGGCTGCTGGGCTGGCCGGG
GCTTTCGTGGCCGCCGGGCCGCTCGGTGGGACGGAAGCGTGTGGAGAGACCGCCAAGGGCTGTAGTCTGGGTCCGCGAGCAAGGTTGCCCTGAACTGG
GGGTTGGGGGGAGCGCAGCAAAATGGCGGCTGTTCCCGAGTCTTGAATGGAAGACGCTTGTGAGGCGGGCTGTGAGGTCGTTGAAACAAGGTGGGGG
GCATGGTGGGCGGCAAGAACCCAAGGTCTTGAGCCCTTCGCTAATGCGGGAAAGCTCTTATTCGGGTGAGATGGGCTGGGCACCATCTGGGGACCCTGA
CGTGAAGTTTGTCACTGACTGGAGAACTCGGTTTGTCGTCTGTTGCGGGGGCGGCAGTTATGGCGGTGCCGTTGGGCAGTGCACCCGTACCTTTGGGAG
CGCGCGCCCTCGTCGTGTCGTGACGTCACCCGTTCTGTTGGCTTATAATGCAGGGTGGGGCCACCTGCCGGTAGGTGTGCGGTAGGCTTTTCTCCGTCGC
AGGACGCAGGGTTCGGGCCTAGGGTAGGCTCTCCTGAATCGACAGGCGCCGGACCTCTGGTGAGGGGAGGGATAAGTGAGGCGTCAGTTTCTTTGGTC
GGTTTTATGTACCTATCTTCTTAAGTAGCTGAAGCTCCGGTTTTGAACTATGCGCTCGGGGTTGGCGAGTGTGTTTTGTGAAGTTTTTTAGGCACCTTTTGA
AATGTAATCATTTGGGTCAATATGTAATTTTCAGTGTTAGACTTGTAAATTGTCCGCTAAATTCTGGCCGTTTTTGGCTTTTTTGTTAGACAACATGACCGAG
TACAAGCCCACGGTGCGCCTCGCCACCCGCGACGACGTCCCCAGGGCCGTACGCACCCTCGCCGCCGCGTTCGCCGACTACCCCGCCACGCGCCACACCG
TCGATCCGGACCGCCACATCGAGCGGGTCACCGAGCTGCAAGAACTCTTCCTCACGCGCGTCGGGCTCGACATCGGCAAGGTGTGGGTCGCGGACGACG
GCGCCGCGGTGGCGGTCTGGACCACGCCGGAGAGCGTCGAAGCGGGGGCGGTGTTCGCCGAGATCGGCCCGCGCATGGCCGAGTTGAGCGGTTCCCG
GCTGGCCGCGCAGCAACAGATGGAGGGCCTCCTGGCGCCGCACCGGCCCAAGGAGCCCGCGTGGTTCCTGGCCACCGTCGGCGTCTCGCCCGACCACCA
GGGCAAGGGTCTGGGCAGCGCCGTCGTGCTCCCCGGAGTGGAGGCCGCCGAGCGCGCCGGGGTGCCCGCCTTCCTGGAGACCTCCGCGCCCCGCAACC
TCCCCTTCTACGAGCGGCTCGGCTTCACCGTCACCGCCGACGTCGAGGTGCCCGAAGGACCGCGCACCTGGTGCATGACCCGCAAGCCCGGTGCCTAGGC
TAGTATGTAAGCCTAGTCTTAGATAATAAAATCGCTATCCATCGAAGATGGATGTGTGTTGGTTTTTTGTGTGTGTAACGCTAGGCGCGCCTGGTGTACCG
AGAACGATCCTCTCAGTGCGAGTCTCGACGATCCATATCGTTGCTTGGCAGTCAGCCAGTCGGAATCCAGCTTGGGACCCAGGAAGTCCAATCGTCAGAT
ATTGTACTCAAGCCTGGTCACGGCAGCGTACCGATCTGTTTAAACCTAGATATTGATAGTCTGATCGGTCAACGTATAATCGAGTCCTAGCTTTTGCAAACA
TCTATCAAGAGACAGGATCAGCAGGAGGCTTTCGCATGAGTATTCAACATTTCCGTGTCGCCCTTATTCCCTTTTTTGCGGCATTTTGCCTTCCTGTTTTTGC
TCACCCAGAAACGCTGGTGAAAGTAAAAGATGCTGAAGATCAGTTGGGTGCGCGAGTGGGTTACATCGAACTGGATCTCAACAGCGGTAAGATCCTTGA
GAGTTTTCGCCCCGAAGAACGCTTTCCAATGATGAGCACTTTTAAAGTTCTGCTATGTGGCGCGGTATTATCCCGTATTGACGCCGGGCAAGAGCAACTCG

GTCGCCGCATACACTATTCTCAGAATGACTTGGTTGAGTATTCACCAGTCACAGAAAAGCATCTTACGGATGGCATGACAGTAAGAGAATTATGCAGTGCT
GCCATAACCATGAGTGATAACACTGCGGCCAACTTACTTCTGACAACGATTGGAGGACCGAAGGAGCTAACCGCTTTTTTGCACAACATGGGGGATCATG
TAACTCGCCTTGATCGTTGGGAACCGGAGCTGAATGAAGCCATACCAAACGACGAGCGTGACACCACGATGCCTGTAGCAATGGCAACAACCTTGCGTAA
ACTATTAACTGGCGAACTACTTACTCTAGCTTCCCGGCAACAGTTGATAGACTGGATGGAGGCGGATAAAGTTGCAGGACCACTTCTGCGCTCGGCCCTTC
CGGCTGGCTGGTTTATTGCTGATAAATCTGGAGCCGGTGAGCGTGGGTCTCGCGGTATCATTGCAGCACTGGGGCCAGATGGTAAGCCCTCCCGTATCGT
AGTTATCTACACGACGGGGAGTCAGGCAACTATGGATGAACGAAATAGACAGATCGCTGAGATAGGTGCCTCACTGATTAAGCATTGGTAACCGATTCTA
GGTGCATTGGCGCAGAAAAAAATGCCTGATGCGACGCTGCGCGTCTTATACTCCCACATATGCCAGATTCAGCAACGGATACGGCTTCCCCAACTTGCCCA
CTTCCATACGTGTCCTCCTTACCAGAAATTTATCCTTAAGATCGTTTAAACTCGACTCTGGCTCTATCGAATCTCCGTCGTTTCGAGCTTACGCGAACAGCCG
TGGCGCTCATTTGCTCGTCGGGCATCGAATCTCGTCAGCTATCGTCAGCTTACCTTTTTGGCAGCGATCGCGGCTCCCGACATCTTGGACCATTAGCTCCAC
AGGTATCTTCTTCCCTCTAGTGGTCATAACAGCAGCTTCAGCTACCTCTCAATTCAAAAAACCCCTCAAGACCCGTTTAGAGGCCCCAAGGGGTTATGCTAT
CAATCGTTGCGTTACACACACAAAAAACCAACACACATCCATCTTCGATGGATAGCGATTTTATTATCTAACTGCTGATCGAGTGTAGCCAGATCTAGTAAT
CAATTACGGGGTCATTAGTTCATAGCCCATATATGGAGTTCCGCGTTACATAACTTACGGTAAATGGCCCGCCTGGCTGACCGCCCAACGACCCCCGCCCA
TTGACGTCAATAATGACGTATGTTCCCATAGTAACGCCAATAGGGACTTTCCATTGACGTCAATGGGTGGAGTATTTACGGTAAACTGCCCACTTGGCAGT
ACATCAAGTGTATCATATGCCAAGTACGCCCCCTATTGACGTCAATGACGGTAAATGGCCCGCCTGGCATTATGCCCAGTACATGACCTTATGGGACTTTC
CTACTTGGCAGTACATCTACGTATTAGTCATCGCTATTACCATGCTGATGCGGTTTTGGCAGTACATCAATGGGCGTGGATAGCGGTTTGACTCACGGGGA
TTTCCAAGTCTCCACCCCATTGACGTCAATGGGAGTTTGTTTTGGCACCAAAATCAACGGGACTTTCCAAAATGTCGTAACAACTCCGCCCCATTGACGCAA
ATGGGCGGTAGGCGTGTACGGTGGGAGGTCTATATAAGCAGAGCTGGTTTAGTGAACCGTCAGATCAGATCTTTGTCGATCCTACCATCCACTCGACACA
CCCGCCAGCGGCCGCATGTACCCGTACGATGTCCCCGACTACGCCGGATCAGGCTCT

Q8IZD9

TGGACTCCAACAGAAGAAGAGAAATATGGAGTCGTCATCTGCTCTTTCCGAGGCAGCGTGCCACAGGGCCTCGTTCTTGAAATCGGTGAAACAGTTCAAA
TCCTGGAGAAGTGCGAGGGCTGGTACCGAGGTGTGAGTACAAAGAAACCTAATGTTAAGGGTATTTTCCCAGCCAACTATATCCATTTGAAAAAAGCCAT
TGTCAGCAATCGCGGACAGTATGAGACTGTGGTGCCGTTGGAAGACAGCATAGTTACTGAAGTGACGGCAACACTTCAGGAATGGGCGTCTCTTTGGAA
GCAGCTGTATGTGAAGCACAAAGTCGATCTGTTTTACAAACTCCGCCATGTAATGAATGAATTGATTGACCTTCGCCGTCAGTTACTCAGCGGTCATCTGA
CACAGGATCAGGTGCGCGAAGTTAAGAGGCATATCACGGTGAGGTTGGACTGGGGCAATGAACACTTGGGGCTGGATCTGGTGCCGCGTAAGGACTTT
GAAGTCGTTGACAGTGATCAGATTAGCGTGAGTGATCTTTATAAGATGCATCTGTCAAGCAGACAGTCTGTGCAGCAGAGCACATCCCAAGTGGACACGA
TGCGTCCTCGCCACGGAGAAACGTGTCGGATGCCTGTGCCACACCACTTCTTCCTTTCTCTGAAATCCTTCACATATAATACAATCGGGGAGGATACTGAT
GTCTTTTTCAGTTTGTACGATATGCGCGAAGGGAAACAAATCTCAGAGAGATTCCTCGTAAGACTGAATAAGAACGGCGGCCCACGGAACCCAGAGAAG
ATAGAGAGAATGTGCGCCCTCTTTACAGACCTGAGCTCTAAGGACATGAAAAGGGATCTTTATATTGTGGCACATGTGATCCGAATCGGGAGAATGCTCC
TGAACGACAGCAAGAAGGGACCTCCTCACTTACATTATAGACGCCCATATGGCTGTGCCGTCCTCTCAATCTTGGACGTTCTGCAGTCACTGACCGAAGTC
AAGGAGGAAAAAGATTTCGTCTTAAAAGTGTATACCTGTAACAACGAATCTGAGTGGAGCCAGATTCACGAAAATATCATACGCAAATCCTCTGCGAAAT
ACTCTGCCCCCAGCGCATCCCATGGCCTCATCATTTCTCTGCAGTTATTGCGCGGAGATATGGAGCAGATCCGCAGAGAAAATCCTATGATCTTTAATCGG
GGGCTGGCCATCACCAGAAAGTTAGGATTCCCTGACGTAATTATGCCAGGCGATATTGGCAGTGGGTCCGATTACAAAGACCATGATGGCGATTATAAGG
ACCACGACATCGATTATAAAGATGACGATGATAAATAGGTCGAGCCGCGGCAATTCCGACGTTACTGGCCGAAGCCGCTTGGAATAAGGCCGGTGTGCG
TTTGTCTATATGTTATTTTCCACCATATTGCCGTCTTTTGGCAATGTGAGGGCCCGGAAACCTGGCCCTGTCTTCTTGACGAGCATTCCTAGGGGTCTTTCCC
CTCTCGCCAAAGGAATGCAAGGTCTGTTGAATGTCGTGAAGGAAGCAGTTCCTCTGGAAGCTTCTTGAAGACAAACAACGTCTGTAGCGACCCTTTGCAG
GCAGCGGAACCCCCCACCTGGCAACAGGTGCCTCTGCGGCCAAAAGCCACGTGTATAAGATACACCTGCAAAGGCGGCACAACCCCAGTGCCACGTTGT
GAGTTGGATAGTTGTGGAAAGAGTCAAATGGCTCTCCTCAAGCGTATTCAACAAGGGGCTGAAGGATGCCCAGAAGGTACCCCATTGTATGGGATCTGA
TCTGGGGCCTCGGTGCACATGCTTTACATGTGTTTAGTCGAGGTTAAAAAAACGTCTAGGCCCCCCGAACCACGGGGACGTGGTTTTCCTTTGAAAAACAC
GATGATAATATGGTGTCAGTGATTAAGCCCGAGATGAAAATGAGATATTATATGGACGGGAGCGTCAATGGGCATGAGTTCACTATCGAAGGCGAGGGA
ACGGGACGCCCTTATGAGGGGCACCAAGAAATGACACTCAGAGTAACTATGGCAGAAGGCGGTCCAATGCCATTTGCATTCGATCTGGTTAGCCACGTAT
TTTGCTACGGGCATAGAGTTTTTACAAAATACCCCGAAGAGATCCCAGATTACTTTAAGCAGGCATTCCCTGAAGGCCTGTCCTGGGAACGATCCCTGGAG
TTCGAGGACGGGGGCAGCGCGAGTGTCTCCGCTCACATTAGTCTGAGGGGGAACACCTTTTATCATAAATCTAAATTCACAGGAGTCAATTTCCCAGCAG
ATGGCCCAATTATGCAGAATCAGTCAGTTGATTGGGAGCCTTCTACGGAAAAGATCACTGCAAGCGACGGCGTGCTTAAGGGGGATGTCACCATGTATCT
GAAACTGGAAGGGGGCGGAAACCATAAGTGTCAAATGAAAACTACCTATAAGGCCGCAAAGGAGATTCTCGAGATGCCCGGCGATCATTACATCGGTCA
TCGATTAGTTCGTAAGACCGAGGGAAATATCACTGAACAGGTCGAGGACGCTGTTGCACACTCATGAGCTAGCTTGACTGACTGAGATACAGCGTACCTT
CAGCTCACAGACATGATAAGATACATTGATGAGTTTGGACAAACCACAACTAGAATGCAGTGAAAAAAATGCTTTATTTGTGAAATTTGTGATGCTATTGC
TTTATTTGTAACCATTATAAGCTGCAATAAACAAGTTAACAACAACAATTGCATTCATTTTATGTTTCAGGTTCAGGGGGAGGTGTGGGAGGTTTTTTAAAG
CAAGTAAAACCTCTACAAATGTGGTATTGGCCCATCTCTATCGGTATCGTAGCATAACCCCTTGGGGCCTCTAAACGGGTCTTGAGGGGTTTTTTGTGCCCC
TCGGGCCGGATTGCTATCTACCGGCATTGGCGCAGAAAAAAATGCCTGATGCGACGCTGCGCGTCTTATACTCCCACATATGCCAGATTCAGCAACGGAT
ACGGCTTCCCCAACTTGCCCACTTCCATACGTGTCCTCCTTACCAGAAATTTATCCTTAAGGTCGTCAGCTATCCTGCAGGCGATCTCTCGATTTCGATCAAG
ACATTCCTTTAATGGTCTTTTCTGGACACCACTAGGGGTCAGAAGTAGTTCATCAAACTTTCTTCCCTCCCTAATCTCATTGGTTACCTTGGGCTATCGAAAC
TTAATTAACCAGTCAAGTCAGCTACTTGGCGAGATCGACTTGTCTGGGTTTCGACTACGCTCAGAATTGCGTCAGTCAAGTTCGATCTGGTCCTTGCTATTG
CACCCGTTCTCCGATTACGAGTTTCATTTAAATCATGTGAGCAAAAGGCCAGCAAAAGGCCAGGAACCGTAAAAAGGCCGCGTTGCTGGCGTTTTTCCATA
GGCTCCGCCCCCCTGACGAGCATCACAAAAATCGACGCTCAAGTCAGAGGTGGCGAAACCCGACAGGACTATAAAGATACCAGGCGTTTCCCCCTGGAA
GCTCCCTCGTGCGCTCTCCTGTTCCGACCCTGCCGCTTACCGGATACCTGTCCGCCTTTCTCCCTTCGGGAAGCGTGGCGCTTTCTCATAGCTCACGCTGTA
GGTATCTCAGTTCGGTGTAGGTCGTTCGCTCCAAGCTGGGCTGTGTGCACGAACCCCCCGTTCAGCCCGACCGCTGCGCCTTATCCGGTAACTATCGTCTT
GAGTCCAACCCGGTAAGACACGACTTATCGCCACTGGCAGCAGCCACTGGTAACAGGATTAGCAGAGCGAGGTATGTAGGCGGTGCTACAGAGTTCTTG
AAGTGGTGGCCTAACTACGGCTACACTAGAAGAACAGTATTTGGTATCTGCGCTCTGCTGAAGCCAGTTACCTTCGGAAAAAGAGTTGGTAGCTCTTGAT
CCGGCAAACAAACCACCGCTGGTAGCGGTGGTTTTTTTGTTTGCAAGCAGCAGATTACGCGCAGAAAAAAAGGATCTCAAGAAGATCCTTTGATCTTTTCT
ACGGGGTCTGACGCTCAGTGGAACGAAAACTCACGTTAAGGGATTTTGGTCATGAGATTATCAAAAAGGATCTTCACCTAGATCCTTTTAAATTAAAAATG
AAGTTTTAAATCAATCTAAAGTATATATGAGTAAACTTGGTCTGACAGTTACCAATGCTTAATCAGTGAGGCACCTATCTCAGCGATCTGTCTATTTCGTTC
ATCCATAGTTGCATTTAAATTTCCGAACTCTCCAAGGCCCTCGTCGGAAAATCTTCAAACCTTTCGTCCGATCCATCTTGCAGGCTACCTCTCGAACGAACTA
TCGCAAGTCTCTTGGCCGGCCTTGCGCCTTGGCTATTGCTTGGCAGCGCCTATCGCCAGGTATTACTCCAATCCCGAATATCCGAGATCGGGATCACCCGA
GAGAAGTTCAACCTACATCCTCAATCCCGATCTATCCGAGATCCGAGGAATATCGAAATCGGGGCGCGCCTGGCCTCCGCGCCGGGTTTTGGCGCCTCCC
GCGGGCGCCCCCCTCGTCACGGCGAGCGCTGCCACGTCAGACGAAGGGCGCAGGAGCGTCCTGATCCTTCCGCCCGGACGCTCAGGACAGCGGCCCGCT
GCTCATAAGACTCGGCCTTAGAACCCCAGTATCAGCAGAAGGACATTTTAGGACGGGACTTGGGTGACTCTAGGGCACTGGTTTTCTTTCCAGAGAGCGG
AACAGGCGAGGAAAAGTAGTCCCTTCTCGGCGATTCTGCGGAGGGATCTCCGTGGGGCGGTGAACGCCGATGATTATATAAGGACGCGCCGGGTGTGG

CACAGCTAGTTCCGTCGCAGCCGGGATTTGGGTCGCGGTTCTTGTTTGTGGATCGCTGTGATCGTCACTTGGTGAGTAGCGGGCTGCTGGGCTGGCCGGG
GCTTTCGTGGCCGCCGGGCCGCTCGGTGGGACGGAAGCGTGTGGAGAGACCGCCAAGGGCTGTAGTCTGGGTCCGCGAGCAAGGTTGCCCTGAACTGG
GGGTTGGGGGGAGCGCAGCAAAATGGCGGCTGTTCCCGAGTCTTGAATGGAAGACGCTTGTGAGGCGGGCTGTGAGGTCGTTGAAACAAGGTGGGGG
GCATGGTGGGCGGCAAGAACCCAAGGTCTTGAGCCCTTCGCTAATGCGGGAAAGCTCTTATTCGGGTGAGATGGGCTGGGCACCATCTGGGGACCCTGA
CGTGAAGTTTGTCACTGACTGGAGAACTCGGTTTGTCGTCTGTTGCGGGGGCGGCAGTTATGGCGGTGCCGTTGGGCAGTGCACCCGTACCTTTGGGAG
CGCGCGCCCTCGTCGTGTCGTGACGTCACCCGTTCTGTTGGCTTATAATGCAGGGTGGGGCCACCTGCCGGTAGGTGTGCGGTAGGCTTTTCTCCGTCGC
AGGACGCAGGGTTCGGGCCTAGGGTAGGCTCTCCTGAATCGACAGGCGCCGGACCTCTGGTGAGGGGAGGGATAAGTGAGGCGTCAGTTTCTTTGGTC
GGTTTTATGTACCTATCTTCTTAAGTAGCTGAAGCTCCGGTTTTGAACTATGCGCTCGGGGTTGGCGAGTGTGTTTTGTGAAGTTTTTTAGGCACCTTTTGA
AATGTAATCATTTGGGTCAATATGTAATTTTCAGTGTTAGACTTGTAAATTGTCCGCTAAATTCTGGCCGTTTTTGGCTTTTTTGTTAGACAACATGACCGAG
TACAAGCCCACGGTGCGCCTCGCCACCCGCGACGACGTCCCCAGGGCCGTACGCACCCTCGCCGCCGCGTTCGCCGACTACCCCGCCACGCGCCACACCG
TCGATCCGGACCGCCACATCGAGCGGGTCACCGAGCTGCAAGAACTCTTCCTCACGCGCGTCGGGCTCGACATCGGCAAGGTGTGGGTCGCGGACGACG
GCGCCGCGGTGGCGGTCTGGACCACGCCGGAGAGCGTCGAAGCGGGGGCGGTGTTCGCCGAGATCGGCCCGCGCATGGCCGAGTTGAGCGGTTCCCG
GCTGGCCGCGCAGCAACAGATGGAGGGCCTCCTGGCGCCGCACCGGCCCAAGGAGCCCGCGTGGTTCCTGGCCACCGTCGGCGTCTCGCCCGACCACCA
GGGCAAGGGTCTGGGCAGCGCCGTCGTGCTCCCCGGAGTGGAGGCCGCCGAGCGCGCCGGGGTGCCCGCCTTCCTGGAGACCTCCGCGCCCCGCAACC
TCCCCTTCTACGAGCGGCTCGGCTTCACCGTCACCGCCGACGTCGAGGTGCCCGAAGGACCGCGCACCTGGTGCATGACCCGCAAGCCCGGTGCCTAGGC
TAGTATGTAAGCCTAGTCTTAGATAATAAAATCGCTATCCATCGAAGATGGATGTGTGTTGGTTTTTTGTGTGTGTAACGCTAGGCGCGCCTGGTGTACCG
AGAACGATCCTCTCAGTGCGAGTCTCGACGATCCATATCGTTGCTTGGCAGTCAGCCAGTCGGAATCCAGCTTGGGACCCAGGAAGTCCAATCGTCAGAT
ATTGTACTCAAGCCTGGTCACGGCAGCGTACCGATCTGTTTAAACCTAGATATTGATAGTCTGATCGGTCAACGTATAATCGAGTCCTAGCTTTTGCAAACA
TCTATCAAGAGACAGGATCAGCAGGAGGCTTTCGCATGAGTATTCAACATTTCCGTGTCGCCCTTATTCCCTTTTTTGCGGCATTTTGCCTTCCTGTTTTTGC
TCACCCAGAAACGCTGGTGAAAGTAAAAGATGCTGAAGATCAGTTGGGTGCGCGAGTGGGTTACATCGAACTGGATCTCAACAGCGGTAAGATCCTTGA
GAGTTTTCGCCCCGAAGAACGCTTTCCAATGATGAGCACTTTTAAAGTTCTGCTATGTGGCGCGGTATTATCCCGTATTGACGCCGGGCAAGAGCAACTCG
GTCGCCGCATACACTATTCTCAGAATGACTTGGTTGAGTATTCACCAGTCACAGAAAAGCATCTTACGGATGGCATGACAGTAAGAGAATTATGCAGTGCT
GCCATAACCATGAGTGATAACACTGCGGCCAACTTACTTCTGACAACGATTGGAGGACCGAAGGAGCTAACCGCTTTTTTGCACAACATGGGGGATCATG
TAACTCGCCTTGATCGTTGGGAACCGGAGCTGAATGAAGCCATACCAAACGACGAGCGTGACACCACGATGCCTGTAGCAATGGCAACAACCTTGCGTAA
ACTATTAACTGGCGAACTACTTACTCTAGCTTCCCGGCAACAGTTGATAGACTGGATGGAGGCGGATAAAGTTGCAGGACCACTTCTGCGCTCGGCCCTTC
CGGCTGGCTGGTTTATTGCTGATAAATCTGGAGCCGGTGAGCGTGGGTCTCGCGGTATCATTGCAGCACTGGGGCCAGATGGTAAGCCCTCCCGTATCGT
AGTTATCTACACGACGGGGAGTCAGGCAACTATGGATGAACGAAATAGACAGATCGCTGAGATAGGTGCCTCACTGATTAAGCATTGGTAACCGATTCTA
GGTGCATTGGCGCAGAAAAAAATGCCTGATGCGACGCTGCGCGTCTTATACTCCCACATATGCCAGATTCAGCAACGGATACGGCTTCCCCAACTTGCCCA
CTTCCATACGTGTCCTCCTTACCAGAAATTTATCCTTAAGATCGTTTAAACTCGACTCTGGCTCTATCGAATCTCCGTCGTTTCGAGCTTACGCGAACAGCCG
TGGCGCTCATTTGCTCGTCGGGCATCGAATCTCGTCAGCTATCGTCAGCTTACCTTTTTGGCAGCGATCGCGGCTCCCGACATCTTGGACCATTAGCTCCAC
AGGTATCTTCTTCCCTCTAGTGGTCATAACAGCAGCTTCAGCTACCTCTCAATTCAAAAAACCCCTCAAGACCCGTTTAGAGGCCCCAAGGGGTTATGCTAT
CAATCGTTGCGTTACACACACAAAAAACCAACACACATCCATCTTCGATGGATAGCGATTTTATTATCTAACTGCTGATCGAGTGTAGCCAGATCTAGTAAT
CAATTACGGGGTCATTAGTTCATAGCCCATATATGGAGTTCCGCGTTACATAACTTACGGTAAATGGCCCGCCTGGCTGACCGCCCAACGACCCCCGCCCA
TTGACGTCAATAATGACGTATGTTCCCATAGTAACGCCAATAGGGACTTTCCATTGACGTCAATGGGTGGAGTATTTACGGTAAACTGCCCACTTGGCAGT
ACATCAAGTGTATCATATGCCAAGTACGCCCCCTATTGACGTCAATGACGGTAAATGGCCCGCCTGGCATTATGCCCAGTACATGACCTTATGGGACTTTC
CTACTTGGCAGTACATCTACGTATTAGTCATCGCTATTACCATGCTGATGCGGTTTTGGCAGTACATCAATGGGCGTGGATAGCGGTTTGACTCACGGGGA
TTTCCAAGTCTCCACCCCATTGACGTCAATGGGAGTTTGTTTTGGCACCAAAATCAACGGGACTTTCCAAAATGTCGTAACAACTCCGCCCCATTGACGCAA
ATGGGCGGTAGGCGTGTACGGTGGGAGGTCTATATAAGCAGAGCTGGTTTAGTGAACCGTCAGATCAGATCTTTGTCGATCCTACCATCCACTCGACACA
CCCGCCAGCGGCCGCATGTACCCGTACGATGTCCCCGACTACGCCGGATCAGGCTCT

Q9NYX4

GTAAAACTGGGGTGTTCATTCAGTGGGAAGCCCGGCAAGGATCCGGGTGACCAGGATGGCGCCGCTATGGATAGCGTCCCCCTGATCTCTCCGCTTGATA
TTTCTCAATTACAGCCCCCGCTTCCGGATCAGGTGGTGATCAAGACTCAGACGGAATACCAGTTAAGTTCTCCGGACCAGCAGAACTTTCCCGATTTGGAA
GGGCAGCGACTTAACTGCTCCCACCCAGAGGAGGGCAGGCGCCTGCCCACGGCACGGATGATCGCCTTCGCTATGGCCCTGCTGGGGTGCGTACTGATA
ATGTATAAAGCTATTTGGTATGACCAATTTACTTGTCCGGACGGATTCCTCTTAAGACACAAAATCTGTACACCACTCACACTGGAGATGTACTACACGGA
GATGGATCCTGAACGGCACCGTTCCATCCTCGCTGCCATCGGAGCTTACCCCCTCTCCCGAAAACATGGCACTGAGACACCCGCAGCCTGGGGCGATGGG
TATAGAGCTGCCAAGGAGGAAAGGAAAGGTCCAACTCAGGCGGGAGCCGCTGCTGCCGCCACAGAGCCGCCAGGGAAGCCGAGTGCTAAGGCAGAGA
AAGAGGCGGCGAGGAAGGCAGCGGGGTCAGCTGCCCCCCCACCGGCCCAAGGCAGTGGGTCCGATTACAAAGACCATGATGGCGATTATAAGGACCAC
GACATCGATTATAAAGATGACGATGATAAATAGGTCGAGCCGCGGCAATTCCGACGTTACTGGCCGAAGCCGCTTGGAATAAGGCCGGTGTGCGTTTGTC
TATATGTTATTTTCCACCATATTGCCGTCTTTTGGCAATGTGAGGGCCCGGAAACCTGGCCCTGTCTTCTTGACGAGCATTCCTAGGGGTCTTTCCCCTCTCG
CCAAAGGAATGCAAGGTCTGTTGAATGTCGTGAAGGAAGCAGTTCCTCTGGAAGCTTCTTGAAGACAAACAACGTCTGTAGCGACCCTTTGCAGGCAGCG
GAACCCCCCACCTGGCAACAGGTGCCTCTGCGGCCAAAAGCCACGTGTATAAGATACACCTGCAAAGGCGGCACAACCCCAGTGCCACGTTGTGAGTTGG
ATAGTTGTGGAAAGAGTCAAATGGCTCTCCTCAAGCGTATTCAACAAGGGGCTGAAGGATGCCCAGAAGGTACCCCATTGTATGGGATCTGATCTGGGG
CCTCGGTGCACATGCTTTACATGTGTTTAGTCGAGGTTAAAAAAACGTCTAGGCCCCCCGAACCACGGGGACGTGGTTTTCCTTTGAAAAACACGATGATA
ATATGGTGTCAGTGATTAAGCCCGAGATGAAAATGAGATATTATATGGACGGGAGCGTCAATGGGCATGAGTTCACTATCGAAGGCGAGGGAACGGGA
CGCCCTTATGAGGGGCACCAAGAAATGACACTCAGAGTAACTATGGCAGAAGGCGGTCCAATGCCATTTGCATTCGATCTGGTTAGCCACGTATTTTGCTA
CGGGCATAGAGTTTTTACAAAATACCCCGAAGAGATCCCAGATTACTTTAAGCAGGCATTCCCTGAAGGCCTGTCCTGGGAACGATCCCTGGAGTTCGAG
GACGGGGGCAGCGCGAGTGTCTCCGCTCACATTAGTCTGAGGGGGAACACCTTTTATCATAAATCTAAATTCACAGGAGTCAATTTCCCAGCAGATGGCC
CAATTATGCAGAATCAGTCAGTTGATTGGGAGCCTTCTACGGAAAAGATCACTGCAAGCGACGGCGTGCTTAAGGGGGATGTCACCATGTATCTGAAACT
GGAAGGGGGCGGAAACCATAAGTGTCAAATGAAAACTACCTATAAGGCCGCAAAGGAGATTCTCGAGATGCCCGGCGATCATTACATCGGTCATCGATT
AGTTCGTAAGACCGAGGGAAATATCACTGAACAGGTCGAGGACGCTGTTGCACACTCATGAGCTAGCTTGACTGACTGAGATACAGCGTACCTTCAGCTC
ACAGACATGATAAGATACATTGATGAGTTTGGACAAACCACAACTAGAATGCAGTGAAAAAAATGCTTTATTTGTGAAATTTGTGATGCTATTGCTTTATTT
GTAACCATTATAAGCTGCAATAAACAAGTTAACAACAACAATTGCATTCATTTTATGTTTCAGGTTCAGGGGGAGGTGTGGGAGGTTTTTTAAAGCAAGTA
AAACCTCTACAAATGTGGTATTGGCCCATCTCTATCGGTATCGTAGCATAACCCCTTGGGGCCTCTAAACGGGTCTTGAGGGGTTTTTTGTGCCCCTCGGG
CCGGATTGCTATCTACCGGCATTGGCGCAGAAAAAAATGCCTGATGCGACGCTGCGCGTCTTATACTCCCACATATGCCAGATTCAGCAACGGATACGGCT
TCCCCAACTTGCCCACTTCCATACGTGTCCTCCTTACCAGAAATTTATCCTTAAGGTCGTCAGCTATCCTGCAGGCGATCTCTCGATTTCGATCAAGACATTC
CTTTAATGGTCTTTTCTGGACACCACTAGGGGTCAGAAGTAGTTCATCAAACTTTCTTCCCTCCCTAATCTCATTGGTTACCTTGGGCTATCGAAACTTAATT
AACCAGTCAAGTCAGCTACTTGGCGAGATCGACTTGTCTGGGTTTCGACTACGCTCAGAATTGCGTCAGTCAAGTTCGATCTGGTCCTTGCTATTGCACCC

GTTCTCCGATTACGAGTTTCATTTAAATCATGTGAGCAAAAGGCCAGCAAAAGGCCAGGAACCGTAAAAAGGCCGCGTTGCTGGCGTTTTTCCATAGGCT
CCGCCCCCCTGACGAGCATCACAAAAATCGACGCTCAAGTCAGAGGTGGCGAAACCCGACAGGACTATAAAGATACCAGGCGTTTCCCCCTGGAAGCTCC
CTCGTGCGCTCTCCTGTTCCGACCCTGCCGCTTACCGGATACCTGTCCGCCTTTCTCCCTTCGGGAAGCGTGGCGCTTTCTCATAGCTCACGCTGTAGGTAT
CTCAGTTCGGTGTAGGTCGTTCGCTCCAAGCTGGGCTGTGTGCACGAACCCCCCGTTCAGCCCGACCGCTGCGCCTTATCCGGTAACTATCGTCTTGAGTC
CAACCCGGTAAGACACGACTTATCGCCACTGGCAGCAGCCACTGGTAACAGGATTAGCAGAGCGAGGTATGTAGGCGGTGCTACAGAGTTCTTGAAGTG
GTGGCCTAACTACGGCTACACTAGAAGAACAGTATTTGGTATCTGCGCTCTGCTGAAGCCAGTTACCTTCGGAAAAAGAGTTGGTAGCTCTTGATCCGGC
AAACAAACCACCGCTGGTAGCGGTGGTTTTTTTGTTTGCAAGCAGCAGATTACGCGCAGAAAAAAAGGATCTCAAGAAGATCCTTTGATCTTTTCTACGGG
GTCTGACGCTCAGTGGAACGAAAACTCACGTTAAGGGATTTTGGTCATGAGATTATCAAAAAGGATCTTCACCTAGATCCTTTTAAATTAAAAATGAAGTT
TTAAATCAATCTAAAGTATATATGAGTAAACTTGGTCTGACAGTTACCAATGCTTAATCAGTGAGGCACCTATCTCAGCGATCTGTCTATTTCGTTCATCCAT
AGTTGCATTTAAATTTCCGAACTCTCCAAGGCCCTCGTCGGAAAATCTTCAAACCTTTCGTCCGATCCATCTTGCAGGCTACCTCTCGAACGAACTATCGCA
AGTCTCTTGGCCGGCCTTGCGCCTTGGCTATTGCTTGGCAGCGCCTATCGCCAGGTATTACTCCAATCCCGAATATCCGAGATCGGGATCACCCGAGAGAA
GTTCAACCTACATCCTCAATCCCGATCTATCCGAGATCCGAGGAATATCGAAATCGGGGCGCGCCTGGCCTCCGCGCCGGGTTTTGGCGCCTCCCGCGGG
CGCCCCCCTCGTCACGGCGAGCGCTGCCACGTCAGACGAAGGGCGCAGGAGCGTCCTGATCCTTCCGCCCGGACGCTCAGGACAGCGGCCCGCTGCTCA
TAAGACTCGGCCTTAGAACCCCAGTATCAGCAGAAGGACATTTTAGGACGGGACTTGGGTGACTCTAGGGCACTGGTTTTCTTTCCAGAGAGCGGAACAG
GCGAGGAAAAGTAGTCCCTTCTCGGCGATTCTGCGGAGGGATCTCCGTGGGGCGGTGAACGCCGATGATTATATAAGGACGCGCCGGGTGTGGCACAG
CTAGTTCCGTCGCAGCCGGGATTTGGGTCGCGGTTCTTGTTTGTGGATCGCTGTGATCGTCACTTGGTGAGTAGCGGGCTGCTGGGCTGGCCGGGGCTTT
CGTGGCCGCCGGGCCGCTCGGTGGGACGGAAGCGTGTGGAGAGACCGCCAAGGGCTGTAGTCTGGGTCCGCGAGCAAGGTTGCCCTGAACTGGGGGTT
GGGGGGAGCGCAGCAAAATGGCGGCTGTTCCCGAGTCTTGAATGGAAGACGCTTGTGAGGCGGGCTGTGAGGTCGTTGAAACAAGGTGGGGGGCATG
GTGGGCGGCAAGAACCCAAGGTCTTGAGCCCTTCGCTAATGCGGGAAAGCTCTTATTCGGGTGAGATGGGCTGGGCACCATCTGGGGACCCTGACGTGA
AGTTTGTCACTGACTGGAGAACTCGGTTTGTCGTCTGTTGCGGGGGCGGCAGTTATGGCGGTGCCGTTGGGCAGTGCACCCGTACCTTTGGGAGCGCGC
GCCCTCGTCGTGTCGTGACGTCACCCGTTCTGTTGGCTTATAATGCAGGGTGGGGCCACCTGCCGGTAGGTGTGCGGTAGGCTTTTCTCCGTCGCAGGAC
GCAGGGTTCGGGCCTAGGGTAGGCTCTCCTGAATCGACAGGCGCCGGACCTCTGGTGAGGGGAGGGATAAGTGAGGCGTCAGTTTCTTTGGTCGGTTTT
ATGTACCTATCTTCTTAAGTAGCTGAAGCTCCGGTTTTGAACTATGCGCTCGGGGTTGGCGAGTGTGTTTTGTGAAGTTTTTTAGGCACCTTTTGAAATGTA
ATCATTTGGGTCAATATGTAATTTTCAGTGTTAGACTTGTAAATTGTCCGCTAAATTCTGGCCGTTTTTGGCTTTTTTGTTAGACAACATGACCGAGTACAAG
CCCACGGTGCGCCTCGCCACCCGCGACGACGTCCCCAGGGCCGTACGCACCCTCGCCGCCGCGTTCGCCGACTACCCCGCCACGCGCCACACCGTCGATC
CGGACCGCCACATCGAGCGGGTCACCGAGCTGCAAGAACTCTTCCTCACGCGCGTCGGGCTCGACATCGGCAAGGTGTGGGTCGCGGACGACGGCGCC
GCGGTGGCGGTCTGGACCACGCCGGAGAGCGTCGAAGCGGGGGCGGTGTTCGCCGAGATCGGCCCGCGCATGGCCGAGTTGAGCGGTTCCCGGCTGG
CCGCGCAGCAACAGATGGAGGGCCTCCTGGCGCCGCACCGGCCCAAGGAGCCCGCGTGGTTCCTGGCCACCGTCGGCGTCTCGCCCGACCACCAGGGCA
AGGGTCTGGGCAGCGCCGTCGTGCTCCCCGGAGTGGAGGCCGCCGAGCGCGCCGGGGTGCCCGCCTTCCTGGAGACCTCCGCGCCCCGCAACCTCCCCT
TCTACGAGCGGCTCGGCTTCACCGTCACCGCCGACGTCGAGGTGCCCGAAGGACCGCGCACCTGGTGCATGACCCGCAAGCCCGGTGCCTAGGCTAGTA
TGTAAGCCTAGTCTTAGATAATAAAATCGCTATCCATCGAAGATGGATGTGTGTTGGTTTTTTGTGTGTGTAACGCTAGGCGCGCCTGGTGTACCGAGAAC
GATCCTCTCAGTGCGAGTCTCGACGATCCATATCGTTGCTTGGCAGTCAGCCAGTCGGAATCCAGCTTGGGACCCAGGAAGTCCAATCGTCAGATATTGTA
CTCAAGCCTGGTCACGGCAGCGTACCGATCTGTTTAAACCTAGATATTGATAGTCTGATCGGTCAACGTATAATCGAGTCCTAGCTTTTGCAAACATCTATC
AAGAGACAGGATCAGCAGGAGGCTTTCGCATGAGTATTCAACATTTCCGTGTCGCCCTTATTCCCTTTTTTGCGGCATTTTGCCTTCCTGTTTTTGCTCACCC
AGAAACGCTGGTGAAAGTAAAAGATGCTGAAGATCAGTTGGGTGCGCGAGTGGGTTACATCGAACTGGATCTCAACAGCGGTAAGATCCTTGAGAGTTT
TCGCCCCGAAGAACGCTTTCCAATGATGAGCACTTTTAAAGTTCTGCTATGTGGCGCGGTATTATCCCGTATTGACGCCGGGCAAGAGCAACTCGGTCGCC
GCATACACTATTCTCAGAATGACTTGGTTGAGTATTCACCAGTCACAGAAAAGCATCTTACGGATGGCATGACAGTAAGAGAATTATGCAGTGCTGCCATA
ACCATGAGTGATAACACTGCGGCCAACTTACTTCTGACAACGATTGGAGGACCGAAGGAGCTAACCGCTTTTTTGCACAACATGGGGGATCATGTAACTC
GCCTTGATCGTTGGGAACCGGAGCTGAATGAAGCCATACCAAACGACGAGCGTGACACCACGATGCCTGTAGCAATGGCAACAACCTTGCGTAAACTATT
AACTGGCGAACTACTTACTCTAGCTTCCCGGCAACAGTTGATAGACTGGATGGAGGCGGATAAAGTTGCAGGACCACTTCTGCGCTCGGCCCTTCCGGCT
GGCTGGTTTATTGCTGATAAATCTGGAGCCGGTGAGCGTGGGTCTCGCGGTATCATTGCAGCACTGGGGCCAGATGGTAAGCCCTCCCGTATCGTAGTTA
TCTACACGACGGGGAGTCAGGCAACTATGGATGAACGAAATAGACAGATCGCTGAGATAGGTGCCTCACTGATTAAGCATTGGTAACCGATTCTAGGTG
CATTGGCGCAGAAAAAAATGCCTGATGCGACGCTGCGCGTCTTATACTCCCACATATGCCAGATTCAGCAACGGATACGGCTTCCCCAACTTGCCCACTTC
CATACGTGTCCTCCTTACCAGAAATTTATCCTTAAGATCGTTTAAACTCGACTCTGGCTCTATCGAATCTCCGTCGTTTCGAGCTTACGCGAACAGCCGTGG
CGCTCATTTGCTCGTCGGGCATCGAATCTCGTCAGCTATCGTCAGCTTACCTTTTTGGCAGCGATCGCGGCTCCCGACATCTTGGACCATTAGCTCCACAGG
TATCTTCTTCCCTCTAGTGGTCATAACAGCAGCTTCAGCTACCTCTCAATTCAAAAAACCCCTCAAGACCCGTTTAGAGGCCCCAAGGGGTTATGCTATCAA
TCGTTGCGTTACACACACAAAAAACCAACACACATCCATCTTCGATGGATAGCGATTTTATTATCTAACTGCTGATCGAGTGTAGCCAGATCTAGTAATCAA
TTACGGGGTCATTAGTTCATAGCCCATATATGGAGTTCCGCGTTACATAACTTACGGTAAATGGCCCGCCTGGCTGACCGCCCAACGACCCCCGCCCATTG
ACGTCAATAATGACGTATGTTCCCATAGTAACGCCAATAGGGACTTTCCATTGACGTCAATGGGTGGAGTATTTACGGTAAACTGCCCACTTGGCAGTACA
TCAAGTGTATCATATGCCAAGTACGCCCCCTATTGACGTCAATGACGGTAAATGGCCCGCCTGGCATTATGCCCAGTACATGACCTTATGGGACTTTCCTAC
TTGGCAGTACATCTACGTATTAGTCATCGCTATTACCATGCTGATGCGGTTTTGGCAGTACATCAATGGGCGTGGATAGCGGTTTGACTCACGGGGATTTC
CAAGTCTCCACCCCATTGACGTCAATGGGAGTTTGTTTTGGCACCAAAATCAACGGGACTTTCCAAAATGTCGTAACAACTCCGCCCCATTGACGCAAATG
GGCGGTAGGCGTGTACGGTGGGAGGTCTATATAAGCAGAGCTGGTTTAGTGAACCGTCAGATCAGATCTTTGTCGATCCTACCATCCACTCGACACACCC
GCCAGCGGCCGCATGTACCCGTACGATGTCCCCGACTACGCCGGATCAGGCTCT

P54652

TCAGCCCGGGGCCCAGCTATTGGTATTGATCTGGGCACAACTTACAGCTGCGTCGGTGTTTTTCAGCACGGTAAGGTCGAAATAATCGCTAATGATCAGG
GTAACAGAACAACCCCGTCTTATGTGGCTTTTACCGATACAGAGCGCCTGATTGGTGACGCCGCCAAAAATCAAGTGGCAATGAACCCTACTAATACCATT
TTCGATGCTAAGCGCCTGATTGGCAGAAAATTCGAAGACGCCACAGTCCAGAGCGATATGAAACATTGGCCTTTTCGGGTCGTGTCCGAAGGGGGAAAG
CCAAAAGTGCAGGTAGAATATAAGGGGGAAACTAAGACGTTTTTCCCTGAGGAGATCTCCAGTATGGTGCTGACAAAGATGAAGGAGATTGCCGAGGCC
TATCTGGGGGGAAAGGTGCATTCAGCAGTCATCACCGTCCCTGCGTACTTTAATGATAGTCAGAGGCAAGCAACTAAAGACGCCGGAACTATCACAGGTC
TTAATGTGCTGAGGATCATTAACGAGCCCACCGCGGCAGCTATAGCCTATGGGCTCGATAAGAAGGGTTGTGCGGGCGGTGAAAAAAACGTACTCATCTT
CGACCTGGGGGGAGGCACATTTGATGTGTCAATTCTGACTATTGAGGACGGAATCTTCGAGGTGAAAAGTACAGCCGGAGACACCCATTTGGGGGGGG
AAGATTTCGACAATAGGATGGTGTCCCATTTGGCAGAGGAATTCAAGCGTAAACATAAGAAGGACATTGGTCCAAATAAGCGTGCCGTTAGGCGGCTGC
GCACCGCCTGTGAGCGAGCCAAAAGAACACTGTCTTCTTCCACTCAGGCCAGTATCGAGATTGATAGCCTGTACGAAGGAGTCGACTTCTACACCTCCATT
ACGCGTGCCCGCTTTGAGGAACTGAACGCTGATTTGTTCAGAGGGACTCTCGAGCCCGTAGAGAAGGCCCTTCGGGACGCTAAGCTGGATAAAGGGCAG
ATCCAGGAGATTGTCTTGGTGGGCGGCAGTACTCGAATACCCAAAATCCAAAAACTGCTTCAGGATTTTTTCAACGGCAAGGAGCTGAACAAGTCCATTA
ACCCCGACGAAGCCGTAGCTTACGGAGCCGCTGTCCAAGCCGCAATTCTGATCGGCGACAAGTCTGAAAACGTGCAGGATCTGCTCCTCCTCGACGTGAC

ACCACTGAGCCTGGGTATTGAAACCGCTGGTGGCGTAATGACCCCCCTGATTAAGAGAAACACAACAATCCCCACAAAACAAACTCAAACATTCACAACAT
ATAGCGACAATCAGTCTTCTGTGTTAGTGCAGGTGTATGAAGGGGAGAGAGCCATGACCAAGGACAACAACTTACTCGGGAAATTTGACCTCACGGGCA
TCCCCCCCGCTCCGCGGGGGGTCCCTCAGATTGAGGTGACTTTTGACATAGACGCGAACGGTATTCTCAATGTGACGGCCGCTGATAAGAGCACCGGCAA
GGGCAGTGGGTCCGATTACAAAGACCATGATGGCGATTATAAGGACCACGACATCGATTATAAAGATGACGATGATAAATAGGTCGAGCCGCGGCAATT
CCGACGTTACTGGCCGAAGCCGCTTGGAATAAGGCCGGTGTGCGTTTGTCTATATGTTATTTTCCACCATATTGCCGTCTTTTGGCAATGTGAGGGCCCGG
AAACCTGGCCCTGTCTTCTTGACGAGCATTCCTAGGGGTCTTTCCCCTCTCGCCAAAGGAATGCAAGGTCTGTTGAATGTCGTGAAGGAAGCAGTTCCTCT
GGAAGCTTCTTGAAGACAAACAACGTCTGTAGCGACCCTTTGCAGGCAGCGGAACCCCCCACCTGGCAACAGGTGCCTCTGCGGCCAAAAGCCACGTGTA
TAAGATACACCTGCAAAGGCGGCACAACCCCAGTGCCACGTTGTGAGTTGGATAGTTGTGGAAAGAGTCAAATGGCTCTCCTCAAGCGTATTCAACAAGG
GGCTGAAGGATGCCCAGAAGGTACCCCATTGTATGGGATCTGATCTGGGGCCTCGGTGCACATGCTTTACATGTGTTTAGTCGAGGTTAAAAAAACGTCT
AGGCCCCCCGAACCACGGGGACGTGGTTTTCCTTTGAAAAACACGATGATAATATGGTGTCAGTGATTAAGCCCGAGATGAAAATGAGATATTATATGGA
CGGGAGCGTCAATGGGCATGAGTTCACTATCGAAGGCGAGGGAACGGGACGCCCTTATGAGGGGCACCAAGAAATGACACTCAGAGTAACTATGGCAG
AAGGCGGTCCAATGCCATTTGCATTCGATCTGGTTAGCCACGTATTTTGCTACGGGCATAGAGTTTTTACAAAATACCCCGAAGAGATCCCAGATTACTTTA
AGCAGGCATTCCCTGAAGGCCTGTCCTGGGAACGATCCCTGGAGTTCGAGGACGGGGGCAGCGCGAGTGTCTCCGCTCACATTAGTCTGAGGGGGAACA
CCTTTTATCATAAATCTAAATTCACAGGAGTCAATTTCCCAGCAGATGGCCCAATTATGCAGAATCAGTCAGTTGATTGGGAGCCTTCTACGGAAAAGATC
ACTGCAAGCGACGGCGTGCTTAAGGGGGATGTCACCATGTATCTGAAACTGGAAGGGGGCGGAAACCATAAGTGTCAAATGAAAACTACCTATAAGGCC
GCAAAGGAGATTCTCGAGATGCCCGGCGATCATTACATCGGTCATCGATTAGTTCGTAAGACCGAGGGAAATATCACTGAACAGGTCGAGGACGCTGTT
GCACACTCATGAGCTAGCTTGACTGACTGAGATACAGCGTACCTTCAGCTCACAGACATGATAAGATACATTGATGAGTTTGGACAAACCACAACTAGAAT
GCAGTGAAAAAAATGCTTTATTTGTGAAATTTGTGATGCTATTGCTTTATTTGTAACCATTATAAGCTGCAATAAACAAGTTAACAACAACAATTGCATTCA
TTTTATGTTTCAGGTTCAGGGGGAGGTGTGGGAGGTTTTTTAAAGCAAGTAAAACCTCTACAAATGTGGTATTGGCCCATCTCTATCGGTATCGTAGCATA
ACCCCTTGGGGCCTCTAAACGGGTCTTGAGGGGTTTTTTGTGCCCCTCGGGCCGGATTGCTATCTACCGGCATTGGCGCAGAAAAAAATGCCTGATGCGA
CGCTGCGCGTCTTATACTCCCACATATGCCAGATTCAGCAACGGATACGGCTTCCCCAACTTGCCCACTTCCATACGTGTCCTCCTTACCAGAAATTTATCCT
TAAGGTCGTCAGCTATCCTGCAGGCGATCTCTCGATTTCGATCAAGACATTCCTTTAATGGTCTTTTCTGGACACCACTAGGGGTCAGAAGTAGTTCATCAA
ACTTTCTTCCCTCCCTAATCTCATTGGTTACCTTGGGCTATCGAAACTTAATTAACCAGTCAAGTCAGCTACTTGGCGAGATCGACTTGTCTGGGTTTCGACT
ACGCTCAGAATTGCGTCAGTCAAGTTCGATCTGGTCCTTGCTATTGCACCCGTTCTCCGATTACGAGTTTCATTTAAATCATGTGAGCAAAAGGCCAGCAAA
AGGCCAGGAACCGTAAAAAGGCCGCGTTGCTGGCGTTTTTCCATAGGCTCCGCCCCCCTGACGAGCATCACAAAAATCGACGCTCAAGTCAGAGGTGGC
GAAACCCGACAGGACTATAAAGATACCAGGCGTTTCCCCCTGGAAGCTCCCTCGTGCGCTCTCCTGTTCCGACCCTGCCGCTTACCGGATACCTGTCCGCC
TTTCTCCCTTCGGGAAGCGTGGCGCTTTCTCATAGCTCACGCTGTAGGTATCTCAGTTCGGTGTAGGTCGTTCGCTCCAAGCTGGGCTGTGTGCACGAACC
CCCCGTTCAGCCCGACCGCTGCGCCTTATCCGGTAACTATCGTCTTGAGTCCAACCCGGTAAGACACGACTTATCGCCACTGGCAGCAGCCACTGGTAACA
GGATTAGCAGAGCGAGGTATGTAGGCGGTGCTACAGAGTTCTTGAAGTGGTGGCCTAACTACGGCTACACTAGAAGAACAGTATTTGGTATCTGCGCTCT
GCTGAAGCCAGTTACCTTCGGAAAAAGAGTTGGTAGCTCTTGATCCGGCAAACAAACCACCGCTGGTAGCGGTGGTTTTTTTGTTTGCAAGCAGCAGATT
ACGCGCAGAAAAAAAGGATCTCAAGAAGATCCTTTGATCTTTTCTACGGGGTCTGACGCTCAGTGGAACGAAAACTCACGTTAAGGGATTTTGGTCATGA
GATTATCAAAAAGGATCTTCACCTAGATCCTTTTAAATTAAAAATGAAGTTTTAAATCAATCTAAAGTATATATGAGTAAACTTGGTCTGACAGTTACCAAT
GCTTAATCAGTGAGGCACCTATCTCAGCGATCTGTCTATTTCGTTCATCCATAGTTGCATTTAAATTTCCGAACTCTCCAAGGCCCTCGTCGGAAAATCTTCA
AACCTTTCGTCCGATCCATCTTGCAGGCTACCTCTCGAACGAACTATCGCAAGTCTCTTGGCCGGCCTTGCGCCTTGGCTATTGCTTGGCAGCGCCTATCGC
CAGGTATTACTCCAATCCCGAATATCCGAGATCGGGATCACCCGAGAGAAGTTCAACCTACATCCTCAATCCCGATCTATCCGAGATCCGAGGAATATCGA
AATCGGGGCGCGCCTGGCCTCCGCGCCGGGTTTTGGCGCCTCCCGCGGGCGCCCCCCTCGTCACGGCGAGCGCTGCCACGTCAGACGAAGGGCGCAGGA
GCGTCCTGATCCTTCCGCCCGGACGCTCAGGACAGCGGCCCGCTGCTCATAAGACTCGGCCTTAGAACCCCAGTATCAGCAGAAGGACATTTTAGGACGG
GACTTGGGTGACTCTAGGGCACTGGTTTTCTTTCCAGAGAGCGGAACAGGCGAGGAAAAGTAGTCCCTTCTCGGCGATTCTGCGGAGGGATCTCCGTGG
GGCGGTGAACGCCGATGATTATATAAGGACGCGCCGGGTGTGGCACAGCTAGTTCCGTCGCAGCCGGGATTTGGGTCGCGGTTCTTGTTTGTGGATCGC
TGTGATCGTCACTTGGTGAGTAGCGGGCTGCTGGGCTGGCCGGGGCTTTCGTGGCCGCCGGGCCGCTCGGTGGGACGGAAGCGTGTGGAGAGACCGCC
AAGGGCTGTAGTCTGGGTCCGCGAGCAAGGTTGCCCTGAACTGGGGGTTGGGGGGAGCGCAGCAAAATGGCGGCTGTTCCCGAGTCTTGAATGGAAGA
CGCTTGTGAGGCGGGCTGTGAGGTCGTTGAAACAAGGTGGGGGGCATGGTGGGCGGCAAGAACCCAAGGTCTTGAGCCCTTCGCTAATGCGGGAAAGC
TCTTATTCGGGTGAGATGGGCTGGGCACCATCTGGGGACCCTGACGTGAAGTTTGTCACTGACTGGAGAACTCGGTTTGTCGTCTGTTGCGGGGGCGGC
AGTTATGGCGGTGCCGTTGGGCAGTGCACCCGTACCTTTGGGAGCGCGCGCCCTCGTCGTGTCGTGACGTCACCCGTTCTGTTGGCTTATAATGCAGGGT
GGGGCCACCTGCCGGTAGGTGTGCGGTAGGCTTTTCTCCGTCGCAGGACGCAGGGTTCGGGCCTAGGGTAGGCTCTCCTGAATCGACAGGCGCCGGACC
TCTGGTGAGGGGAGGGATAAGTGAGGCGTCAGTTTCTTTGGTCGGTTTTATGTACCTATCTTCTTAAGTAGCTGAAGCTCCGGTTTTGAACTATGCGCTCG
GGGTTGGCGAGTGTGTTTTGTGAAGTTTTTTAGGCACCTTTTGAAATGTAATCATTTGGGTCAATATGTAATTTTCAGTGTTAGACTTGTAAATTGTCCGCT
AAATTCTGGCCGTTTTTGGCTTTTTTGTTAGACAACATGACCGAGTACAAGCCCACGGTGCGCCTCGCCACCCGCGACGACGTCCCCAGGGCCGTACGCAC
CCTCGCCGCCGCGTTCGCCGACTACCCCGCCACGCGCCACACCGTCGATCCGGACCGCCACATCGAGCGGGTCACCGAGCTGCAAGAACTCTTCCTCACGC
GCGTCGGGCTCGACATCGGCAAGGTGTGGGTCGCGGACGACGGCGCCGCGGTGGCGGTCTGGACCACGCCGGAGAGCGTCGAAGCGGGGGCGGTGTT
CGCCGAGATCGGCCCGCGCATGGCCGAGTTGAGCGGTTCCCGGCTGGCCGCGCAGCAACAGATGGAGGGCCTCCTGGCGCCGCACCGGCCCAAGGAGC
CCGCGTGGTTCCTGGCCACCGTCGGCGTCTCGCCCGACCACCAGGGCAAGGGTCTGGGCAGCGCCGTCGTGCTCCCCGGAGTGGAGGCCGCCGAGCGCG
CCGGGGTGCCCGCCTTCCTGGAGACCTCCGCGCCCCGCAACCTCCCCTTCTACGAGCGGCTCGGCTTCACCGTCACCGCCGACGTCGAGGTGCCCGAAGG
ACCGCGCACCTGGTGCATGACCCGCAAGCCCGGTGCCTAGGCTAGTATGTAAGCCTAGTCTTAGATAATAAAATCGCTATCCATCGAAGATGGATGTGTG
TTGGTTTTTTGTGTGTGTAACGCTAGGCGCGCCTGGTGTACCGAGAACGATCCTCTCAGTGCGAGTCTCGACGATCCATATCGTTGCTTGGCAGTCAGCCA
GTCGGAATCCAGCTTGGGACCCAGGAAGTCCAATCGTCAGATATTGTACTCAAGCCTGGTCACGGCAGCGTACCGATCTGTTTAAACCTAGATATTGATAG
TCTGATCGGTCAACGTATAATCGAGTCCTAGCTTTTGCAAACATCTATCAAGAGACAGGATCAGCAGGAGGCTTTCGCATGAGTATTCAACATTTCCGTGT
CGCCCTTATTCCCTTTTTTGCGGCATTTTGCCTTCCTGTTTTTGCTCACCCAGAAACGCTGGTGAAAGTAAAAGATGCTGAAGATCAGTTGGGTGCGCGAGT
GGGTTACATCGAACTGGATCTCAACAGCGGTAAGATCCTTGAGAGTTTTCGCCCCGAAGAACGCTTTCCAATGATGAGCACTTTTAAAGTTCTGCTATGTG
GCGCGGTATTATCCCGTATTGACGCCGGGCAAGAGCAACTCGGTCGCCGCATACACTATTCTCAGAATGACTTGGTTGAGTATTCACCAGTCACAGAAAA
GCATCTTACGGATGGCATGACAGTAAGAGAATTATGCAGTGCTGCCATAACCATGAGTGATAACACTGCGGCCAACTTACTTCTGACAACGATTGGAGGA
CCGAAGGAGCTAACCGCTTTTTTGCACAACATGGGGGATCATGTAACTCGCCTTGATCGTTGGGAACCGGAGCTGAATGAAGCCATACCAAACGACGAGC
GTGACACCACGATGCCTGTAGCAATGGCAACAACCTTGCGTAAACTATTAACTGGCGAACTACTTACTCTAGCTTCCCGGCAACAGTTGATAGACTGGATG
GAGGCGGATAAAGTTGCAGGACCACTTCTGCGCTCGGCCCTTCCGGCTGGCTGGTTTATTGCTGATAAATCTGGAGCCGGTGAGCGTGGGTCTCGCGGTA
TCATTGCAGCACTGGGGCCAGATGGTAAGCCCTCCCGTATCGTAGTTATCTACACGACGGGGAGTCAGGCAACTATGGATGAACGAAATAGACAGATCG
CTGAGATAGGTGCCTCACTGATTAAGCATTGGTAACCGATTCTAGGTGCATTGGCGCAGAAAAAAATGCCTGATGCGACGCTGCGCGTCTTATACTCCCAC
ATATGCCAGATTCAGCAACGGATACGGCTTCCCCAACTTGCCCACTTCCATACGTGTCCTCCTTACCAGAAATTTATCCTTAAGATCGTTTAAACTCGACTCT
GGCTCTATCGAATCTCCGTCGTTTCGAGCTTACGCGAACAGCCGTGGCGCTCATTTGCTCGTCGGGCATCGAATCTCGTCAGCTATCGTCAGCTTACCTTTT
TGGCAGCGATCGCGGCTCCCGACATCTTGGACCATTAGCTCCACAGGTATCTTCTTCCCTCTAGTGGTCATAACAGCAGCTTCAGCTACCTCTCAATTCAAA
AAACCCCTCAAGACCCGTTTAGAGGCCCCAAGGGGTTATGCTATCAATCGTTGCGTTACACACACAAAAAACCAACACACATCCATCTTCGATGGATAGCG
ATTTTATTATCTAACTGCTGATCGAGTGTAGCCAGATCTAGTAATCAATTACGGGGTCATTAGTTCATAGCCCATATATGGAGTTCCGCGTTACATAACTTA

CGGTAAATGGCCCGCCTGGCTGACCGCCCAACGACCCCCGCCCATTGACGTCAATAATGACGTATGTTCCCATAGTAACGCCAATAGGGACTTTCCATTGA
CGTCAATGGGTGGAGTATTTACGGTAAACTGCCCACTTGGCAGTACATCAAGTGTATCATATGCCAAGTACGCCCCCTATTGACGTCAATGACGGTAAATG
GCCCGCCTGGCATTATGCCCAGTACATGACCTTATGGGACTTTCCTACTTGGCAGTACATCTACGTATTAGTCATCGCTATTACCATGCTGATGCGGTTTTG
GCAGTACATCAATGGGCGTGGATAGCGGTTTGACTCACGGGGATTTCCAAGTCTCCACCCCATTGACGTCAATGGGAGTTTGTTTTGGCACCAAAATCAAC
GGGACTTTCCAAAATGTCGTAACAACTCCGCCCCATTGACGCAAATGGGCGGTAGGCGTGTACGGTGGGAGGTCTATATAAGCAGAGCTGGTTTAGTGA
ACCGTCAGATCAGATCTTTGTCGATCCTACCATCCACTCGACACACCCGCCAGCGGCCGCATGTACCCGTACGATGTCCCCGACTACGCCGGATCAGGCTC
T

P21757

GAGCAGTGGGACCACTTCCATAATCAACAGGAGGACACAGACAGTTGCTCTGAAAGCGTGAAATTTGATGCGCGATCAATGACTGCCCTTCTTCCTCCTAA
CCCGAAAAATTCTCCCAGCTTGCAAGAGAAGCTGAAGTCTTTCAAAGCCGCTCTGATCGCGCTGTACCTGTTGGTGTTTGCCGTTCTTATACCACTGATAGG
CATTGTAGCAGCACAACTGCTTAAGTGGGAAACCAAAAACTGTTCAGTAAGTAGCACTAACGCGAATGACATAACCCAGAGCCTGACAGGCAAAGGAAA
TGATTCTGAAGAAGAGATGCGCTTTCAGGAAGTCTTCATGGAACACATGTCTAATATGGAGAAACGAATTCAGCATATCCTGGACATGGAGGCGAATCTG
ATGGACACTGAACATTTTCAGAACTTTTCCATGACCACCGATCAGAGATTCAACGACATACTGCTCCAGCTGAGCACACTCTTCTCCAGCGTCCAAGGGCAT
GGGAACGCCATCGACGAAATAAGCAAAAGCCTCATATCACTGAATACAACCTTGCTGGATCTCCAGCTCAATATCGAAAATCTGAATGGTAAAATCCAGG
AAAACACCTTCAAGCAGCAGGAGGAAATTAGCAAGTTGGAGGAACGCGTGTATAACGTCAGCGCCGAGATCATGGCAATGAAGGAAGAGCAAGTTCAC
CTTGAACAAGAGATCAAAGGCGAAGTGAAGGTCCTGAATAATATCACAAACGACTTAAGGTTGAAGGACTGGGAGCATAGTCAAACTCTTAGAAATATC
ACTTTAATCCAAGGGCCACCCGGTCCGCCTGGCGAGAAAGGCGATCGTGGACCTACAGGTGAATCAGGCCCAAGGGGATTCCCTGGTCCAATTGGCCCTC
CTGGGCTTAAGGGTGATAGGGGAGCTATAGGCTTCCCCGGATCCCGCGGCCTGCCTGGATACGCCGGCCGTCCTGGAAACTCCGGCCCTAAGGGGCAGA
AGGGGGAGAAGGGATCCGGTAACACATTAACTCCCTTTACCAAGGTTCGGTTGGTTGGAGGCAGCGGCCCTCATGAAGGAAGGGTGGAGATCCTGCACA
GCGGCCAGTGGGGCACCATTTGCGATGACCGGTGGGAAGTCCGCGTGGGACAGGTGGTCTGCCGGAGTCTCGGTTATCCTGGCGTGCAGGCAGTGCAC
AAGGCCGCTCACTTCGGGCAGGGCACTGGCCCCATCTGGCTGAATGAAGTCTTCTGCTTCGGAAGGGAGAGCAGTATTGAGGAGTGCAAGATACGGCAG
TGGGGAACTAGGGCATGTTCACATTCCGAGGATGCAGGAGTGACGTGCACTCTGGGCAGTGGGTCCGATTACAAAGACCATGATGGCGATTATAAGGAC
CACGACATCGATTATAAAGATGACGATGATAAATAGGTCGAGCCGCGGCAATTCCGACGTTACTGGCCGAAGCCGCTTGGAATAAGGCCGGTGTGCGTTT
GTCTATATGTTATTTTCCACCATATTGCCGTCTTTTGGCAATGTGAGGGCCCGGAAACCTGGCCCTGTCTTCTTGACGAGCATTCCTAGGGGTCTTTCCCCTC
TCGCCAAAGGAATGCAAGGTCTGTTGAATGTCGTGAAGGAAGCAGTTCCTCTGGAAGCTTCTTGAAGACAAACAACGTCTGTAGCGACCCTTTGCAGGCA
GCGGAACCCCCCACCTGGCAACAGGTGCCTCTGCGGCCAAAAGCCACGTGTATAAGATACACCTGCAAAGGCGGCACAACCCCAGTGCCACGTTGTGAG
TTGGATAGTTGTGGAAAGAGTCAAATGGCTCTCCTCAAGCGTATTCAACAAGGGGCTGAAGGATGCCCAGAAGGTACCCCATTGTATGGGATCTGATCTG
GGGCCTCGGTGCACATGCTTTACATGTGTTTAGTCGAGGTTAAAAAAACGTCTAGGCCCCCCGAACCACGGGGACGTGGTTTTCCTTTGAAAAACACGAT
GATAATATGGTGTCAGTGATTAAGCCCGAGATGAAAATGAGATATTATATGGACGGGAGCGTCAATGGGCATGAGTTCACTATCGAAGGCGAGGGAACG
GGACGCCCTTATGAGGGGCACCAAGAAATGACACTCAGAGTAACTATGGCAGAAGGCGGTCCAATGCCATTTGCATTCGATCTGGTTAGCCACGTATTTT
GCTACGGGCATAGAGTTTTTACAAAATACCCCGAAGAGATCCCAGATTACTTTAAGCAGGCATTCCCTGAAGGCCTGTCCTGGGAACGATCCCTGGAGTTC
GAGGACGGGGGCAGCGCGAGTGTCTCCGCTCACATTAGTCTGAGGGGGAACACCTTTTATCATAAATCTAAATTCACAGGAGTCAATTTCCCAGCAGATG
GCCCAATTATGCAGAATCAGTCAGTTGATTGGGAGCCTTCTACGGAAAAGATCACTGCAAGCGACGGCGTGCTTAAGGGGGATGTCACCATGTATCTGAA
ACTGGAAGGGGGCGGAAACCATAAGTGTCAAATGAAAACTACCTATAAGGCCGCAAAGGAGATTCTCGAGATGCCCGGCGATCATTACATCGGTCATCG
ATTAGTTCGTAAGACCGAGGGAAATATCACTGAACAGGTCGAGGACGCTGTTGCACACTCATGAGCTAGCTTGACTGACTGAGATACAGCGTACCTTCAG
CTCACAGACATGATAAGATACATTGATGAGTTTGGACAAACCACAACTAGAATGCAGTGAAAAAAATGCTTTATTTGTGAAATTTGTGATGCTATTGCTTT
ATTTGTAACCATTATAAGCTGCAATAAACAAGTTAACAACAACAATTGCATTCATTTTATGTTTCAGGTTCAGGGGGAGGTGTGGGAGGTTTTTTAAAGCA
AGTAAAACCTCTACAAATGTGGTATTGGCCCATCTCTATCGGTATCGTAGCATAACCCCTTGGGGCCTCTAAACGGGTCTTGAGGGGTTTTTTGTGCCCCTC
GGGCCGGATTGCTATCTACCGGCATTGGCGCAGAAAAAAATGCCTGATGCGACGCTGCGCGTCTTATACTCCCACATATGCCAGATTCAGCAACGGATAC
GGCTTCCCCAACTTGCCCACTTCCATACGTGTCCTCCTTACCAGAAATTTATCCTTAAGGTCGTCAGCTATCCTGCAGGCGATCTCTCGATTTCGATCAAGAC
ATTCCTTTAATGGTCTTTTCTGGACACCACTAGGGGTCAGAAGTAGTTCATCAAACTTTCTTCCCTCCCTAATCTCATTGGTTACCTTGGGCTATCGAAACTT
AATTAACCAGTCAAGTCAGCTACTTGGCGAGATCGACTTGTCTGGGTTTCGACTACGCTCAGAATTGCGTCAGTCAAGTTCGATCTGGTCCTTGCTATTGCA
CCCGTTCTCCGATTACGAGTTTCATTTAAATCATGTGAGCAAAAGGCCAGCAAAAGGCCAGGAACCGTAAAAAGGCCGCGTTGCTGGCGTTTTTCCATAG
GCTCCGCCCCCCTGACGAGCATCACAAAAATCGACGCTCAAGTCAGAGGTGGCGAAACCCGACAGGACTATAAAGATACCAGGCGTTTCCCCCTGGAAGC
TCCCTCGTGCGCTCTCCTGTTCCGACCCTGCCGCTTACCGGATACCTGTCCGCCTTTCTCCCTTCGGGAAGCGTGGCGCTTTCTCATAGCTCACGCTGTAGGT
ATCTCAGTTCGGTGTAGGTCGTTCGCTCCAAGCTGGGCTGTGTGCACGAACCCCCCGTTCAGCCCGACCGCTGCGCCTTATCCGGTAACTATCGTCTTGAG
TCCAACCCGGTAAGACACGACTTATCGCCACTGGCAGCAGCCACTGGTAACAGGATTAGCAGAGCGAGGTATGTAGGCGGTGCTACAGAGTTCTTGAAG
TGGTGGCCTAACTACGGCTACACTAGAAGAACAGTATTTGGTATCTGCGCTCTGCTGAAGCCAGTTACCTTCGGAAAAAGAGTTGGTAGCTCTTGATCCG
GCAAACAAACCACCGCTGGTAGCGGTGGTTTTTTTGTTTGCAAGCAGCAGATTACGCGCAGAAAAAAAGGATCTCAAGAAGATCCTTTGATCTTTTCTACG
GGGTCTGACGCTCAGTGGAACGAAAACTCACGTTAAGGGATTTTGGTCATGAGATTATCAAAAAGGATCTTCACCTAGATCCTTTTAAATTAAAAATGAAG
TTTTAAATCAATCTAAAGTATATATGAGTAAACTTGGTCTGACAGTTACCAATGCTTAATCAGTGAGGCACCTATCTCAGCGATCTGTCTATTTCGTTCATCC
ATAGTTGCATTTAAATTTCCGAACTCTCCAAGGCCCTCGTCGGAAAATCTTCAAACCTTTCGTCCGATCCATCTTGCAGGCTACCTCTCGAACGAACTATCGC
AAGTCTCTTGGCCGGCCTTGCGCCTTGGCTATTGCTTGGCAGCGCCTATCGCCAGGTATTACTCCAATCCCGAATATCCGAGATCGGGATCACCCGAGAGA
AGTTCAACCTACATCCTCAATCCCGATCTATCCGAGATCCGAGGAATATCGAAATCGGGGCGCGCCTGGCCTCCGCGCCGGGTTTTGGCGCCTCCCGCGG
GCGCCCCCCTCGTCACGGCGAGCGCTGCCACGTCAGACGAAGGGCGCAGGAGCGTCCTGATCCTTCCGCCCGGACGCTCAGGACAGCGGCCCGCTGCTC
ATAAGACTCGGCCTTAGAACCCCAGTATCAGCAGAAGGACATTTTAGGACGGGACTTGGGTGACTCTAGGGCACTGGTTTTCTTTCCAGAGAGCGGAACA
GGCGAGGAAAAGTAGTCCCTTCTCGGCGATTCTGCGGAGGGATCTCCGTGGGGCGGTGAACGCCGATGATTATATAAGGACGCGCCGGGTGTGGCACA
GCTAGTTCCGTCGCAGCCGGGATTTGGGTCGCGGTTCTTGTTTGTGGATCGCTGTGATCGTCACTTGGTGAGTAGCGGGCTGCTGGGCTGGCCGGGGCTT
TCGTGGCCGCCGGGCCGCTCGGTGGGACGGAAGCGTGTGGAGAGACCGCCAAGGGCTGTAGTCTGGGTCCGCGAGCAAGGTTGCCCTGAACTGGGGGT
TGGGGGGAGCGCAGCAAAATGGCGGCTGTTCCCGAGTCTTGAATGGAAGACGCTTGTGAGGCGGGCTGTGAGGTCGTTGAAACAAGGTGGGGGGCAT
GGTGGGCGGCAAGAACCCAAGGTCTTGAGCCCTTCGCTAATGCGGGAAAGCTCTTATTCGGGTGAGATGGGCTGGGCACCATCTGGGGACCCTGACGTG
AAGTTTGTCACTGACTGGAGAACTCGGTTTGTCGTCTGTTGCGGGGGCGGCAGTTATGGCGGTGCCGTTGGGCAGTGCACCCGTACCTTTGGGAGCGCG
CGCCCTCGTCGTGTCGTGACGTCACCCGTTCTGTTGGCTTATAATGCAGGGTGGGGCCACCTGCCGGTAGGTGTGCGGTAGGCTTTTCTCCGTCGCAGGA
CGCAGGGTTCGGGCCTAGGGTAGGCTCTCCTGAATCGACAGGCGCCGGACCTCTGGTGAGGGGAGGGATAAGTGAGGCGTCAGTTTCTTTGGTCGGTTT
TATGTACCTATCTTCTTAAGTAGCTGAAGCTCCGGTTTTGAACTATGCGCTCGGGGTTGGCGAGTGTGTTTTGTGAAGTTTTTTAGGCACCTTTTGAAATGT
AATCATTTGGGTCAATATGTAATTTTCAGTGTTAGACTTGTAAATTGTCCGCTAAATTCTGGCCGTTTTTGGCTTTTTTGTTAGACAACATGACCGAGTACAA
GCCCACGGTGCGCCTCGCCACCCGCGACGACGTCCCCAGGGCCGTACGCACCCTCGCCGCCGCGTTCGCCGACTACCCCGCCACGCGCCACACCGTCGAT

CCGGACCGCCACATCGAGCGGGTCACCGAGCTGCAAGAACTCTTCCTCACGCGCGTCGGGCTCGACATCGGCAAGGTGTGGGTCGCGGACGACGGCGCC
GCGGTGGCGGTCTGGACCACGCCGGAGAGCGTCGAAGCGGGGGCGGTGTTCGCCGAGATCGGCCCGCGCATGGCCGAGTTGAGCGGTTCCCGGCTGG
CCGCGCAGCAACAGATGGAGGGCCTCCTGGCGCCGCACCGGCCCAAGGAGCCCGCGTGGTTCCTGGCCACCGTCGGCGTCTCGCCCGACCACCAGGGCA
AGGGTCTGGGCAGCGCCGTCGTGCTCCCCGGAGTGGAGGCCGCCGAGCGCGCCGGGGTGCCCGCCTTCCTGGAGACCTCCGCGCCCCGCAACCTCCCCT
TCTACGAGCGGCTCGGCTTCACCGTCACCGCCGACGTCGAGGTGCCCGAAGGACCGCGCACCTGGTGCATGACCCGCAAGCCCGGTGCCTAGGCTAGTA
TGTAAGCCTAGTCTTAGATAATAAAATCGCTATCCATCGAAGATGGATGTGTGTTGGTTTTTTGTGTGTGTAACGCTAGGCGCGCCTGGTGTACCGAGAAC
GATCCTCTCAGTGCGAGTCTCGACGATCCATATCGTTGCTTGGCAGTCAGCCAGTCGGAATCCAGCTTGGGACCCAGGAAGTCCAATCGTCAGATATTGTA
CTCAAGCCTGGTCACGGCAGCGTACCGATCTGTTTAAACCTAGATATTGATAGTCTGATCGGTCAACGTATAATCGAGTCCTAGCTTTTGCAAACATCTATC
AAGAGACAGGATCAGCAGGAGGCTTTCGCATGAGTATTCAACATTTCCGTGTCGCCCTTATTCCCTTTTTTGCGGCATTTTGCCTTCCTGTTTTTGCTCACCC
AGAAACGCTGGTGAAAGTAAAAGATGCTGAAGATCAGTTGGGTGCGCGAGTGGGTTACATCGAACTGGATCTCAACAGCGGTAAGATCCTTGAGAGTTT
TCGCCCCGAAGAACGCTTTCCAATGATGAGCACTTTTAAAGTTCTGCTATGTGGCGCGGTATTATCCCGTATTGACGCCGGGCAAGAGCAACTCGGTCGCC
GCATACACTATTCTCAGAATGACTTGGTTGAGTATTCACCAGTCACAGAAAAGCATCTTACGGATGGCATGACAGTAAGAGAATTATGCAGTGCTGCCATA
ACCATGAGTGATAACACTGCGGCCAACTTACTTCTGACAACGATTGGAGGACCGAAGGAGCTAACCGCTTTTTTGCACAACATGGGGGATCATGTAACTC
GCCTTGATCGTTGGGAACCGGAGCTGAATGAAGCCATACCAAACGACGAGCGTGACACCACGATGCCTGTAGCAATGGCAACAACCTTGCGTAAACTATT
AACTGGCGAACTACTTACTCTAGCTTCCCGGCAACAGTTGATAGACTGGATGGAGGCGGATAAAGTTGCAGGACCACTTCTGCGCTCGGCCCTTCCGGCT
GGCTGGTTTATTGCTGATAAATCTGGAGCCGGTGAGCGTGGGTCTCGCGGTATCATTGCAGCACTGGGGCCAGATGGTAAGCCCTCCCGTATCGTAGTTA
TCTACACGACGGGGAGTCAGGCAACTATGGATGAACGAAATAGACAGATCGCTGAGATAGGTGCCTCACTGATTAAGCATTGGTAACCGATTCTAGGTG
CATTGGCGCAGAAAAAAATGCCTGATGCGACGCTGCGCGTCTTATACTCCCACATATGCCAGATTCAGCAACGGATACGGCTTCCCCAACTTGCCCACTTC
CATACGTGTCCTCCTTACCAGAAATTTATCCTTAAGATCGTTTAAACTCGACTCTGGCTCTATCGAATCTCCGTCGTTTCGAGCTTACGCGAACAGCCGTGG
CGCTCATTTGCTCGTCGGGCATCGAATCTCGTCAGCTATCGTCAGCTTACCTTTTTGGCAGCGATCGCGGCTCCCGACATCTTGGACCATTAGCTCCACAGG
TATCTTCTTCCCTCTAGTGGTCATAACAGCAGCTTCAGCTACCTCTCAATTCAAAAAACCCCTCAAGACCCGTTTAGAGGCCCCAAGGGGTTATGCTATCAA
TCGTTGCGTTACACACACAAAAAACCAACACACATCCATCTTCGATGGATAGCGATTTTATTATCTAACTGCTGATCGAGTGTAGCCAGATCTAGTAATCAA
TTACGGGGTCATTAGTTCATAGCCCATATATGGAGTTCCGCGTTACATAACTTACGGTAAATGGCCCGCCTGGCTGACCGCCCAACGACCCCCGCCCATTG
ACGTCAATAATGACGTATGTTCCCATAGTAACGCCAATAGGGACTTTCCATTGACGTCAATGGGTGGAGTATTTACGGTAAACTGCCCACTTGGCAGTACA
TCAAGTGTATCATATGCCAAGTACGCCCCCTATTGACGTCAATGACGGTAAATGGCCCGCCTGGCATTATGCCCAGTACATGACCTTATGGGACTTTCCTAC
TTGGCAGTACATCTACGTATTAGTCATCGCTATTACCATGCTGATGCGGTTTTGGCAGTACATCAATGGGCGTGGATAGCGGTTTGACTCACGGGGATTTC
CAAGTCTCCACCCCATTGACGTCAATGGGAGTTTGTTTTGGCACCAAAATCAACGGGACTTTCCAAAATGTCGTAACAACTCCGCCCCATTGACGCAAATG
GGCGGTAGGCGTGTACGGTGGGAGGTCTATATAAGCAGAGCTGGTTTAGTGAACCGTCAGATCAGATCTTTGTCGATCCTACCATCCACTCGACACACCC
GCCAGCGGCCGCATGTACCCGTACGATGTCCCCGACTACGCCGGATCAGGCTCT

P62995

TCCGACAGTGGTGAACAGAATTACGGCGAGCGCGAGTCAAGGTCAGCCAGCAGGTCCGGTTCCGCGCACGGCAGTGGTAAGAGTGCTAGACATACGCCT
GCACGAAGCAGGAGCAAGGAGGATAGCAGAAGATCCAGGTCTAAGAGCCGTTCAAGGAGCGAGTCTAGATCCCGCTCCCGCCGTAGTTCTAGACGCCAC
TACACTAGATCAAGATCAAGGTCACGCTCCCACAGACGATCTCGCAGCCGGAGTTATTCTAGGGACTACAGAAGGAGGCATTCCCATAGCCATAGCCCAA
TGAGTACGAGAAGAAGGCATGTGGGCAACAGAGCTAATCCCGACCCTAATTGCTGTCTGGGGGTGTTCGGCCTGAGTCTGTACACAACAGAGCGGGATC
TTCGCGAGGTATTCTCCAAGTATGGGCCCATCGCAGACGTGAGTATAGTTTATGACCAGCAATCACGACGCTCTCGAGGGTTTGCCTTTGTCTATTTTGAA
AATGTAGACGACGCTAAGGAAGCAAAAGAGCGAGCTAACGGAATGGAGCTCGATGGCAGGCGGATCAGAGTGGACTTCTCTATTACAAAAAGGCCACA
CACGCCCACACCAGGCATATACATGGGAAGGCCCACTTATGGCTCATCAAGGCGACGCGACTATTACGATCGGGGCTACGACAGGGGGTATGACGATCG
CGACTATTACTCACGCTCATATCGAGGGGGCGGAGGTGGCGGCGGAGGTTGGAGGGCCGCTCAGGACCGCGATCAGATTTACCGCCGGAGATCTCCAA
GTCCGTATTACTCTCGAGGCGGGTATCGTTCACGCTCTAGATCCAGATCCTATAGTCCTCGTCGGTATGGCAGTGGGTCCGATTACAAAGACCATGATGGC
GATTATAAGGACCACGACATCGATTATAAAGATGACGATGATAAATAGGTCGAGCCGCGGCAATTCCGACGTTACTGGCCGAAGCCGCTTGGAATAAGG
CCGGTGTGCGTTTGTCTATATGTTATTTTCCACCATATTGCCGTCTTTTGGCAATGTGAGGGCCCGGAAACCTGGCCCTGTCTTCTTGACGAGCATTCCTAG
GGGTCTTTCCCCTCTCGCCAAAGGAATGCAAGGTCTGTTGAATGTCGTGAAGGAAGCAGTTCCTCTGGAAGCTTCTTGAAGACAAACAACGTCTGTAGCG
ACCCTTTGCAGGCAGCGGAACCCCCCACCTGGCAACAGGTGCCTCTGCGGCCAAAAGCCACGTGTATAAGATACACCTGCAAAGGCGGCACAACCCCAGT
GCCACGTTGTGAGTTGGATAGTTGTGGAAAGAGTCAAATGGCTCTCCTCAAGCGTATTCAACAAGGGGCTGAAGGATGCCCAGAAGGTACCCCATTGTAT
GGGATCTGATCTGGGGCCTCGGTGCACATGCTTTACATGTGTTTAGTCGAGGTTAAAAAAACGTCTAGGCCCCCCGAACCACGGGGACGTGGTTTTCCTTT
GAAAAACACGATGATAATATGGTGTCAGTGATTAAGCCCGAGATGAAAATGAGATATTATATGGACGGGAGCGTCAATGGGCATGAGTTCACTATCGAA
GGCGAGGGAACGGGACGCCCTTATGAGGGGCACCAAGAAATGACACTCAGAGTAACTATGGCAGAAGGCGGTCCAATGCCATTTGCATTCGATCTGGTT
AGCCACGTATTTTGCTACGGGCATAGAGTTTTTACAAAATACCCCGAAGAGATCCCAGATTACTTTAAGCAGGCATTCCCTGAAGGCCTGTCCTGGGAACG
ATCCCTGGAGTTCGAGGACGGGGGCAGCGCGAGTGTCTCCGCTCACATTAGTCTGAGGGGGAACACCTTTTATCATAAATCTAAATTCACAGGAGTCAAT
TTCCCAGCAGATGGCCCAATTATGCAGAATCAGTCAGTTGATTGGGAGCCTTCTACGGAAAAGATCACTGCAAGCGACGGCGTGCTTAAGGGGGATGTCA
CCATGTATCTGAAACTGGAAGGGGGCGGAAACCATAAGTGTCAAATGAAAACTACCTATAAGGCCGCAAAGGAGATTCTCGAGATGCCCGGCGATCATT
ACATCGGTCATCGATTAGTTCGTAAGACCGAGGGAAATATCACTGAACAGGTCGAGGACGCTGTTGCACACTCATGAGCTAGCTTGACTGACTGAGATAC
AGCGTACCTTCAGCTCACAGACATGATAAGATACATTGATGAGTTTGGACAAACCACAACTAGAATGCAGTGAAAAAAATGCTTTATTTGTGAAATTTGTG
ATGCTATTGCTTTATTTGTAACCATTATAAGCTGCAATAAACAAGTTAACAACAACAATTGCATTCATTTTATGTTTCAGGTTCAGGGGGAGGTGTGGGAG
GTTTTTTAAAGCAAGTAAAACCTCTACAAATGTGGTATTGGCCCATCTCTATCGGTATCGTAGCATAACCCCTTGGGGCCTCTAAACGGGTCTTGAGGGGT
TTTTTGTGCCCCTCGGGCCGGATTGCTATCTACCGGCATTGGCGCAGAAAAAAATGCCTGATGCGACGCTGCGCGTCTTATACTCCCACATATGCCAGATT
CAGCAACGGATACGGCTTCCCCAACTTGCCCACTTCCATACGTGTCCTCCTTACCAGAAATTTATCCTTAAGGTCGTCAGCTATCCTGCAGGCGATCTCTCG
ATTTCGATCAAGACATTCCTTTAATGGTCTTTTCTGGACACCACTAGGGGTCAGAAGTAGTTCATCAAACTTTCTTCCCTCCCTAATCTCATTGGTTACCTTG
GGCTATCGAAACTTAATTAACCAGTCAAGTCAGCTACTTGGCGAGATCGACTTGTCTGGGTTTCGACTACGCTCAGAATTGCGTCAGTCAAGTTCGATCTG
GTCCTTGCTATTGCACCCGTTCTCCGATTACGAGTTTCATTTAAATCATGTGAGCAAAAGGCCAGCAAAAGGCCAGGAACCGTAAAAAGGCCGCGTTGCTG
GCGTTTTTCCATAGGCTCCGCCCCCCTGACGAGCATCACAAAAATCGACGCTCAAGTCAGAGGTGGCGAAACCCGACAGGACTATAAAGATACCAGGCGT
TTCCCCCTGGAAGCTCCCTCGTGCGCTCTCCTGTTCCGACCCTGCCGCTTACCGGATACCTGTCCGCCTTTCTCCCTTCGGGAAGCGTGGCGCTTTCTCATAG
CTCACGCTGTAGGTATCTCAGTTCGGTGTAGGTCGTTCGCTCCAAGCTGGGCTGTGTGCACGAACCCCCCGTTCAGCCCGACCGCTGCGCCTTATCCGGTA
ACTATCGTCTTGAGTCCAACCCGGTAAGACACGACTTATCGCCACTGGCAGCAGCCACTGGTAACAGGATTAGCAGAGCGAGGTATGTAGGCGGTGCTAC
AGAGTTCTTGAAGTGGTGGCCTAACTACGGCTACACTAGAAGAACAGTATTTGGTATCTGCGCTCTGCTGAAGCCAGTTACCTTCGGAAAAAGAGTTGGT
AGCTCTTGATCCGGCAAACAAACCACCGCTGGTAGCGGTGGTTTTTTTGTTTGCAAGCAGCAGATTACGCGCAGAAAAAAAGGATCTCAAGAAGATCCTT
TGATCTTTTCTACGGGGTCTGACGCTCAGTGGAACGAAAACTCACGTTAAGGGATTTTGGTCATGAGATTATCAAAAAGGATCTTCACCTAGATCCTTTTA

AATTAAAAATGAAGTTTTAAATCAATCTAAAGTATATATGAGTAAACTTGGTCTGACAGTTACCAATGCTTAATCAGTGAGGCACCTATCTCAGCGATCTGT
CTATTTCGTTCATCCATAGTTGCATTTAAATTTCCGAACTCTCCAAGGCCCTCGTCGGAAAATCTTCAAACCTTTCGTCCGATCCATCTTGCAGGCTACCTCTC
GAACGAACTATCGCAAGTCTCTTGGCCGGCCTTGCGCCTTGGCTATTGCTTGGCAGCGCCTATCGCCAGGTATTACTCCAATCCCGAATATCCGAGATCGG
GATCACCCGAGAGAAGTTCAACCTACATCCTCAATCCCGATCTATCCGAGATCCGAGGAATATCGAAATCGGGGCGCGCCTGGCCTCCGCGCCGGGTTTT
GGCGCCTCCCGCGGGCGCCCCCCTCGTCACGGCGAGCGCTGCCACGTCAGACGAAGGGCGCAGGAGCGTCCTGATCCTTCCGCCCGGACGCTCAGGACA
GCGGCCCGCTGCTCATAAGACTCGGCCTTAGAACCCCAGTATCAGCAGAAGGACATTTTAGGACGGGACTTGGGTGACTCTAGGGCACTGGTTTTCTTTC
CAGAGAGCGGAACAGGCGAGGAAAAGTAGTCCCTTCTCGGCGATTCTGCGGAGGGATCTCCGTGGGGCGGTGAACGCCGATGATTATATAAGGACGCG
CCGGGTGTGGCACAGCTAGTTCCGTCGCAGCCGGGATTTGGGTCGCGGTTCTTGTTTGTGGATCGCTGTGATCGTCACTTGGTGAGTAGCGGGCTGCTGG
GCTGGCCGGGGCTTTCGTGGCCGCCGGGCCGCTCGGTGGGACGGAAGCGTGTGGAGAGACCGCCAAGGGCTGTAGTCTGGGTCCGCGAGCAAGGTTGC
CCTGAACTGGGGGTTGGGGGGAGCGCAGCAAAATGGCGGCTGTTCCCGAGTCTTGAATGGAAGACGCTTGTGAGGCGGGCTGTGAGGTCGTTGAAACA
AGGTGGGGGGCATGGTGGGCGGCAAGAACCCAAGGTCTTGAGCCCTTCGCTAATGCGGGAAAGCTCTTATTCGGGTGAGATGGGCTGGGCACCATCTG
GGGACCCTGACGTGAAGTTTGTCACTGACTGGAGAACTCGGTTTGTCGTCTGTTGCGGGGGCGGCAGTTATGGCGGTGCCGTTGGGCAGTGCACCCGTA
CCTTTGGGAGCGCGCGCCCTCGTCGTGTCGTGACGTCACCCGTTCTGTTGGCTTATAATGCAGGGTGGGGCCACCTGCCGGTAGGTGTGCGGTAGGCTTT
TCTCCGTCGCAGGACGCAGGGTTCGGGCCTAGGGTAGGCTCTCCTGAATCGACAGGCGCCGGACCTCTGGTGAGGGGAGGGATAAGTGAGGCGTCAGT
TTCTTTGGTCGGTTTTATGTACCTATCTTCTTAAGTAGCTGAAGCTCCGGTTTTGAACTATGCGCTCGGGGTTGGCGAGTGTGTTTTGTGAAGTTTTTTAGG
CACCTTTTGAAATGTAATCATTTGGGTCAATATGTAATTTTCAGTGTTAGACTTGTAAATTGTCCGCTAAATTCTGGCCGTTTTTGGCTTTTTTGTTAGACAA
CATGACCGAGTACAAGCCCACGGTGCGCCTCGCCACCCGCGACGACGTCCCCAGGGCCGTACGCACCCTCGCCGCCGCGTTCGCCGACTACCCCGCCACG
CGCCACACCGTCGATCCGGACCGCCACATCGAGCGGGTCACCGAGCTGCAAGAACTCTTCCTCACGCGCGTCGGGCTCGACATCGGCAAGGTGTGGGTC
GCGGACGACGGCGCCGCGGTGGCGGTCTGGACCACGCCGGAGAGCGTCGAAGCGGGGGCGGTGTTCGCCGAGATCGGCCCGCGCATGGCCGAGTTGA
GCGGTTCCCGGCTGGCCGCGCAGCAACAGATGGAGGGCCTCCTGGCGCCGCACCGGCCCAAGGAGCCCGCGTGGTTCCTGGCCACCGTCGGCGTCTCGC
CCGACCACCAGGGCAAGGGTCTGGGCAGCGCCGTCGTGCTCCCCGGAGTGGAGGCCGCCGAGCGCGCCGGGGTGCCCGCCTTCCTGGAGACCTCCGCG
CCCCGCAACCTCCCCTTCTACGAGCGGCTCGGCTTCACCGTCACCGCCGACGTCGAGGTGCCCGAAGGACCGCGCACCTGGTGCATGACCCGCAAGCCCG
GTGCCTAGGCTAGTATGTAAGCCTAGTCTTAGATAATAAAATCGCTATCCATCGAAGATGGATGTGTGTTGGTTTTTTGTGTGTGTAACGCTAGGCGCGCC
TGGTGTACCGAGAACGATCCTCTCAGTGCGAGTCTCGACGATCCATATCGTTGCTTGGCAGTCAGCCAGTCGGAATCCAGCTTGGGACCCAGGAAGTCCA
ATCGTCAGATATTGTACTCAAGCCTGGTCACGGCAGCGTACCGATCTGTTTAAACCTAGATATTGATAGTCTGATCGGTCAACGTATAATCGAGTCCTAGC
TTTTGCAAACATCTATCAAGAGACAGGATCAGCAGGAGGCTTTCGCATGAGTATTCAACATTTCCGTGTCGCCCTTATTCCCTTTTTTGCGGCATTTTGCCTT
CCTGTTTTTGCTCACCCAGAAACGCTGGTGAAAGTAAAAGATGCTGAAGATCAGTTGGGTGCGCGAGTGGGTTACATCGAACTGGATCTCAACAGCGGTA
AGATCCTTGAGAGTTTTCGCCCCGAAGAACGCTTTCCAATGATGAGCACTTTTAAAGTTCTGCTATGTGGCGCGGTATTATCCCGTATTGACGCCGGGCAA
GAGCAACTCGGTCGCCGCATACACTATTCTCAGAATGACTTGGTTGAGTATTCACCAGTCACAGAAAAGCATCTTACGGATGGCATGACAGTAAGAGAAT
TATGCAGTGCTGCCATAACCATGAGTGATAACACTGCGGCCAACTTACTTCTGACAACGATTGGAGGACCGAAGGAGCTAACCGCTTTTTTGCACAACATG
GGGGATCATGTAACTCGCCTTGATCGTTGGGAACCGGAGCTGAATGAAGCCATACCAAACGACGAGCGTGACACCACGATGCCTGTAGCAATGGCAACA
ACCTTGCGTAAACTATTAACTGGCGAACTACTTACTCTAGCTTCCCGGCAACAGTTGATAGACTGGATGGAGGCGGATAAAGTTGCAGGACCACTTCTGCG
CTCGGCCCTTCCGGCTGGCTGGTTTATTGCTGATAAATCTGGAGCCGGTGAGCGTGGGTCTCGCGGTATCATTGCAGCACTGGGGCCAGATGGTAAGCCC
TCCCGTATCGTAGTTATCTACACGACGGGGAGTCAGGCAACTATGGATGAACGAAATAGACAGATCGCTGAGATAGGTGCCTCACTGATTAAGCATTGGT
AACCGATTCTAGGTGCATTGGCGCAGAAAAAAATGCCTGATGCGACGCTGCGCGTCTTATACTCCCACATATGCCAGATTCAGCAACGGATACGGCTTCCC
CAACTTGCCCACTTCCATACGTGTCCTCCTTACCAGAAATTTATCCTTAAGATCGTTTAAACTCGACTCTGGCTCTATCGAATCTCCGTCGTTTCGAGCTTAC
GCGAACAGCCGTGGCGCTCATTTGCTCGTCGGGCATCGAATCTCGTCAGCTATCGTCAGCTTACCTTTTTGGCAGCGATCGCGGCTCCCGACATCTTGGAC
CATTAGCTCCACAGGTATCTTCTTCCCTCTAGTGGTCATAACAGCAGCTTCAGCTACCTCTCAATTCAAAAAACCCCTCAAGACCCGTTTAGAGGCCCCAAG
GGGTTATGCTATCAATCGTTGCGTTACACACACAAAAAACCAACACACATCCATCTTCGATGGATAGCGATTTTATTATCTAACTGCTGATCGAGTGTAGCC
AGATCTAGTAATCAATTACGGGGTCATTAGTTCATAGCCCATATATGGAGTTCCGCGTTACATAACTTACGGTAAATGGCCCGCCTGGCTGACCGCCCAAC
GACCCCCGCCCATTGACGTCAATAATGACGTATGTTCCCATAGTAACGCCAATAGGGACTTTCCATTGACGTCAATGGGTGGAGTATTTACGGTAAACTGC
CCACTTGGCAGTACATCAAGTGTATCATATGCCAAGTACGCCCCCTATTGACGTCAATGACGGTAAATGGCCCGCCTGGCATTATGCCCAGTACATGACCT
TATGGGACTTTCCTACTTGGCAGTACATCTACGTATTAGTCATCGCTATTACCATGCTGATGCGGTTTTGGCAGTACATCAATGGGCGTGGATAGCGGTTT
GACTCACGGGGATTTCCAAGTCTCCACCCCATTGACGTCAATGGGAGTTTGTTTTGGCACCAAAATCAACGGGACTTTCCAAAATGTCGTAACAACTCCGC
CCCATTGACGCAAATGGGCGGTAGGCGTGTACGGTGGGAGGTCTATATAAGCAGAGCTGGTTTAGTGAACCGTCAGATCAGATCTTTGTCGATCCTACCA
TCCACTCGACACACCCGCCAGCGGCCGCATGTACCCGTACGATGTCCCCGACTACGCCGGATCAGGCTCT

P10636-8_2_441

GCTGAGCCCAGACAGGAGTTTGAAGTAATGGAGGATCACGCAGGCACTTACGGCCTGGGGGATAGGAAAGATCAGGGAGGCTACACTATGCATCAAGA
TCAGGAGGGGGACACAGATGCAGGACTGAAGGAATCCCCCCTTCAGACTCCTACAGAGGATGGCTCCGAGGAGCCTGGGTCCGAGACTAGTGATGCCA
AGAGTACTCCAACCGCCGAAGACGTGACTGCACCCCTGGTTGATGAGGGAGCACCTGGCAAACAGGCAGCAGCACAGCCTCATACAGAAATCCCTGAAG
GAACTACCGCTGAGGAGGCTGGAATAGGGGATACCCCATCCCTTGAGGACGAGGCCGCCGGGCACGTGACGCAGGCTAGAATGGTGAGCAAATCTAAA
GACGGAACCGGTTCCGATGACAAGAAAGCTAAGGGCGCCGATGGAAAGACCAAAATCGCGACCCCTAGGGGAGCAGCCCCGCCCGGCCAAAAAGGCCA
AGCCAATGCCACTAGGATCCCAGCAAAGACTCCCCCTGCCCCAAAGACACCCCCCTCCTCTGGAGAGCCCCCTAAGTCAGGGGATCGGTCCGGATACTCCT
CACCCGGAAGTCCAGGGACACCAGGATCCAGGTCACGCACCCCCAGCTTGCCCACCCCACCCACAAGGGAACCAAAAAAGGTGGCAGTTGTGAGGACCC
CGCCGAAATCACCTTCCTCTGCCAAATCCCGCCTCCAAACAGCGCCCGTGCCCATGCCGGACCTGAAGAACGTCAAGTCTAAAATTGGGTCTACAGAGAAT
CTGAAGCACCAGCCAGGAGGTGGTAAAGTGCAAATTATAAACAAAAAACTTGATCTCTCTAACGTACAGTCAAAATGCGGGTCAAAAGATAACATTAAGC
ATGTGCCTGGCGGCGGCAGTGTACAAATTGTTTATAAGCCCGTGGATCTGTCCAAAGTGACCAGTAAGTGTGGAAGTCTGGGCAACATCCACCATAAACC
CGGCGGTGGGCAGGTGGAGGTGAAGTCTGAAAAACTGGATTTCAAGGACCGCGTCCAAAGCAAGATCGGGTCACTCGATAATATTACCCATGTTCCAGG
CGGAGGTAACAAAAAGATCGAGACCCATAAACTGACTTTCCGGGAGAACGCCAAGGCCAAGACAGACCACGGGGCAGAGATTGTATACAAGTCTCCAGT
GGTCTCTGGAGACACCAGTCCTAGGCATTTATCCAATGTGTCATCAACCGGGTCTATAGACATGGTCGACTCTCCTCAGCTCGCTACGCTGGCTGACGAGG
TGTCTGCTAGTCTCGCAAAGCAAGGTCTGGGCAGTGGGTCCGATTACAAAGACCATGATGGCGATTATAAGGACCACGACATCGATTATAAAGATGACGA
TGATAAATAGGTCGAGCCGCGGCAATTCCGACGTTACTGGCCGAAGCCGCTTGGAATAAGGCCGGTGTGCGTTTGTCTATATGTTATTTTCCACCATATTG
CCGTCTTTTGGCAATGTGAGGGCCCGGAAACCTGGCCCTGTCTTCTTGACGAGCATTCCTAGGGGTCTTTCCCCTCTCGCCAAAGGAATGCAAGGTCTGTT
GAATGTCGTGAAGGAAGCAGTTCCTCTGGAAGCTTCTTGAAGACAAACAACGTCTGTAGCGACCCTTTGCAGGCAGCGGAACCCCCCACCTGGCAACAGG
TGCCTCTGCGGCCAAAAGCCACGTGTATAAGATACACCTGCAAAGGCGGCACAACCCCAGTGCCACGTTGTGAGTTGGATAGTTGTGGAAAGAGTCAAAT
GGCTCTCCTCAAGCGTATTCAACAAGGGGCTGAAGGATGCCCAGAAGGTACCCCATTGTATGGGATCTGATCTGGGGCCTCGGTGCACATGCTTTACATG
TGTTTAGTCGAGGTTAAAAAAACGTCTAGGCCCCCCGAACCACGGGGACGTGGTTTTCCTTTGAAAAACACGATGATAATATGGTGTCAGTGATTAAGCC

CGAGATGAAAATGAGATATTATATGGACGGGAGCGTCAATGGGCATGAGTTCACTATCGAAGGCGAGGGAACGGGACGCCCTTATGAGGGGCACCAAG
AAATGACACTCAGAGTAACTATGGCAGAAGGCGGTCCAATGCCATTTGCATTCGATCTGGTTAGCCACGTATTTTGCTACGGGCATAGAGTTTTTACAAAA
TACCCCGAAGAGATCCCAGATTACTTTAAGCAGGCATTCCCTGAAGGCCTGTCCTGGGAACGATCCCTGGAGTTCGAGGACGGGGGCAGCGCGAGTGTC
TCCGCTCACATTAGTCTGAGGGGGAACACCTTTTATCATAAATCTAAATTCACAGGAGTCAATTTCCCAGCAGATGGCCCAATTATGCAGAATCAGTCAGTT
GATTGGGAGCCTTCTACGGAAAAGATCACTGCAAGCGACGGCGTGCTTAAGGGGGATGTCACCATGTATCTGAAACTGGAAGGGGGCGGAAACCATAA
GTGTCAAATGAAAACTACCTATAAGGCCGCAAAGGAGATTCTCGAGATGCCCGGCGATCATTACATCGGTCATCGATTAGTTCGTAAGACCGAGGGAAAT
ATCACTGAACAGGTCGAGGACGCTGTTGCACACTCATGAGCTAGCTTGACTGACTGAGATACAGCGTACCTTCAGCTCACAGACATGATAAGATACATTG
ATGAGTTTGGACAAACCACAACTAGAATGCAGTGAAAAAAATGCTTTATTTGTGAAATTTGTGATGCTATTGCTTTATTTGTAACCATTATAAGCTGCAATA
AACAAGTTAACAACAACAATTGCATTCATTTTATGTTTCAGGTTCAGGGGGAGGTGTGGGAGGTTTTTTAAAGCAAGTAAAACCTCTACAAATGTGGTATT
GGCCCATCTCTATCGGTATCGTAGCATAACCCCTTGGGGCCTCTAAACGGGTCTTGAGGGGTTTTTTGTGCCCCTCGGGCCGGATTGCTATCTACCGGCAT
TGGCGCAGAAAAAAATGCCTGATGCGACGCTGCGCGTCTTATACTCCCACATATGCCAGATTCAGCAACGGATACGGCTTCCCCAACTTGCCCACTTCCAT
ACGTGTCCTCCTTACCAGAAATTTATCCTTAAGGTCGTCAGCTATCCTGCAGGCGATCTCTCGATTTCGATCAAGACATTCCTTTAATGGTCTTTTCTGGACA
CCACTAGGGGTCAGAAGTAGTTCATCAAACTTTCTTCCCTCCCTAATCTCATTGGTTACCTTGGGCTATCGAAACTTAATTAACCAGTCAAGTCAGCTACTTG
GCGAGATCGACTTGTCTGGGTTTCGACTACGCTCAGAATTGCGTCAGTCAAGTTCGATCTGGTCCTTGCTATTGCACCCGTTCTCCGATTACGAGTTTCATT
TAAATCATGTGAGCAAAAGGCCAGCAAAAGGCCAGGAACCGTAAAAAGGCCGCGTTGCTGGCGTTTTTCCATAGGCTCCGCCCCCCTGACGAGCATCACA
AAAATCGACGCTCAAGTCAGAGGTGGCGAAACCCGACAGGACTATAAAGATACCAGGCGTTTCCCCCTGGAAGCTCCCTCGTGCGCTCTCCTGTTCCGAC
CCTGCCGCTTACCGGATACCTGTCCGCCTTTCTCCCTTCGGGAAGCGTGGCGCTTTCTCATAGCTCACGCTGTAGGTATCTCAGTTCGGTGTAGGTCGTTCG
CTCCAAGCTGGGCTGTGTGCACGAACCCCCCGTTCAGCCCGACCGCTGCGCCTTATCCGGTAACTATCGTCTTGAGTCCAACCCGGTAAGACACGACTTAT
CGCCACTGGCAGCAGCCACTGGTAACAGGATTAGCAGAGCGAGGTATGTAGGCGGTGCTACAGAGTTCTTGAAGTGGTGGCCTAACTACGGCTACACTA
GAAGAACAGTATTTGGTATCTGCGCTCTGCTGAAGCCAGTTACCTTCGGAAAAAGAGTTGGTAGCTCTTGATCCGGCAAACAAACCACCGCTGGTAGCGG
TGGTTTTTTTGTTTGCAAGCAGCAGATTACGCGCAGAAAAAAAGGATCTCAAGAAGATCCTTTGATCTTTTCTACGGGGTCTGACGCTCAGTGGAACGAAA
ACTCACGTTAAGGGATTTTGGTCATGAGATTATCAAAAAGGATCTTCACCTAGATCCTTTTAAATTAAAAATGAAGTTTTAAATCAATCTAAAGTATATATG
AGTAAACTTGGTCTGACAGTTACCAATGCTTAATCAGTGAGGCACCTATCTCAGCGATCTGTCTATTTCGTTCATCCATAGTTGCATTTAAATTTCCGAACTC
TCCAAGGCCCTCGTCGGAAAATCTTCAAACCTTTCGTCCGATCCATCTTGCAGGCTACCTCTCGAACGAACTATCGCAAGTCTCTTGGCCGGCCTTGCGCCT
TGGCTATTGCTTGGCAGCGCCTATCGCCAGGTATTACTCCAATCCCGAATATCCGAGATCGGGATCACCCGAGAGAAGTTCAACCTACATCCTCAATCCCG
ATCTATCCGAGATCCGAGGAATATCGAAATCGGGGCGCGCCTGGCCTCCGCGCCGGGTTTTGGCGCCTCCCGCGGGCGCCCCCCTCGTCACGGCGAGCG
CTGCCACGTCAGACGAAGGGCGCAGGAGCGTCCTGATCCTTCCGCCCGGACGCTCAGGACAGCGGCCCGCTGCTCATAAGACTCGGCCTTAGAACCCCA
GTATCAGCAGAAGGACATTTTAGGACGGGACTTGGGTGACTCTAGGGCACTGGTTTTCTTTCCAGAGAGCGGAACAGGCGAGGAAAAGTAGTCCCTTCT
CGGCGATTCTGCGGAGGGATCTCCGTGGGGCGGTGAACGCCGATGATTATATAAGGACGCGCCGGGTGTGGCACAGCTAGTTCCGTCGCAGCCGGGATT
TGGGTCGCGGTTCTTGTTTGTGGATCGCTGTGATCGTCACTTGGTGAGTAGCGGGCTGCTGGGCTGGCCGGGGCTTTCGTGGCCGCCGGGCCGCTCGGT
GGGACGGAAGCGTGTGGAGAGACCGCCAAGGGCTGTAGTCTGGGTCCGCGAGCAAGGTTGCCCTGAACTGGGGGTTGGGGGGAGCGCAGCAAAATGG
CGGCTGTTCCCGAGTCTTGAATGGAAGACGCTTGTGAGGCGGGCTGTGAGGTCGTTGAAACAAGGTGGGGGGCATGGTGGGCGGCAAGAACCCAAGGT
CTTGAGCCCTTCGCTAATGCGGGAAAGCTCTTATTCGGGTGAGATGGGCTGGGCACCATCTGGGGACCCTGACGTGAAGTTTGTCACTGACTGGAGAACT
CGGTTTGTCGTCTGTTGCGGGGGCGGCAGTTATGGCGGTGCCGTTGGGCAGTGCACCCGTACCTTTGGGAGCGCGCGCCCTCGTCGTGTCGTGACGTCAC
CCGTTCTGTTGGCTTATAATGCAGGGTGGGGCCACCTGCCGGTAGGTGTGCGGTAGGCTTTTCTCCGTCGCAGGACGCAGGGTTCGGGCCTAGGGTAGG
CTCTCCTGAATCGACAGGCGCCGGACCTCTGGTGAGGGGAGGGATAAGTGAGGCGTCAGTTTCTTTGGTCGGTTTTATGTACCTATCTTCTTAAGTAGCTG
AAGCTCCGGTTTTGAACTATGCGCTCGGGGTTGGCGAGTGTGTTTTGTGAAGTTTTTTAGGCACCTTTTGAAATGTAATCATTTGGGTCAATATGTAATTTT
CAGTGTTAGACTTGTAAATTGTCCGCTAAATTCTGGCCGTTTTTGGCTTTTTTGTTAGACAACATGACCGAGTACAAGCCCACGGTGCGCCTCGCCACCCGC
GACGACGTCCCCAGGGCCGTACGCACCCTCGCCGCCGCGTTCGCCGACTACCCCGCCACGCGCCACACCGTCGATCCGGACCGCCACATCGAGCGGGTCA
CCGAGCTGCAAGAACTCTTCCTCACGCGCGTCGGGCTCGACATCGGCAAGGTGTGGGTCGCGGACGACGGCGCCGCGGTGGCGGTCTGGACCACGCCG
GAGAGCGTCGAAGCGGGGGCGGTGTTCGCCGAGATCGGCCCGCGCATGGCCGAGTTGAGCGGTTCCCGGCTGGCCGCGCAGCAACAGATGGAGGGCC
TCCTGGCGCCGCACCGGCCCAAGGAGCCCGCGTGGTTCCTGGCCACCGTCGGCGTCTCGCCCGACCACCAGGGCAAGGGTCTGGGCAGCGCCGTCGTGC
TCCCCGGAGTGGAGGCCGCCGAGCGCGCCGGGGTGCCCGCCTTCCTGGAGACCTCCGCGCCCCGCAACCTCCCCTTCTACGAGCGGCTCGGCTTCACCGT
CACCGCCGACGTCGAGGTGCCCGAAGGACCGCGCACCTGGTGCATGACCCGCAAGCCCGGTGCCTAGGCTAGTATGTAAGCCTAGTCTTAGATAATAAA
ATCGCTATCCATCGAAGATGGATGTGTGTTGGTTTTTTGTGTGTGTAACGCTAGGCGCGCCTGGTGTACCGAGAACGATCCTCTCAGTGCGAGTCTCGACG
ATCCATATCGTTGCTTGGCAGTCAGCCAGTCGGAATCCAGCTTGGGACCCAGGAAGTCCAATCGTCAGATATTGTACTCAAGCCTGGTCACGGCAGCGTA
CCGATCTGTTTAAACCTAGATATTGATAGTCTGATCGGTCAACGTATAATCGAGTCCTAGCTTTTGCAAACATCTATCAAGAGACAGGATCAGCAGGAGGC
TTTCGCATGAGTATTCAACATTTCCGTGTCGCCCTTATTCCCTTTTTTGCGGCATTTTGCCTTCCTGTTTTTGCTCACCCAGAAACGCTGGTGAAAGTAAAAG
ATGCTGAAGATCAGTTGGGTGCGCGAGTGGGTTACATCGAACTGGATCTCAACAGCGGTAAGATCCTTGAGAGTTTTCGCCCCGAAGAACGCTTTCCAAT
GATGAGCACTTTTAAAGTTCTGCTATGTGGCGCGGTATTATCCCGTATTGACGCCGGGCAAGAGCAACTCGGTCGCCGCATACACTATTCTCAGAATGACT
TGGTTGAGTATTCACCAGTCACAGAAAAGCATCTTACGGATGGCATGACAGTAAGAGAATTATGCAGTGCTGCCATAACCATGAGTGATAACACTGCGGC
CAACTTACTTCTGACAACGATTGGAGGACCGAAGGAGCTAACCGCTTTTTTGCACAACATGGGGGATCATGTAACTCGCCTTGATCGTTGGGAACCGGAG
CTGAATGAAGCCATACCAAACGACGAGCGTGACACCACGATGCCTGTAGCAATGGCAACAACCTTGCGTAAACTATTAACTGGCGAACTACTTACTCTAG
CTTCCCGGCAACAGTTGATAGACTGGATGGAGGCGGATAAAGTTGCAGGACCACTTCTGCGCTCGGCCCTTCCGGCTGGCTGGTTTATTGCTGATAAATCT
GGAGCCGGTGAGCGTGGGTCTCGCGGTATCATTGCAGCACTGGGGCCAGATGGTAAGCCCTCCCGTATCGTAGTTATCTACACGACGGGGAGTCAGGCA
ACTATGGATGAACGAAATAGACAGATCGCTGAGATAGGTGCCTCACTGATTAAGCATTGGTAACCGATTCTAGGTGCATTGGCGCAGAAAAAAATGCCTG
ATGCGACGCTGCGCGTCTTATACTCCCACATATGCCAGATTCAGCAACGGATACGGCTTCCCCAACTTGCCCACTTCCATACGTGTCCTCCTTACCAGAAAT
TTATCCTTAAGATCGTTTAAACTCGACTCTGGCTCTATCGAATCTCCGTCGTTTCGAGCTTACGCGAACAGCCGTGGCGCTCATTTGCTCGTCGGGCATCGA
ATCTCGTCAGCTATCGTCAGCTTACCTTTTTGGCAGCGATCGCGGCTCCCGACATCTTGGACCATTAGCTCCACAGGTATCTTCTTCCCTCTAGTGGTCATAA
CAGCAGCTTCAGCTACCTCTCAATTCAAAAAACCCCTCAAGACCCGTTTAGAGGCCCCAAGGGGTTATGCTATCAATCGTTGCGTTACACACACAAAAAAC
CAACACACATCCATCTTCGATGGATAGCGATTTTATTATCTAACTGCTGATCGAGTGTAGCCAGATCTAGTAATCAATTACGGGGTCATTAGTTCATAGCCC
ATATATGGAGTTCCGCGTTACATAACTTACGGTAAATGGCCCGCCTGGCTGACCGCCCAACGACCCCCGCCCATTGACGTCAATAATGACGTATGTTCCCA
TAGTAACGCCAATAGGGACTTTCCATTGACGTCAATGGGTGGAGTATTTACGGTAAACTGCCCACTTGGCAGTACATCAAGTGTATCATATGCCAAGTACG
CCCCCTATTGACGTCAATGACGGTAAATGGCCCGCCTGGCATTATGCCCAGTACATGACCTTATGGGACTTTCCTACTTGGCAGTACATCTACGTATTAGTC
ATCGCTATTACCATGCTGATGCGGTTTTGGCAGTACATCAATGGGCGTGGATAGCGGTTTGACTCACGGGGATTTCCAAGTCTCCACCCCATTGACGTCAA
TGGGAGTTTGTTTTGGCACCAAAATCAACGGGACTTTCCAAAATGTCGTAACAACTCCGCCCCATTGACGCAAATGGGCGGTAGGCGTGTACGGTGGGAG
GTCTATATAAGCAGAGCTGGTTTAGTGAACCGTCAGATCAGATCTTTGTCGATCCTACCATCCACTCGACACACCCGCCAGCGGCCGCATGTACCCGTACG
ATGTCCCCGACTACGCCGGATCAGGCTCT

P10636-8_244_372

CAGACGGCGCCTGTTCCAATGCCTGACTTGAAGAACGTTAAATCCAAGATCGGGTCCACCGAAAACCTTAAGCACCAGCCCGGTGGAGGCAAGGTCCAG
ATTATCAACAAGAAGCTCGACCTTAGTAATGTTCAAAGCAAGTGTGGCTCCAAAGATAACATTAAACACGTCCCAGGAGGCGGGAGCGTGCAGATCGTGT
ACAAGCCCGTCGACCTGTCTAAGGTTACGTCCAAGTGTGGATCCCTGGGGAACATCCACCACAAACCTGGGGGCGGCCAGGTGGAGGTGAAGTCCGAAA
AGCTGGACTTTAAAGACCGAGTTCAGAGCAAAATTGGCTCCCTTGACAATATCACACACGTGCCTGGAGGAGGCAATAAGAAGATTGAGGGCAGTGGGT
CCGATTACAAAGACCATGATGGCGATTATAAGGACCACGACATCGATTATAAAGATGACGATGATAAATAGGTCGAGCCGCGGCAATTCCGACGTTACTG
GCCGAAGCCGCTTGGAATAAGGCCGGTGTGCGTTTGTCTATATGTTATTTTCCACCATATTGCCGTCTTTTGGCAATGTGAGGGCCCGGAAACCTGGCCCT
GTCTTCTTGACGAGCATTCCTAGGGGTCTTTCCCCTCTCGCCAAAGGAATGCAAGGTCTGTTGAATGTCGTGAAGGAAGCAGTTCCTCTGGAAGCTTCTTG
AAGACAAACAACGTCTGTAGCGACCCTTTGCAGGCAGCGGAACCCCCCACCTGGCAACAGGTGCCTCTGCGGCCAAAAGCCACGTGTATAAGATACACCT
GCAAAGGCGGCACAACCCCAGTGCCACGTTGTGAGTTGGATAGTTGTGGAAAGAGTCAAATGGCTCTCCTCAAGCGTATTCAACAAGGGGCTGAAGGAT
GCCCAGAAGGTACCCCATTGTATGGGATCTGATCTGGGGCCTCGGTGCACATGCTTTACATGTGTTTAGTCGAGGTTAAAAAAACGTCTAGGCCCCCCGA
ACCACGGGGACGTGGTTTTCCTTTGAAAAACACGATGATAATATGGTGTCAGTGATTAAGCCCGAGATGAAAATGAGATATTATATGGACGGGAGCGTCA
ATGGGCATGAGTTCACTATCGAAGGCGAGGGAACGGGACGCCCTTATGAGGGGCACCAAGAAATGACACTCAGAGTAACTATGGCAGAAGGCGGTCCA
ATGCCATTTGCATTCGATCTGGTTAGCCACGTATTTTGCTACGGGCATAGAGTTTTTACAAAATACCCCGAAGAGATCCCAGATTACTTTAAGCAGGCATTC
CCTGAAGGCCTGTCCTGGGAACGATCCCTGGAGTTCGAGGACGGGGGCAGCGCGAGTGTCTCCGCTCACATTAGTCTGAGGGGGAACACCTTTTATCATA
AATCTAAATTCACAGGAGTCAATTTCCCAGCAGATGGCCCAATTATGCAGAATCAGTCAGTTGATTGGGAGCCTTCTACGGAAAAGATCACTGCAAGCGA
CGGCGTGCTTAAGGGGGATGTCACCATGTATCTGAAACTGGAAGGGGGCGGAAACCATAAGTGTCAAATGAAAACTACCTATAAGGCCGCAAAGGAGA
TTCTCGAGATGCCCGGCGATCATTACATCGGTCATCGATTAGTTCGTAAGACCGAGGGAAATATCACTGAACAGGTCGAGGACGCTGTTGCACACTCATG
AGCTAGCTTGACTGACTGAGATACAGCGTACCTTCAGCTCACAGACATGATAAGATACATTGATGAGTTTGGACAAACCACAACTAGAATGCAGTGAAAA
AAATGCTTTATTTGTGAAATTTGTGATGCTATTGCTTTATTTGTAACCATTATAAGCTGCAATAAACAAGTTAACAACAACAATTGCATTCATTTTATGTTTC
AGGTTCAGGGGGAGGTGTGGGAGGTTTTTTAAAGCAAGTAAAACCTCTACAAATGTGGTATTGGCCCATCTCTATCGGTATCGTAGCATAACCCCTTGGG
GCCTCTAAACGGGTCTTGAGGGGTTTTTTGTGCCCCTCGGGCCGGATTGCTATCTACCGGCATTGGCGCAGAAAAAAATGCCTGATGCGACGCTGCGCGT
CTTATACTCCCACATATGCCAGATTCAGCAACGGATACGGCTTCCCCAACTTGCCCACTTCCATACGTGTCCTCCTTACCAGAAATTTATCCTTAAGGTCGTC
AGCTATCCTGCAGGCGATCTCTCGATTTCGATCAAGACATTCCTTTAATGGTCTTTTCTGGACACCACTAGGGGTCAGAAGTAGTTCATCAAACTTTCTTCCC
TCCCTAATCTCATTGGTTACCTTGGGCTATCGAAACTTAATTAACCAGTCAAGTCAGCTACTTGGCGAGATCGACTTGTCTGGGTTTCGACTACGCTCAGAA
TTGCGTCAGTCAAGTTCGATCTGGTCCTTGCTATTGCACCCGTTCTCCGATTACGAGTTTCATTTAAATCATGTGAGCAAAAGGCCAGCAAAAGGCCAGGA
ACCGTAAAAAGGCCGCGTTGCTGGCGTTTTTCCATAGGCTCCGCCCCCCTGACGAGCATCACAAAAATCGACGCTCAAGTCAGAGGTGGCGAAACCCGAC
AGGACTATAAAGATACCAGGCGTTTCCCCCTGGAAGCTCCCTCGTGCGCTCTCCTGTTCCGACCCTGCCGCTTACCGGATACCTGTCCGCCTTTCTCCCTTC
GGGAAGCGTGGCGCTTTCTCATAGCTCACGCTGTAGGTATCTCAGTTCGGTGTAGGTCGTTCGCTCCAAGCTGGGCTGTGTGCACGAACCCCCCGTTCAGC
CCGACCGCTGCGCCTTATCCGGTAACTATCGTCTTGAGTCCAACCCGGTAAGACACGACTTATCGCCACTGGCAGCAGCCACTGGTAACAGGATTAGCAG
AGCGAGGTATGTAGGCGGTGCTACAGAGTTCTTGAAGTGGTGGCCTAACTACGGCTACACTAGAAGAACAGTATTTGGTATCTGCGCTCTGCTGAAGCCA
GTTACCTTCGGAAAAAGAGTTGGTAGCTCTTGATCCGGCAAACAAACCACCGCTGGTAGCGGTGGTTTTTTTGTTTGCAAGCAGCAGATTACGCGCAGAA
AAAAAGGATCTCAAGAAGATCCTTTGATCTTTTCTACGGGGTCTGACGCTCAGTGGAACGAAAACTCACGTTAAGGGATTTTGGTCATGAGATTATCAAAA
AGGATCTTCACCTAGATCCTTTTAAATTAAAAATGAAGTTTTAAATCAATCTAAAGTATATATGAGTAAACTTGGTCTGACAGTTACCAATGCTTAATCAGT
GAGGCACCTATCTCAGCGATCTGTCTATTTCGTTCATCCATAGTTGCATTTAAATTTCCGAACTCTCCAAGGCCCTCGTCGGAAAATCTTCAAACCTTTCGTC
CGATCCATCTTGCAGGCTACCTCTCGAACGAACTATCGCAAGTCTCTTGGCCGGCCTTGCGCCTTGGCTATTGCTTGGCAGCGCCTATCGCCAGGTATTACT
CCAATCCCGAATATCCGAGATCGGGATCACCCGAGAGAAGTTCAACCTACATCCTCAATCCCGATCTATCCGAGATCCGAGGAATATCGAAATCGGGGCG
CGCCTGGCCTCCGCGCCGGGTTTTGGCGCCTCCCGCGGGCGCCCCCCTCGTCACGGCGAGCGCTGCCACGTCAGACGAAGGGCGCAGGAGCGTCCTGAT
CCTTCCGCCCGGACGCTCAGGACAGCGGCCCGCTGCTCATAAGACTCGGCCTTAGAACCCCAGTATCAGCAGAAGGACATTTTAGGACGGGACTTGGGTG
ACTCTAGGGCACTGGTTTTCTTTCCAGAGAGCGGAACAGGCGAGGAAAAGTAGTCCCTTCTCGGCGATTCTGCGGAGGGATCTCCGTGGGGCGGTGAAC
GCCGATGATTATATAAGGACGCGCCGGGTGTGGCACAGCTAGTTCCGTCGCAGCCGGGATTTGGGTCGCGGTTCTTGTTTGTGGATCGCTGTGATCGTCA
CTTGGTGAGTAGCGGGCTGCTGGGCTGGCCGGGGCTTTCGTGGCCGCCGGGCCGCTCGGTGGGACGGAAGCGTGTGGAGAGACCGCCAAGGGCTGTA
GTCTGGGTCCGCGAGCAAGGTTGCCCTGAACTGGGGGTTGGGGGGAGCGCAGCAAAATGGCGGCTGTTCCCGAGTCTTGAATGGAAGACGCTTGTGAG
GCGGGCTGTGAGGTCGTTGAAACAAGGTGGGGGGCATGGTGGGCGGCAAGAACCCAAGGTCTTGAGCCCTTCGCTAATGCGGGAAAGCTCTTATTCGG
GTGAGATGGGCTGGGCACCATCTGGGGACCCTGACGTGAAGTTTGTCACTGACTGGAGAACTCGGTTTGTCGTCTGTTGCGGGGGCGGCAGTTATGGCG
GTGCCGTTGGGCAGTGCACCCGTACCTTTGGGAGCGCGCGCCCTCGTCGTGTCGTGACGTCACCCGTTCTGTTGGCTTATAATGCAGGGTGGGGCCACCT
GCCGGTAGGTGTGCGGTAGGCTTTTCTCCGTCGCAGGACGCAGGGTTCGGGCCTAGGGTAGGCTCTCCTGAATCGACAGGCGCCGGACCTCTGGTGAGG
GGAGGGATAAGTGAGGCGTCAGTTTCTTTGGTCGGTTTTATGTACCTATCTTCTTAAGTAGCTGAAGCTCCGGTTTTGAACTATGCGCTCGGGGTTGGCGA
GTGTGTTTTGTGAAGTTTTTTAGGCACCTTTTGAAATGTAATCATTTGGGTCAATATGTAATTTTCAGTGTTAGACTTGTAAATTGTCCGCTAAATTCTGGCC
GTTTTTGGCTTTTTTGTTAGACAACATGACCGAGTACAAGCCCACGGTGCGCCTCGCCACCCGCGACGACGTCCCCAGGGCCGTACGCACCCTCGCCGCCG
CGTTCGCCGACTACCCCGCCACGCGCCACACCGTCGATCCGGACCGCCACATCGAGCGGGTCACCGAGCTGCAAGAACTCTTCCTCACGCGCGTCGGGCT
CGACATCGGCAAGGTGTGGGTCGCGGACGACGGCGCCGCGGTGGCGGTCTGGACCACGCCGGAGAGCGTCGAAGCGGGGGCGGTGTTCGCCGAGATC
GGCCCGCGCATGGCCGAGTTGAGCGGTTCCCGGCTGGCCGCGCAGCAACAGATGGAGGGCCTCCTGGCGCCGCACCGGCCCAAGGAGCCCGCGTGGTT
CCTGGCCACCGTCGGCGTCTCGCCCGACCACCAGGGCAAGGGTCTGGGCAGCGCCGTCGTGCTCCCCGGAGTGGAGGCCGCCGAGCGCGCCGGGGTGC
CCGCCTTCCTGGAGACCTCCGCGCCCCGCAACCTCCCCTTCTACGAGCGGCTCGGCTTCACCGTCACCGCCGACGTCGAGGTGCCCGAAGGACCGCGCAC
CTGGTGCATGACCCGCAAGCCCGGTGCCTAGGCTAGTATGTAAGCCTAGTCTTAGATAATAAAATCGCTATCCATCGAAGATGGATGTGTGTTGGTTTTTT
GTGTGTGTAACGCTAGGCGCGCCTGGTGTACCGAGAACGATCCTCTCAGTGCGAGTCTCGACGATCCATATCGTTGCTTGGCAGTCAGCCAGTCGGAATC
CAGCTTGGGACCCAGGAAGTCCAATCGTCAGATATTGTACTCAAGCCTGGTCACGGCAGCGTACCGATCTGTTTAAACCTAGATATTGATAGTCTGATCGG
TCAACGTATAATCGAGTCCTAGCTTTTGCAAACATCTATCAAGAGACAGGATCAGCAGGAGGCTTTCGCATGAGTATTCAACATTTCCGTGTCGCCCTTATT
CCCTTTTTTGCGGCATTTTGCCTTCCTGTTTTTGCTCACCCAGAAACGCTGGTGAAAGTAAAAGATGCTGAAGATCAGTTGGGTGCGCGAGTGGGTTACAT
CGAACTGGATCTCAACAGCGGTAAGATCCTTGAGAGTTTTCGCCCCGAAGAACGCTTTCCAATGATGAGCACTTTTAAAGTTCTGCTATGTGGCGCGGTAT
TATCCCGTATTGACGCCGGGCAAGAGCAACTCGGTCGCCGCATACACTATTCTCAGAATGACTTGGTTGAGTATTCACCAGTCACAGAAAAGCATCTTACG
GATGGCATGACAGTAAGAGAATTATGCAGTGCTGCCATAACCATGAGTGATAACACTGCGGCCAACTTACTTCTGACAACGATTGGAGGACCGAAGGAG
CTAACCGCTTTTTTGCACAACATGGGGGATCATGTAACTCGCCTTGATCGTTGGGAACCGGAGCTGAATGAAGCCATACCAAACGACGAGCGTGACACCA
CGATGCCTGTAGCAATGGCAACAACCTTGCGTAAACTATTAACTGGCGAACTACTTACTCTAGCTTCCCGGCAACAGTTGATAGACTGGATGGAGGCGGA
TAAAGTTGCAGGACCACTTCTGCGCTCGGCCCTTCCGGCTGGCTGGTTTATTGCTGATAAATCTGGAGCCGGTGAGCGTGGGTCTCGCGGTATCATTGCA
GCACTGGGGCCAGATGGTAAGCCCTCCCGTATCGTAGTTATCTACACGACGGGGAGTCAGGCAACTATGGATGAACGAAATAGACAGATCGCTGAGATA
GGTGCCTCACTGATTAAGCATTGGTAACCGATTCTAGGTGCATTGGCGCAGAAAAAAATGCCTGATGCGACGCTGCGCGTCTTATACTCCCACATATGCCA
GATTCAGCAACGGATACGGCTTCCCCAACTTGCCCACTTCCATACGTGTCCTCCTTACCAGAAATTTATCCTTAAGATCGTTTAAACTCGACTCTGGCTCTAT
CGAATCTCCGTCGTTTCGAGCTTACGCGAACAGCCGTGGCGCTCATTTGCTCGTCGGGCATCGAATCTCGTCAGCTATCGTCAGCTTACCTTTTTGGCAGCG
ATCGCGGCTCCCGACATCTTGGACCATTAGCTCCACAGGTATCTTCTTCCCTCTAGTGGTCATAACAGCAGCTTCAGCTACCTCTCAATTCAAAAAACCCCTC
AAGACCCGTTTAGAGGCCCCAAGGGGTTATGCTATCAATCGTTGCGTTACACACACAAAAAACCAACACACATCCATCTTCGATGGATAGCGATTTTATTA

TCTAACTGCTGATCGAGTGTAGCCAGATCTAGTAATCAATTACGGGGTCATTAGTTCATAGCCCATATATGGAGTTCCGCGTTACATAACTTACGGTAAAT
GGCCCGCCTGGCTGACCGCCCAACGACCCCCGCCCATTGACGTCAATAATGACGTATGTTCCCATAGTAACGCCAATAGGGACTTTCCATTGACGTCAATG
GGTGGAGTATTTACGGTAAACTGCCCACTTGGCAGTACATCAAGTGTATCATATGCCAAGTACGCCCCCTATTGACGTCAATGACGGTAAATGGCCCGCCT
GGCATTATGCCCAGTACATGACCTTATGGGACTTTCCTACTTGGCAGTACATCTACGTATTAGTCATCGCTATTACCATGCTGATGCGGTTTTGGCAGTACA
TCAATGGGCGTGGATAGCGGTTTGACTCACGGGGATTTCCAAGTCTCCACCCCATTGACGTCAATGGGAGTTTGTTTTGGCACCAAAATCAACGGGACTTT
CCAAAATGTCGTAACAACTCCGCCCCATTGACGCAAATGGGCGGTAGGCGTGTACGGTGGGAGGTCTATATAAGCAGAGCTGGTTTAGTGAACCGTCAG
ATCAGATCTTTGTCGATCCTACCATCCACTCGACACACCCGCCAGCGGCCGCATGTACCCGTACGATGTCCCCGACTACGCCGGATCAGGCTCT

Supplementary References

1 Louros, N., Orlando, G., De Vleeschouwer, M., Rousseau, F. & Schymkowitz, J. Structure-based machine-guided mapping of amyloid sequence space reveals uncharted sequence clusters with higher solubilities. *Nat Commun* **11**, 3314, doi:10.1038/s41467-020-17207-3 (2020).

2 Schymkowitz, J. *et al.* The FoldX web server: an online force field. *Nucleic Acids Res* **33**, W382-388, doi:10.1093/nar/gki387 (2005).
